# Effectiveness of Remotely Delivered Interventions to Simultaneously Optimize Management of Hypertension, Hyperglycemia and Dyslipidemia in People With Diabetes: A Systematic Review and Meta-Analysis of Randomized Controlled Trials

**DOI:** 10.3389/fendo.2022.848695

**Published:** 2022-03-15

**Authors:** Malindu E. Fernando, Leonard Seng, Aaron Drovandi, Benjamin J. Crowley, Jonathan Golledge

**Affiliations:** ^1^ Queensland Research Centre for Peripheral Vascular Disease, College of Medicine and Dentistry, James Cook University, Townsville, QLD, Australia; ^2^ Ulcer and Wound Healing Consortium (UHEAL), Australian Institute of Tropical Health and Medicine, James Cook University, Townsville, QLD, Australia; ^3^ Faculty of Health and Medicine, School of Health Sciences, University of Newcastle, Newcastle, NSW, Australia; ^4^ Australian Institute of Tropical Health and Medicine, James Cook University, Townsville, QLD, Australia; ^5^ Department of Vascular and Endovascular Surgery, Townsville University Hospital, Townsville, QLD, Australia

**Keywords:** blood pressure, cholesterol, lipids, systematic review, telehealth

## Abstract

**Background:**

Remotely delivered interventions may be more efficient in controlling multiple risk factors in people with diabetes.

**Purpose:**

To pool evidence from randomized controlled trials testing remote management interventions to simultaneously control blood pressure, blood glucose and lipids.

**Data Sources:**

PubMed/Medline, EMBASE, CINAHL and the Cochrane library were systematically searched for randomized controlled trials (RCTs) until 20^th^ June 2021.

**Study Selection:**

Included RCTs were those that reported participant data on blood pressure, blood glucose, and lipid outcomes in response to a remotely delivered intervention.

**Data Extraction:**

Three authors extracted data using a predefined template. Primary outcomes were glycated hemoglobin (HbA1c), total cholesterol (TC), low-density lipoprotein cholesterol (LDL-c), systolic and diastolic blood pressure (SBP & DBP). Risk of bias was assessed using the Cochrane collaboration RoB-2 tool. Meta-analyses are reported as standardized mean difference (SMD) with 95% confidence intervals (95%CI).

**Data Synthesis:**

Twenty-seven RCTs reporting on 9100 participants (4581 intervention and 4519 usual care) were included. Components of the remote management interventions tested were identified as patient education, risk factor monitoring, coaching on monitoring, consultations, and pharmacological management. Comparator groups were typically face-to-face usual patient care. Remote management significantly reduced HbA1c (SMD -0.25, 95%CI -0.33 to -0.17, p<0.001), TC (SMD -0.17, 95%CI -0.29 to -0.04, p<0.0001), LDL-c (SMD -0.11, 95%CI -0.19 to -0.03, p=0.006), SBP (SMD -0.11, 95%CI -0.18 to -0.04, p=0.001) and DBP (SMD -0.09, 95%CI -0.16 to -0.02, p=0.02), with low to moderate heterogeneity (I²= 0 to 75). Twelve trials had high risk of bias, 12 had some risk and three were at low risk of bias.

**Limitations:**

Heterogeneity and potential publication bias may limit applicability of findings.

**Conclusions:**

Remote management significantly improves control of modifiable risk factors.

**Systematic Review Registration:**

[https://www.crd.york.ac.uk/prospero/display_record.php?RecordID=258433], identifier PROSPERO (CRD42021258433).

## Introduction

Adults diagnosed with diabetes are at high risk of major adverse events such as myocardial infarction, stroke, end stage renal failure, foot ulceration, amputation and death (1, 2). The risk of these complications can be reduced by control of blood glucose, blood pressure and lipids (3–7). Optimal control of these risk factors is infrequently achieved in routine practice, representing a missed opportunity to prevent major adverse events (6, 8). This may be due to limited access to specialists, lack of cohesive healthcare delivery and ineffective patient education (9, 10).

The medical management of people with diabetes usually involves frequent face-to-face appointments with multiple specialists (11). This can contribute to confusion about how intensively risk factors should be controlled and who is responsible for managing these risk factors (12). It also disadvantages patients in rural and remote settings who may not be able to access specialist medical services easily (13).

Remotely delivered risk factor management programs have been proposed as a more efficient way to control multiple risk factors (14–18). Risk factor monitoring, healthcare consultations, medication prescription and behavioral support can occur remotely to facilitate optimizing blood glucose, blood pressure and lipids (17–19). No previous meta-analysis or overviews have evaluated the benefit of simultaneous remote management of all these risk factors (17, 18, 20). Evidence on the effectiveness of interventions that simultaneously control multiple modifiable risk factors is needed to inform how most efficiently to deliver preventive management. This systematic review and meta-analysis aimed to pool evidence from randomized controlled trials (RCTs) testing the effectiveness of remote risk factor management programs for people with diabetes in simultaneously controlling blood glucose, blood pressure, and lipids.

## Methods

This review was conducted in accordance with the Preferred Reporting Items for Systematic Reviews and Meta-Analyses (PRISMA) guidelines (21), and is registered with PROSPERO (CRD42021258433).

### Data Sources and Searches

The PubMed/Medline, EMBASE, CINAHL and Cochrane library databases were searched independently by three authors (MF, LS AD) for English language articles of RCTs published from 1^st^ January 2000 to 20^th^ June 2021. This date restriction was applied due to the relative recent introduction of remotely-delivered healthcare and in order to provide a contemporary assessment of intervention strategies. The search combined three term groups; 1) ‘controlled trial’ (e.g. randomized, clinical trial), 2) ‘remote’ (e.g. telehealth), and 3) ‘disease and treatment’ (e.g. diabetes, dyslipidemia). The full search string is shown in **Supplementary Text 1**. Reference and citation lists of eligible articles were also manually searched.

### Study Selection

Eligible articles were published RCTs that evaluated the effect of remote medical management interventions in comparison to usual care. The population of interest were adults ≥18 years old with either type 1 or type 2 diabetes mellitus irrespective of disease duration or history of cardiovascular disease. The interventions were remotely delivered healthcare (e.g. internet or phone-based monitoring or telehealth consultations) aimed at optimizing glycemic control, systolic blood pressure (SBP) and/or diastolic blood pressure (DBP) and total cholesterol (TC) and/or low-density lipoprotein cholesterol (LDL-c). The control group received usual medical management without remotely delivered healthcare. Each RCT identified was screened by at least two authors (MF, LS, AD, BC). Trials that did not aim to control all three risk factors or failed to report them were excluded.

### Data Extraction and Quality Assessment

The primary outcome was the impact of the remotely delivered interventions on: 1) hemoglobin A1c (HbA1c %), 2) TC and LDL-c (mmol/L), and 3) SBP and DBP (mmHg) compared to the control groups. Secondary outcomes included incidence of adverse events including hypoglycemia, postural hypotension, hospital admission, death, limb-related events including leg revascularization or lower limb amputation, other medication related side-effects, major adverse cardiovascular events (MACE), development of micro-vascular complications including progression of retinopathy, neuropathy (including incident foot ulceration), or nephropathy, and all-cause mortality. Other secondary outcomes were health-related quality of life and cost-benefit analyses.

A standardized data extraction form was developed to extract the following data from each study: title, authors, year published, country of publication, number of participants, participant characteristics, intervention setting, type, frequency and duration of remote and usual care intervention(s), primary and secondary outcomes, study limitations and whether intention-to-treat or per-protocol analyses. Two authors independently extracted data, which were checked by a third author (MF, LS, AD, BC). Where studies reported multiple follow-up data, the longest follow-up duration was used. Where there were more than one intervention arm, all intervention groups were included. Meta-analyses included the number of participants completing the trial rather than numbers initially randomized as outcome data were only available for this group. Study authors were contacted for all potentially eligible studies to obtain additional and missing data.

Methodological quality was assessed independently by three authors (AD, LS and BC) using the Cochrane collaborations revised risk-of-bias tool for randomized trials (RoB 2) (22). Following independent evaluation, discussions were held between assessors to arrive at a consensus score. Where this was not possible, a final consensus on the overall risk of bias was made by an independent fourth assessor (MF). In relation to the tool, five outcomes were possible for each criterion which were ‘yes’, ‘probably yes’, ‘no information’, ‘probably no’, or ‘no’ (22). Studies were rated as low risk of bias if all domains were judged to be at low risk of bias, high risk of bias if any domain was judged to be at high risk of bias, or ‘some concerns’ of bias if any domain was judged to have some concerns but no domain had a high risk of bias (22).

### Data Synthesis and Analysis

Numerical data were reported as mean and standard deviation (SD) and categorical data as number and percentage (%). Meta-analysis were performed for any primary or secondary outcome with data extractable from a minimum of three studies. The meta-analyses were conducted using the inverse-variance method for continuous outcomes and the Mantel-Haenszel statistical method for dichotomous outcomes with random effect models anticipating substantial heterogeneity (23). The results were reported as standardized mean difference (SMD) (24) or risk ratio (RR) and 95% CI for dichotomous outcomes (23). All statistical tests were two-sided and a p value <0.05 was considered significant. Heterogeneity was assessed using I^2^ statistic values (interpreted as 0 to 49%: low, 50 to 74%: moderate and 75 to 100%: high) (25). Several sensitivity and subgroup analyses were carried out including leave-one-out (LOO) sensitivity analyses and analysis excluding studies with high risk of bias. Several sub-group analyses were also carried out (see **Supplementary Text 2**). Five distinct aspects of the remote management programs tested were defined to clarify which aspects of the interventions were most important in improving outcome in subgroup meta-analysis. Subgroup meta-analysis was also planned to evaluate whether remote management was more effective in studies that only included a higher risk population at entry who were at greater risk of MACE. Higher-risk was defined as: a documented history of cardiovascular disease, a diabetes duration of greater than 10 years, HbA1c of >10.0% and/or LDL of >2.0 mmol/L and/or SBP of >130 mmHg and/or a DBP of >80 mmHg or a previous history of diabetes related complications at entry. Publication bias was assessed by funnel plots comparing the summary estimate of each study and its precision (1/standard error) (26). All analyses were conducted with Review Manager (RevMan) version 5.4. (The Cochrane Collaboration, 2020).

## Results

**Figure 1:**
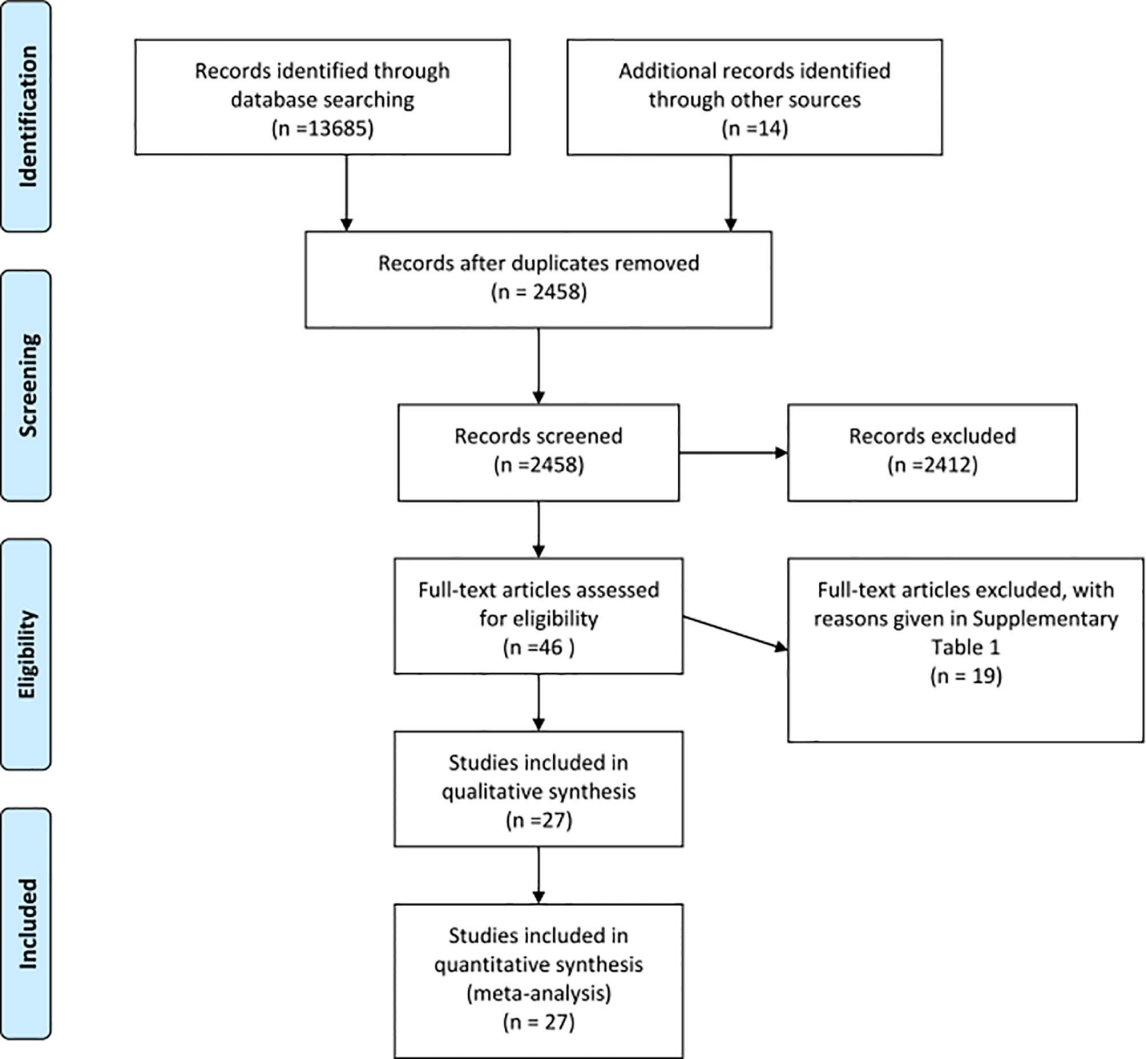
PRISMA flow diagram of the search results and number of eligible articles included.

Of 2458 unique articles identified, 46 were assessed for full-text eligibility and 27 RCTs were included (**Figure 1**) (27–53). Most full-text screened studies that were excluded did not target or report on the impact of the remotely-delivered intervention on all key risk factors of interest (**Supplementary Table 1**). Of 33 contacted authors from potentially eligible studies, four replied with the request for additional data (32, 37, 39, 44).

### Study and Participant Characteristics

The included studies had a total of 9153 participants randomized and reported outcomes on 4581 participants randomized to an intervention group and 4519 to usual care. Sample sizes of individual trials ranged from 36 to 1665 (**Table 1**) and follow-up durations ranged from 3 to 60 months. **Supplementary Table 2** reports the inclusion and exclusion criteria of each trial and the total number of participants screened and excluded. Six RCTs included people with type 1 diabetes (27, 29, 31, 33, 38, 46) and all others exclusively included participants with type 2 diabetes. Several studies excluded participants with severe complications such as foot ulcers, progressive nephropathy or retinopathy. Three studies recruited participants deemed to be at high risk of diabetes complications based on entry criteria (35, 41, 51). Participant medications at baseline and follow-up are shown in **Supplementary Table 3**, and baseline risk factors are shown in **Supplementary Table 4**, and study outcome measures, additional supports and methods of risk factor monitoring are shown in **Supplementary Table 5**.

**Table 1:** Characteristics of study participants in included randomized controlled trials (n=27).

TOTAL COHORT								INTERVENTION GROUP					CONTROL GROUP				
Study	Country	Study setting	Design	Number randomized	Attrition	Follow-up duration	Population description	Type of remote intervention tested	N	Age	Females	Diabetes duration	Control group description	N	Age	Females	Diabetes duration
Aytekin Kanadli (27)	Turkey	Hospital	Two-arm RCT	91	3/91	3-months	People with diabetes attending an endocrinology unit	Telephone-based education and monitoring	44	NR	27 (61.4%)	NR	Routine treatment and care	44	NR	29 (65.9%)	NR
Blackberry (28)	Australia	Primary care/ community	Stratified cluster RCT	473	22/473	18-months	Patients with poorly controlled type 2 diabetes	Practice nurse led telephone coaching	236	63.6 (10.4)	109 (46.0%)	10 [5-15]	Usual general practice care	237	61.9 (10.5)	95 (40.0%)	9 [5-13]
Bond (29)	USA	Hospital	Two-arm RCT	62	NR	6-months	People ≥60 years with diabetes	Web-based education and monitoring program	31	66.2 (5.7)	13 (41.9%)	16.1 (10.5)	Standard diabetes care	31	68.2 (6.2)	15 (48.4%)	17.8 (11.7)
Crowley (30)	USA	Primary care/ community	Two-arm parallel group RCT	359	29/359	12-months	African American patients with type 2 diabetes	Nurse-administered telephone intervention	182	56.0 (12.0)	126 (69.0%)	NR	Usual care	177	57.0 (12.0)	133 (75.0%)	NR
Davis (31)	USA	Primary care/ community	Non-blinded, two-arm, parallel-group single-site RCT	165	NR	12-months	People ≥35 years with uncontrolled diabetes	Education through videoconferencing	85	59.9 (9.4)	62 (72.9%)	8.5 (6.6)	Usual care	80	59.2 (9.3)	61 (76.3%)	10.3 (8.1)
de Vasconcelos (40)	Brazil	Primary care/ community	Parallel group RCT	36	5/36	6-months	Patients with type 2 diabetes	Health tele-coaching programme *via* telephone	18	60.9 (NR)	14 (58.3%)	10 (8.5)	Usual care	18	59.6 (NR)	10 (41.7%)	8.67 (6.4)
Eakin (32)	Australia	Primary care/ community	Non-blinded, two-arm, parallel-group, pragmatic RCT	302	53/302	24-months	People with type 2 diabetes and physically inactive or overweight	Telephone-based weight and activity intervention	151	57.7 (8.1)	67 (44.4%)	4.0 [2.0-7.0]	Usual care & mailed results	151	58.3 (9.0)	65 (43.0%)	5.0 [2.0-10.0]
Harno (33)	Finland	Mixed primary care & hospital	Two-arm, parallel-group, multi-center RCT	175	NR	12-months	People with diabetes	E-health app and diabetes management system and text messaging	101	NR	NR	NR	Usual care	74	NR	NR	NR
Holbrook (34)	Canada	Primary care/ community	Two-arm, pragmatic RCT	511	66/511	6-months	People with type 2 diabetes	Web-based diabetes risk factor tracker & education	253	61.0 (13.1)	130 (51.4%)	8.7 (9.0)	Usual care	258	60.5 (11.9)	122 (47.3%)	10.0 (10.7)
Huo (35)	China	Hospital	Single-blinded, parallel-group multi-center, RCT	502	34/502	6-months	People diagnosed with type 2 diabetes and CHD within the prior 3 years	Text-messaging behavior support	251	59.5 (9.4)	43 (17.1%)	NR	Standard care only	251	59.5 (9.1)	45 (17.9%)	NR
Kempf (36)	Germany	Mixed primary care & hospital	Single-blinded, two-arm, parallel-group, single-center RCT	202	69/202	12-months	Type 2 diabetes with poor control (HbA1c >7.5%), BMI >27 kg/m^2^, and two oral medications	Web-portal and remote monitoring and telephone calls	102	NR	48 (47.0%)	NR	Standard care and limited home-based monitoring	100	NR	41 (41.0%)	NR
Krein (37)	USA	Primary care/ community	Two-arm, multi-site RCT	246	30/246	18-months	Patients with poorly controlled type 2 diabetes	Nurse practitioner-led telephone-based case management	123	61 (10.0)	2 (2.0%)	11 (10.0)	Usual care	123	61 (11.0)	6 (5.0%)	11 (9.0)
Leichter (38)	USA	Primary care/ community	Non-blinded, two-arm, parallel-group, single-center RCT	98	28/98	12-months	People with diabetes	Computer based monitoring and phone-based consultations	49	45.5 (11.8)	24 (49.0%)	NR	In-clinic consultations	49	50.9 (11.7)	19 (38.8%)	NR
Lim (39)	Singapore	Mixed primary care & hospital	1:1 parallel group multi-center RCT	204	9/204	6-months	Asian patients with type 2 diabetes	Smartphone application and remote coaching	99	50.8 (10.0)	39 (37.1%)	4.2 (3.6)	Usual care	105	51.6 (9.4)	33 (33.3%)	5.2 (4.5)
Liou (40)	Taiwan	Primary care/ community	Two-arm, multi-center RCT	95	NR	6-months	People with type 2 diabetes and HbA1c >7% for >1 year	Internet-based education program and video conferencing education program	54	56.6 (7.7)	26 (48.1%)	NR	Usual care	41	57.0 (7.5)	21 (51.2%)	NR
Nicolucci (41)	Italy	Primary care/ community	Non-blinded, two-arm, Parallel-group, multi-center RCT	302	53/302	12-months	People >45 years with type 2 diabetes and HbA1c between 7.5 and 10%, and SBP >130mmHg	Monitoring and education program delivered *via* telephone	153	59.1 (10.3)	59 (38.6%)	8.3 (6.2)	Usual practice	149	57.8 (8.9)	57 (38.3%)	8.7 (6.2)
Odnoletkova (42)	Belgium	Primary care/ community	Non-blinded, two-arm, parallel-group RCT	574	88/574	18-months	People with type 2 diabetes receiving anti-diabetic therapy	Nurse-led telephone coaching and pre-made education material	287	63.8 (8.7)	114 (39.7%)	NR	Usual care	287	62.4 (8.9)	107 (37.3%)	NR
Quinn (43)^*^	USA	Primary care/ community	Multi-Arm cluster RCT	213	50/213	12-months	Patients aged 18 to 64 with type 2 diabetes	Mobile and web-based self-management patient coaching system and provider decision support *via* telephone	23^‡^	52.8 (8.0)	11 (47.8%)	7.7 (5.6)	Usual care	56	53.2 (8.4)	28 (50%)	9.0 (7.0)
									22^§^	53.7 (8.2)	12 (54.5%)	6.8 (4.9)					
									62||	52.0 (8.0)	31 (50.0%)	8.2 (5.3)					
Ramallo-Farina (44)	Spain	Primary care/ community	Open-label multi-center cluster RCT	1123	NR	24-months	Patients with type 2 diabetes	Web-based platform and mobile text messaging	537	55.9 (7.0)	253 (47.1%)	8.4 (6.8)	Usual care	586	55.2 (7.3)	300 (51.2%)	8.6 (6.8)
Shahid (45)	Pakistan	Hospital	Two-arm parallel group RCT	440	NR	4-months	Patients with type 2 diabetes living in rural areas	Telephone coaching delivered by mobile phone	220	49.0 (8.8)	85 (38.6%)	NR	Usual care	220	49.21 (7.92)	85 (38.6%)	NR
Shea (46)	USA	Primary care/ community	Non-blinded, parallel-group, two-arm, multi-center RCT	1665	872/1665	60-months	People with diabetes aged over 55 years in medically underserved areas	Case management *via* remote education and home telemedicine unit for videoconference	844	70.8 (6.5)	536 (63.5%)	11.2 (9.6)	Usual care	821	70.9 (6.8)	510 (62.1%)	11.0 (9.2)
Tang (47)	USA	Primary care/ community	Parallel group RCT	415	36/415	12-months	Patients with uncontrolled type 2 diabetes	Online diabetes management system	202	54.0 (10.7)	83 (41.1%)	NR	Usual care	213	53.5 (10.2)	83 (39.0%)	NR
Varney (48)^*^	Australia	Hospital	Non-blinded, parallel-group, single-center RCT	94	23/94	12-months	People with type 2 diabetes and HbA1c > 7%	Telephone coaching	47	59 (10.5)	13 (27.7%)	12.6 (8.4)	Usual care	47	64 (8.7)	17 (36.2%)	13.1 (8.6)
Vinitha (50)	India	Hospital	Double blinded (investigator & outcome assessor), parallel-group, Multi-center RCT	248	30/248	24-months	Newly diagnosed people with type 2 diabetes with (HbA1c) > 6.5%, who were treatment naïve.	Text-messaging behavior support	126	42.4 (8.5)	40 (31.7%)	NR	Standard care	122	44.1 (8.9)	40 (32.8%)	NR
Wild (51)	UK	Primary care/ community	Single blinded, parallel-group, multi-center RCT	321	12/321	9-months	People with type 2 diabetes and HbA1c >7.5%	Telemonitoring & support *via* web-portal	160	60.5 (9.8)	54 (33.8%)	7.4 (5.7)	Usual care	161	61.4 (9.8)	53 (32.9%)	7.4 (5.8)
Yoo (52)^†^	South Korea	Mixed primary care & hospital	Open-label multi-site RCT	123	12/123	3-months	Overweight patients with type 2 diabetes and hypertension	Online data monitoring system and physician feedback *via* text-message	62	57.0 (9.1)	27 (47.4%)	6.0 (5.4)	Usual care	61	59.4 (8.4)	19 (35.2%)	7.2 (6.0)
Zhou (53)^†^	China	Hospital	Two-arm parallel group RCT	114	6/114	3-months	Patients with type 2 diabetes	Diabetes telemedicine system and data monitoring and feedback *via* internet, text or telephone	57	NR	NR	NR	Usual care	57	NR	NR	NR

Data are presented as n (%), mean (standard deviation SD), or median [interquartile range] unless otherwise specified. BMI; body mass index, CHD; coronary heart disease; NR; not reported, HbA1c; glycated hemoglobin, RCT; randomized controlled trial* Where the SD was not reported and instead the 95% confidence intervals (CIs) were reported, these were converted to SD using the equation SD= √N x (upper limit 95% CI-lower limit 95% CI)/3.92. Where only the standard error (SE) was reported, this was converted to SD by using the formula: SD = SE x√N. ^†^Reported baseline characteristics for a subset of the randomised cohort only (those who completed the trial). Ramello-Farina et al. (44) had several interventional groups and only the patient intervention group were included. Quinn et al. (2011) had three intervention groups: ^‡^intervention group a: online coaching only, ^§^group b: coaching and primary care providers portal, and || group c: coach PCP portal with decision-support.

### Description of the Types of Interventions Tested

A detailed description of the types of interventions is given in **Supplementary Table 5**, with **Supplementary Table 6** providing a summary overview of the key elements of the intervention provided in each trial used in the meta-analyses. Twenty out of the twenty-seven RCTs provided remote patient education without any in-person education (28–31, 35–37, 39–43, 45–50, 52, 53), nineteen RCTs provided remote risk factor monitoring (29, 31–34, 36–39, 41–47, 51–53), twenty-two RCTs provided remote coaching regarding risk factors without the use of in-person coaching (27–37, 39, 41, 43–44, 46–49, 51–53), twelve provided remote consultation without any in-person consultation (31, 35, 36, 40–47, 49) and fourteen provided remote pharmacological advice or reminders to the patient or treating team (28, 30, 34, 37–38, 41–44, 46–48, 50, 51) (see **Supplementary Text 3** for further info).

### Description of Control Groups

In most studies, the control group received usual care (**Supplementary Table 5**). This typically consisted of regular primary care physician management of participant risk factors based on guideline recommendations. Ten RCTs failed to provide a definition of usual care (27, 30, 35, 37, 39, 44, 47, 50, 52, 53). One RCT delivered non-health related text-messages to the control group (35). One RCT provided the control arm with the same blood glucose monitoring system as their intervention group, but the data was not transmitted to an online portal for further intervention (38).

### Risk of Bias of Included Studies

Overall, 12 trials were deemed to be at high risk (27, 29, 33, 36, 38, 40–42, 45, 49, 51, 53), 12 trials had some concerns regarding their risk of bias (30–32, 35, 37, 39, 43, 46–48, 50, 52) and three were at low risk of bias (**Supplementary Table 7**) (28, 34, 44). Problems identified with high-risk studies included lack of detail on the appropriateness of analyses (27, 29), lack of allocation concealment (45, 51), differences in baseline risk factors (HbA1c) between the intervention and control groups (33), missing outcome data (36, 41, 42, 49) and differences in how data were collected between the intervention and control groups for the primary outcome (38, 40, 53).

### Primary Outcome Measures

**Supplementary Table 8** reports on the main risk factor outcomes based on last known follow-up included in the meta-analysis.

#### Impact of Remote Intervention on HbA1c

**Figure 2:**
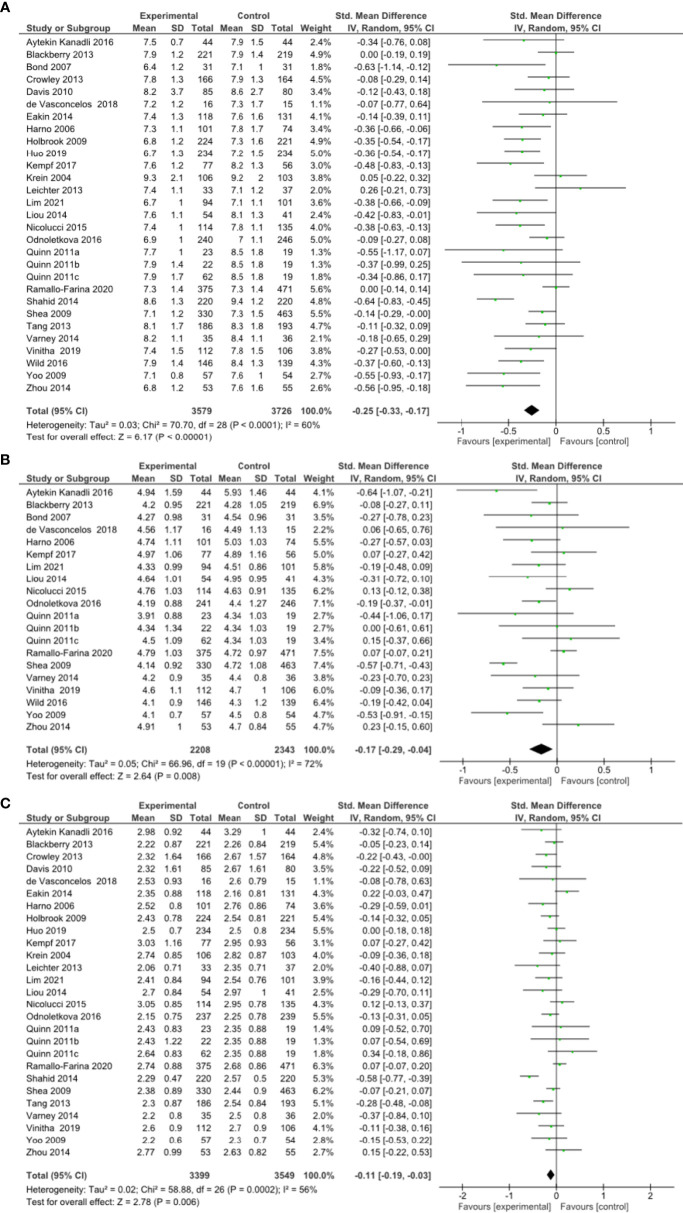
**(A)** Forest plot showing the effect of remote risk factor management on HbA1c, **(B)** Forest plot showing the effect of remote management on total cholesterol, **(C)** Forest plot showing the effect of remote risk factor management on LDL-cholesterol.

A meta-analysis of all 27 RCTs incorporating 3579 participants in the intervention group and 3726 participants in the control group found that remote risk factor management significantly reduced HbA1c compared to usual care (SMD -0.25, 95% CI -0.33 to -0.17, Z=6.17, p=<0.001; **Figure 2A**) with a moderate degree of heterogeneity (I²= 60%). The funnel plot was asymmetrical (**Supplementary Results, Figure 1.1**). LOO sensitivity analyses suggested removal of any individual RCT did not affect the significance of the finding (**Supplementary Results, Table 2.1**). Exclusion of studies with high risk of bias did not change the significance of the outcome (**Supplementary Results, Figure 3.1**). Subgroup analyses focused on remote intervention type or a high-risk population did not change the significance of the outcome (**Table 2** and **Supplementary Results, Figures 4.1-4.5 and 5.1**).

**Table 2:** Meta-analysis outcomes by subgroups of remote interventions and high-risk population.

Risk factor	SUBGROUP ANALYSIS OF REMOTE INTERVENTION TYPES					SUBGROUP ANALYSIS OF HIGH-RISK POPULATION AT ENTRY
	PATIENT EDUCATION	MONITORING OF RISK FACTORS	COACHING REGARDING RISK FACTOR MODIFICATION	CONSULTATION	PHARMACOLOGICAL MANGEMENT	
**HbA1c**	(SMD -0.26, 95% CI -0.35 to -0.17), Z=5.51, p<0.0001 (I²= 59%)	(SMD -0.27, 95% CI -0.37 to -0.17), Z=5.32, p<0.0001 (I²= 67%)	(SMD -0.24, 95% CI -0.32 to -0.16), Z=5.98, p<0.0001 (I²= 49%)	(SMD -0.19, 95% CI -0.29 to -0.10), Z=3.96, p=0.0001 (I²=29%)	(SMD -0.14, 95% CI -0.23 to -0.06), Z=3.36, p = 0.0008 (I²=48%)	(SMD -0.39, 95% CI -0.53 to -0.25), Z=5.52, p <0.0001 (I²= 0%)
**TC**	(SMD -0.14, 95% CI -0.29 to -0.01), Z=1.89, p=0.06 (I²=71%)	NA	(SMD -0.18, 95% CI -0.34 to -0.02), Z=2.23 p=0.03 (I²= 72%)	(SMD -0.15, 95% CI -0.35 to 0.05), Z=1.48, p=0.14) (I²= 75%)	(SMD -0.13, 95% CI -0.31 to 0.06), Z=1.35, p=0.18 (I²= 84%)	NA
**LDL-c**	(SMD -0.09, 95% CI -0.19 to -0.00), Z=2.01, p=0.04 (I²=58%)	NA	(SMD -0.06, 95% CI -0.13 to 0.00), Z=1.853, p=0.06 (I²=30%)	(SMD -0.10, 95% CI -0.18 to -0.02), Z=2.54, p=0.01 (I²=8%)	(SMD -0.19, 95% CI -0.17 to -0.01), Z=2.26, p=0.02 (I²=37%)	(SMD 0.02, 95% CI -0.12 to 0.15), Z=0.23, p=0.82 (I²=0%)
**SBP**	(SMD -0.10, 95% CI -0.18 to -0.01), Z=2.19, p=0.3 (I²=53%)	(SMD -0.12, 95% CI -0.19 to -0.04), Z=2.97, p=0.003 (I²=13%)	(SMD -0.09, 95% CI -0.17 to -0.02), Z=2.41, p=0.02 (I²=46%)	(SMD -0.10, 95% CI -0.18 to -0.03), Z=2.60, p=0.009 (I²=7%)	(SMD -0.13, 95% CI -0.21 to -0.04), Z=2.97, p=0.003) (I²=48%)	(SMD 0.08, 95% CI -0.05 to 0.22), Z=1.19, p=0.24 (I²=0%)
**DBP**	(SMD -0.07, 95% CI -0.17 to 0.03), Z=1.41 p=0.16 (I²=48%)	(SMD -0.14, 95% CI -0.26 to -0.02), Z=2.32, p=0.02 (I²=60%)	(SMD -0.13, 95% CI -0.20 to -0.05), Z=3.26, p=0.001 (I²=36%)	(SMD -0.07, 95% CI -0.19 to 0.04), Z=1.23, p=0.22 (I²=52%)	(SMD -0.12, 95% CI -0.18 to -0.06), Z=3.80, p=0.0001 (I²= 4%)	NA

Five distinct aspects of the remote management programs tested were defined in an attempt to clarify which aspects of the interventions were most important in improving outcome: 1) patient education, 2) monitoring of risk factors, 3) coaching to improve risk factor control, 4) health care professional telehealth consultation and 5) pharmacological management. We only included remote risk factor monitoring RCTs in a meta-analysis where either blood pressures, blood glucose or blood lipids were remotely monitored. Sub-group meta-analyses (MA) were performed for any primary outcome with data available from a minimum of three studies per remote intervention component. If the component of the intervention was not delivered remotely, this study was excluded from meta-analysis. HbA1c =glycated hemoglobin A1c, TC = total cholesterol, LDL-c = low density lipoprotein cholesterol, SBP= systolic blood pressure, DBP= diastolic blood pressure, SMD= standardized mean difference, 95% CI= 95% confidence interval and I²= measure of statistical heterogeneity. Subgroup meta-analysis was also planned to evaluate whether remote management was more effective in studies which only included a higher risk population defined as; a documented history of cardiovascular disease, a diabetes duration of greater than 10 years, HbA1c of > 10.0% (54) and/or LDL of >2.0 mmol/L (55) and/or systolic blood pressure of > 130 mmHg and/or a diastolic blood pressure of >80 mmHg or a previous history of diabetes related complications at entry. Green squares indicate where the subgroup meta-analysis outcome was statistically significant, and the red squares indicate where it was not and the yellow squares indicate where meta-analysis was not possible. The full results are reported in **Supplementary Results**.

#### Impact of Remote Intervention on Blood Lipids

A meta-analysis of 18 RCTs incorporating 2208 participants in the intervention group and 2343 participants in the control group found that remote risk factor management significantly reduced TC compared to usual care (SMD -0.17, 95% CI -0.29 to -0.04, Z=2.64, p=0.008) with a moderate degree of heterogeneity (I²= 72%) (**Figure 2B**). The funnel plot was asymmetrical (**Supplementary Results, Figures 1.2**). LOO sensitivity analyses suggested removal of one study reduced the heterogeneity and effect size substantially (46) (**Supplementary Results, Figures 2.2**). Exclusion of studies with high risk of bias changed the significance of the outcome (SMD -0.20, 95% CI -0.40 to 0.00, Z=1.91, p=0.06) with a high degree of heterogeneity (I²= 81%) and the funnel plot was symmetrical (**Supplementary Results, Figures 3.3, 3.4**). Subgroup analyses of remote interventions showed only the coaching of risk factor modification significantly reduced TC compared to usual care (**Table 2** and **Supplementary Results, Figures 4.6-4.10**). There were insufficient studies to undertake subgroup analysis of a high-risk population.

A meta-analysis of 25 RCTs incorporating 3399 participants in the intervention group and 3549 participants in the control group found that remote risk factor management significantly reduced LDL-c compared to usual care (SMD -0.11, 95% CI -0.19 to -0.03, Z=2.78, p=0.006) with a moderate degree of heterogeneity (I²= 56%) (**Figure 2C**). The funnel plot was asymmetrical (**Supplementary Results, Figures 1.3**). LOO sensitivity analyses suggested removal of any individual RCT did not affect the significance of the main finding (**Supplementary Results, Table 2.3**). Exclusion of studies with high risk of bias did not change the significance of the outcome (**Supplementary Results, Figure 3.5**). Subgroup analyses suggested that patient education, consultation and pharmacological management but not coaching of risk factor modification significantly reduced LDL-c compared to usual care (**Table 2** and **Supplementary Results, Figures 4.11–4.15**). Subgroup analysis also suggested that the interventions did not significantly reduce LDL-c in the high-risk population (**Supplementary Results, Figure 5.2**).

#### Impact of Interventions on Blood Pressure

**Figure 3:**
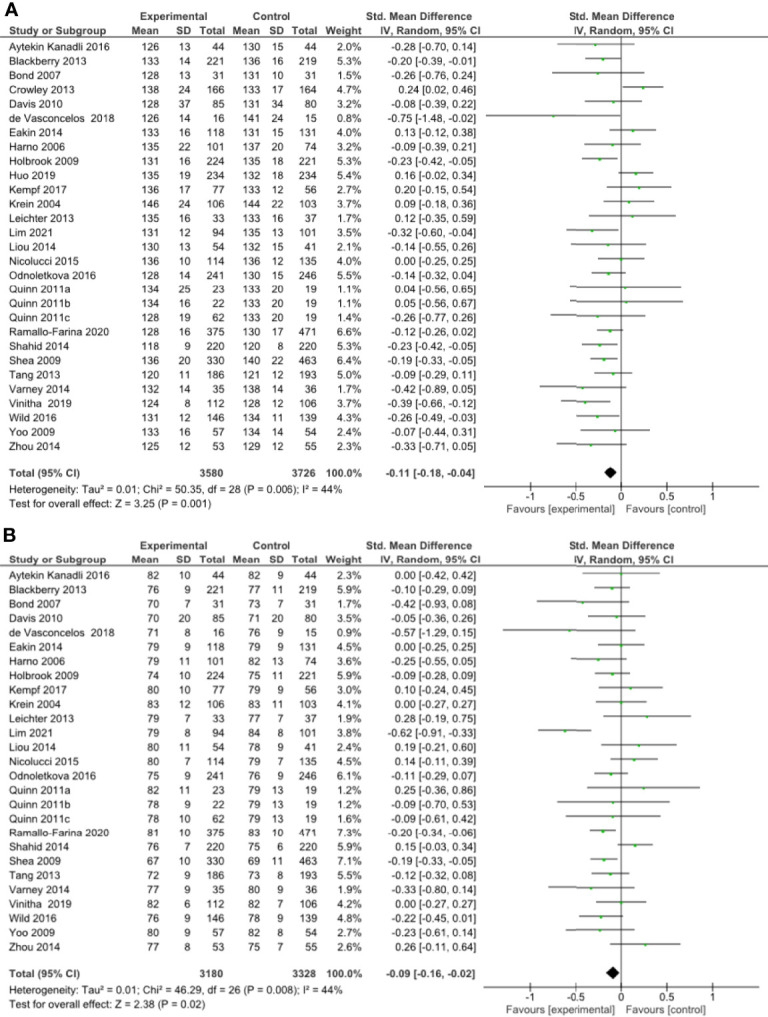
**(A)** Forest plot showing the effect of remote management on systolic blood pressure, **(B)** Forest plot showing the effect of remote risk factor management on diastolic blood pressure.

A meta-analysis of all 27 RCTs incorporating 3580 participants in the intervention group and 3726 participants in the control group found that remote risk factor management significantly reduced SBP compared to usual care (SMD -0.11, 95% CI -0.18 to -0.04, Z=3.25, p=0.001) with a low degree of heterogeneity (I²= 44%) (**Figure 3A**). The funnel plot was asymmetrical (**Supplementary Results, Figure 1.4**). LOO sensitivity analyses suggested removal of any individual RCT did not affect the significance of the main finding (**Supplementary Results, Table 2.4**). Exclusion of studies with high risk of bias changed the significance of the outcome (SMD -0.09, 95% CI -0.18 to 0.00, Z=1.96, p=0.05) with a moderate degree of heterogeneity (I²= 56%) and the funnel plot was asymmetrical (**Supplementary Results, Figures 3.7, 3.8**). Subgroup analyses focused on remote intervention type did not change the significance of the outcome (**Table 2** and **Supplementary Results, Figures 4.16-4.20**). Subgroup analysis suggested that the interventions did not significantly reduce SBP in the high-risk population (**Supplementary Results, Figure 5.3**).

A meta-analysis of all 27 RCTs incorporating 3180 participants in the intervention group and 3328 participants in the control group found that remote risk factor management significantly reduced DBP compared to usual care (SMD -0.09, 95% CI -0.16 to -0.02, Z=2.38, p=0.02) with a low degree of heterogeneity (I²= 44%) (**Figure 3B**). The funnel plot was asymmetrical (**Supplementary Results, Figure 1.5**). LOO sensitivity analyses suggested removal of any individual RCT did not affect the significance of the main finding (**Supplementary Results, Table 2.5**). Exclusion of studies with high risk of bias did not change the significance of the outcome (**Supplementary Results, Figure 3.9**). Subgroup analyses suggested that monitoring of risk factors, coaching of risk factor modification and pharmacological management but not patient education and consultation significantly reduced DBP compared to usual care (**Table 2** and **Supplementary Results, Figures 4.21-4.25**). There were insufficient studies to undertake subgroup analysis of a high-risk population.

### Secondary Outcome Measures

**Supplementary Tables 9 and 10** report the secondary outcome data. One study reported on major adverse cardiovascular events (51), but none of the studies reported on limb revascularization or amputation, or progression of microvascular disease or worsening of existing comorbidities. One study reported that the cost of the intervention was more than the control due to telemonitoring service costs and additional nurse phone consultations (51). None of the studies undertook a cost-benefit analysis. Quality of life data were reported in seven RCTs (28, 34–36, 41, 50, 51), but could not be combined in meta-analysis due to heterogeneity of instruments used. Two studies reported significant improvements in quality of life in the remote management group at follow-up compared to baseline (36, 41). Fifteen RCTs reported on adverse outcomes (28, 30, 32, 34–39, 41, 42, 46, 48, 51, 53). Four RCTs reported on medication related side effects including hypoglycemia and postural hypotension (32, 39, 51, 53). Mortality during follow-up was reported in 17 RCTs (27–32, 34, 35, 37, 39, 41–43, 46–48, 51).

A meta-analysis including 15 RCTs incorporated 2979 participants in the intervention group and 2955 participants in the control group found that remote risk factor management had no effect on overall adverse outcomes (RR = 0.88, 95% CI 0.70 to 1.09, Chi^2 =^ 12.85, p=0.24) with a low degree of heterogeneity (I²= 7%) (**Supplementary Results, Figure 6.1**). The funnel plot was asymmetrical (**Supplementary data, Figure 7.1**). Additional meta-analyses of individual adverse events including mortality, hypoglycemic episodes and hospital admission showed no significant difference between groups (**Supplementary Results, Figures 6.2-6.4**).

## Discussion

This meta-analysis suggested that remote management significantly improved control of the five modifiable risk factors for diabetes-related major adverse events. Small reductions in HbA1c and TC and modest reductions in LDL-c, SBP and DBP were found. The main findings were robust in sensitivity analyses but clarity on which components of the remote management were most effective was limited because all interventions included a composite of different intervention types. Remote risk factor management had no effect on the rate of adverse outcomes including mortality, hypoglycemic episodes and hospital admissions.

The generalizability of the findings of this meta-analysis need to consider the populations studied. These were mainly people with diabetes without a history of major adverse events but with poor risk factor control at entry (56–59). The findings may not be generalizable to populations where risk factors are already well controlled or those with a past history of diabetes-related major adverse events (60). It is also likely that the not all populations are able to engage with remote delivery of healthcare (61, 62). While some RCTs provided participants with mobile phones, computers or internet services or training (43, 44, 51, 52), most did not. There appears to be a separation between those who have access to, and the ability to understand diverse technological resources, and those who do not (‘the digital divide’) (62). For many vulnerable populations such as older persons and those from low socioeconomic, very remote and low educational backgrounds and those with physical disability and/or visual or hearing impairment, remote interventions may not be suitable or readily available. Therefore, factors such as access to the internet of things and electronic devices, user friendliness and ease of navigation of medical technology are important considerations when designing remote interventions. For some populations and certainly for some aspects of medical management, in-person models of care such as home visits are essential and therefore entirely remotely delivered models of care are unsuitable (55).

Subgroup analyses suggested that multiple components of the interventions contributed to the value of the remote management. This included patient education, monitoring of risk factors, coaching, remote consultations and pharmacological management for HbA1c and SBP. The components of patient education for TC and DBP, risk factor coaching for LDL-c, remote consultation for TC and DBP and pharmacological management for TC appeared to be less effective. Conclusions on this are however limited due to the integrated nature of all the interventions studied.

There were several limitations to this meta-analysis. First, an individual-level data analysis was not possible and thus it was not possible to analyse the effect of differing population characteristics and intervention types in detail. The interventions tested were heterogeneous and included multiple components. We sought to examine which components were more effective but since all interventions include more than one component, this analysis was incomplete. An intention to treat analysis was not possible due to loss to follow-up. Most funnel plots suggested a risk of publication bias and thus the effect of the interventions may have been over-estimated. Due to lack of consistent data, we could not perform pooled analyses of planned secondary outcomes including quality of life, cost-effectiveness, micro-vascular outcomes, limb events or MACE. A cost-benefit analysis of using remote intervention compared to standard care remains an important area of future research. Lastly, the adherence to treatments were not reported in most studies and therefore we could not evaluate the impact of adherence on outcome. There were several strengths to our study including carefully planned analyses, the inclusion of RCTs which reported on all three risk factors of interest, extensive evaluation of risk of bias and the reporting of sensitivity and subgroup analyses to evaluate relationships between subgroups and individual studies and pooled outcomes.

## Conclusion

This meta-analysis suggests that remotely managing modifiable risk factors significantly lowers HbA1c, total cholesterol, LDL-cholesterol, and systolic and diastolic blood pressure in people with diabetes. Patient coaching on risk factor management and the provision of pharmacological management were identified as the most effective interventions at improving risk factor control. Further research is needed to rigorously clarify the most effective components of remote management.

## Data Availability Statement

The original contributions presented in the study are included in the article/**Supplementary Material**. Further inquiries can be directed to the corresponding author.

## Author Contributions

MF conceived the research topic, conducted the search strategy, extracted the data, analyzed the data, and wrote the manuscript. LS extracted the data, cleaned the data, and reviewed the manuscript. AD conceived the research topic, conducted the search strategy, extracted the data, cleaned the data, and reviewed the manuscript. BC extracted the data, cleaned the data, and reviewed the manuscript. JG conceived the research topic, supervised the other authors in writing the manuscript, and reviewed the manuscript. JG is the guarantor of this work, and as such had full access to the data in the study and takes responsibility for the integrity of the data and the accuracy of the data analysis. All authors contributed to the article and approved the submitted version.

## Funding

This work was supported by the Townsville Hospital and Health Service Study, Education and Research Trust Account (SERTA) Fund, the James Cook University Strategic Research Investment Fund, and the Queensland Government. JG holds a Practitioner Fellowship from the National Health and Medical Research Council (1117061) and a Senior Clinical Research Fellowship from the Queensland Government, Australia. The funders played no role in study design, conduct, data collection, analysis and interpretation, and did not assist in preparation or review of this manuscript.

## Conflict of Interest

The authors declare that the research was conducted in the absence of any commercial or financial relationships that could be construed as a potential conflict of interest.

## Publisher’s Note

All claims expressed in this article are solely those of the authors and do not necessarily represent those of their affiliated organizations, or those of the publisher, the editors and the reviewers. Any product that may be evaluated in this article, or claim that may be made by its manufacturer, is not guaranteed or endorsed by the publisher.
