# Corrigendum: Effectiveness of Remotely Delivered Interventions to Simultaneously Optimize Management of Hypertension, Hyperglycemia and Dyslipidemia in People With Diabetes: A Systematic Review and Meta-Analysis of Randomized Controlled Trials

**DOI:** 10.3389/fendo.2022.916377

**Published:** 2022-05-09

**Authors:** Malindu E. Fernando, Leonard Seng, Aaron Drovandi, Benjamin J. Crowley, Jonathan Golledge

**Affiliations:** ^1^ Queensland Research Centre for Peripheral Vascular Disease, College of Medicine and Dentistry, James Cook University, Townsville, QLD, Australia; ^2^ Ulcer and Wound Healing Consortium (UHEAL), Australian Institute of Tropical Health and Medicine, James Cook University, Townsville, QLD, Australia; ^3^ Faculty of Health and Medicine, School of Health Sciences, University of Newcastle, Newcastle, NSW, Australia; ^4^ Australian Institute of Tropical Health and Medicine, James Cook University, Townsville, QLD, Australia; ^5^ Department of Vascular and Endovascular Surgery, Townsville University Hospital, Townsville, QLD, Australia

**Keywords:** blood pressure, cholesterol, lipids, systematic review, telehealth

In the original article, there were mistakes in **Figure 2B**, **Figure 2C**, **Figure 3A** and **Figure 3B** as published. The corrected **Figures 2** and **3** appear here.

**Figure 2:**
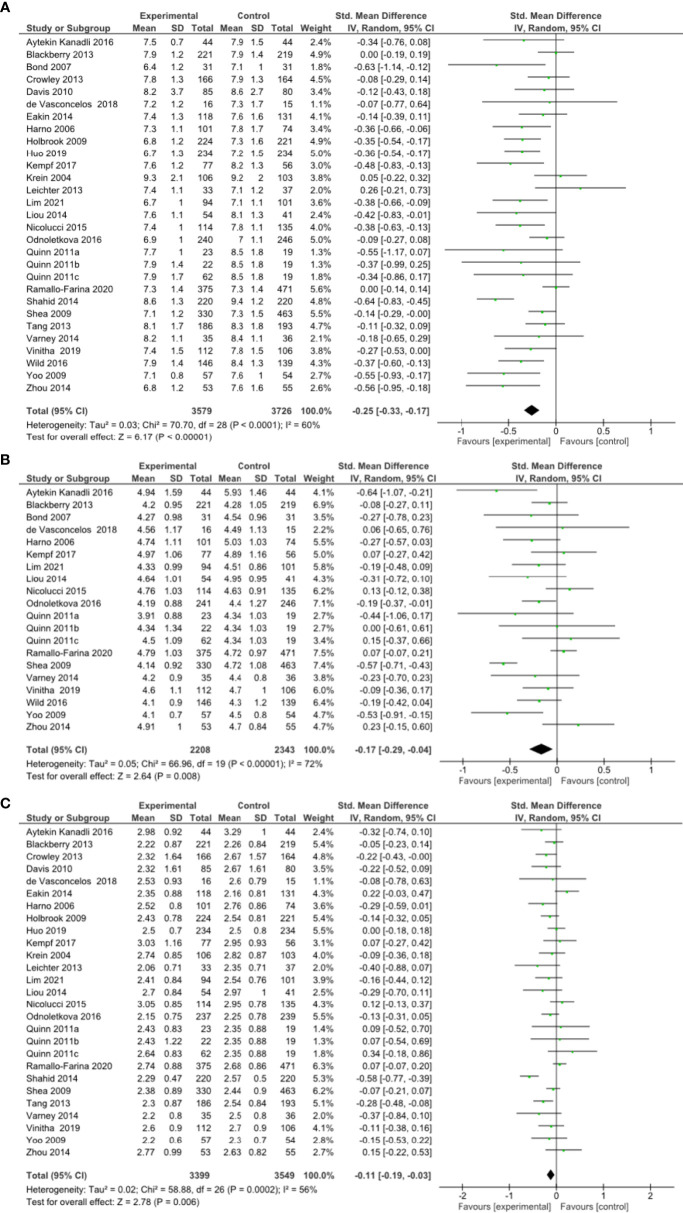
**(A)** Forest plot showing the effect of remote risk factor management on HbA1c, **(B)** Forest plot showing the effect of remote management on total cholesterol, **(C)** Forest plot showing the effect of remote risk factor management on LDL-cholesterol.

**Figure 3:**
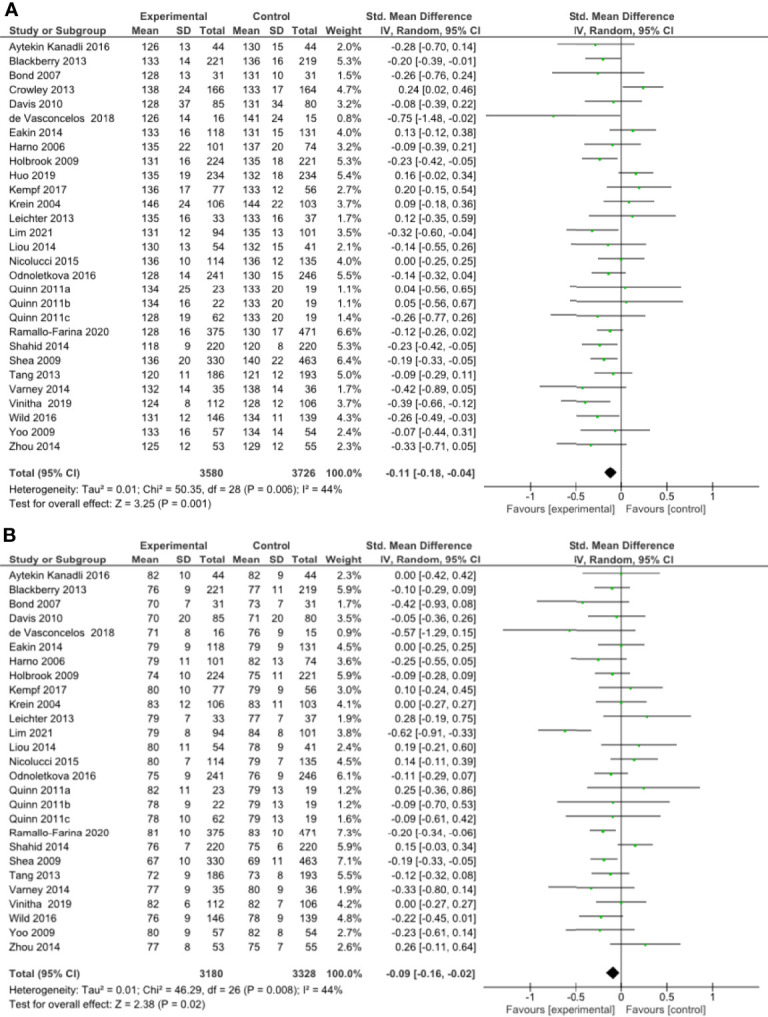
**(A)** Forest plot showing the effect of remote management on systolic blood pressure, **(B)** Forest plot showing the effect of remote risk factor management on diastolic blood pressure.

The authors apologize for this error and state that this does not change the scientific conclusions of the article in any way. The original article has been updated.

## Publisher’s Note

All claims expressed in this article are solely those of the authors and do not necessarily represent those of their affiliated organizations, or those of the publisher, the editors and the reviewers. Any product that may be evaluated in this article, or claim that may be made by its manufacturer, is not guaranteed or endorsed by the publisher.

