# Supplementary material for: Effectiveness of Remotely Delivered Interventions to Simultaneously Optimize Management of Hypertension, Hyperglycemia and Dyslipidemia in People With Diabetes: A Systematic Review and Meta-Analysis of Randomized Controlled Trials
Source: Front Endocrinol (Lausanne). 2022 Mar 15;13:848695. doi: 10.3389/fendo.2022.848695 (PMC8965099; doi:10.3389/fendo.2022.848695)

ONLINE SUPPLEMENTARY FILE

# Supplementary Text 1: Search strategy

Trial[Title] OR random*[Title] OR RCT[Title] AND (online*[Title] OR internet*[Title] OR computer*[Title] OR web[Title] OR web-based*[Title] OR home*[Title] OR at home*[Title] OR ambulatory*[Title] OR continuous*[Title] OR tele*[Title] OR eHealth*[Title] OR e-Health*[Title] OR e Health*[Title] OR electronic health*[Title] OR electronic*[Title] OR mHealth*[Title] OR m-Health*[Title] OR m Health*[Title] OR mobile*[Title] OR mobile health*[Title] OR smartphone*[Title] OR text-messag*[Title] OR text messag*[Title] OR textin* messag*[Title] OR electronic messag*[Title] OR technolog*[Title] OR digital*[Title])) AND (diabet*[Title] OR diabet*[Title] OR glucose*[Title] OR blood sugar*[Title] OR glucose self-monitoring*[Title] OR glucose tolerance test*[Title] OR glycated*[Title] OR glycaemic*[Title] OR glycemic*[Title] OR hyperglycaem*[Title] OR hyperglycem*[Title] OR hypoglyc*[Title] OR metformin*[Title] OR sulfonylurea*[Title] OR insulin*[Title] OR anti-diabet*[Title] OR antidiabet*[Title] OR diabetic neuropathy*[Title] OR peripheral*[Title] OR PVD[Title] OR PAD[Title] OR claudic*[Title] OR ischaemic*[Title] OR ischemia*[Title] OR intermittent*[Title] OR dyslipid*[Title] OR hypercholesterol*[Title] OR high cholesterol*[Title] OR cholesterol*[Title] OR lipid*[Title] OR triglycer*[Title] OR statin*[Title] OR LDL[Title] OR lipoprotein*[Title] OR lipid lowering*[Title] OR ezetimibe*[Title] OR hypertens*[Title] OR blood press*[Title] OR systolic*[Title] OR ambulatory*[Title] OR home based*[Title] OR home-based*[Title] OR anti-hypertens*[Title] OR antihypertens*[Title] OR angiotens*[Title] OR beta-adrenergic*[Title] OR beta-block*[Title] OR beta block*[Title] OR beta adrenergic*[Title] OR calcium-channel*[Title] OR calcium channel*[Title] OR diuret*[Title] OR athero*[Title] OR arteri*[Title] OR antiplate*[Title] OR anti-plate*[Title] OR anti plate*[Title] OR coagula*[Title] OR anti-throm*[Title] OR antithrom*[Title] OR anti throm*[Title] OR thromb*[Title] OR aspirin*[Title] OR clopidogrel*[Title] OR rivaroxaban*[Title] OR smok*[Title] OR tobacco*[Title] OR nicotin*[Title] OR exercis*[Title] OR strenghten*[Title] OR physical activit*[Title] OR physical fitness*[Title] OR diet*[Title] OR lifestyle*[Title] OR self-manag*[Title] OR self manag*[Title] OR prevent*[Title] OR primary prevent*[Title] OR manag*[Title] OR supervis*[Title] OR prescript*[Title] OR monitor*[Title])

# Supplementary Text 2: Data analysis

Numerical data was reported as mean and standard deviation (SD) and categorical data as number and percentage (%). Where 95% confidence intervals (CIs) and not SD were reported, these were converted to SD using the equation SD= √N x (upper limit 95% CI-lower limit 95% CI)/3.92. Where only the standard error (SE) was reported, this was converted to SD by using the formula: SD = SE x √N.^26^ Meta-analyses were performed for any primary or secondary outcome with data extractable from a minimum of three studies. Where there was more than one intervention group receiving remote interventions in an RCT, the intervention groups were analyzed separately in the meta-analysis by splitting the control group and using the reported control group mean and SD.^26^ The meta-analyses were conducted using the inverse-variance method for continuous outcomes and the Mantel-Haenszel statistical method for dichotomous outcomes with random effect models anticipating substantial heterogeneity.^26^ The results were reported as standardized mean difference (SMD)^27^ or risk ratio (RR) and 95% CI for dichotomous outcomes.^26^ SMDs were interpreted as Cohen’s d values^28^, with d=0.2 regarded as a 'small' effect size, d=0.5 as a 'medium' effect size and d=0.8 as a 'large' effect size. All statistical tests were two-sided and a p-value <0.05 was considered significant. Heterogeneity was assessed using I^2^ statistic values (interpreted as 0 to 49%: low, 50 to 74%: moderate and 75 to 100%: high).^29^ Leave-one-out (LOO) sensitivity analyses assessed the contribution of each study by excluding individual studies one at a time and recalculating the remaining pooled estimates. Additional sensitivity analyses were performed excluding studies with high risk of bias.^25^

Five distinct aspects of the remote management programs tested were defined in an attempt to clarify which aspects of the interventions were most important in improving outcome: 1) patient education, 2) monitoring of risk factors, 3) coaching to improve risk factor control, 4) health care professional telehealth consultation and 5) pharmacological management. We only included remote risk factor monitoring RCTs in a meta-analysis where either blood pressures, blood glucose or blood lipids were remotely monitored. Sub-group meta-analyses were performed for any primary outcome with data available from a minimum of three studies per remote intervention component. If any of the component of the intervention was not delivered remotely (i.e., patient education delivered remotely and in person), this study was excluded from meta-analysis.

Subgroup meta-analysis was also planned to evaluate whether remote management was more effective in studies which only included a higher risk population defined as; a documented history of cardiovascular disease, a diabetes duration of greater than 10 years, HbA1c of >10.0% and/or LDL of >2.0 mmol/L and/or systolic blood pressure of >130 mmHg and/or a diastolic blood pressure of >80 mmHg or a previous history of diabetes related complications at entry. Publication bias was assessed by funnel plots comparing the summary estimate of each study and its precision (1/standard error).^30^ All analyses were conducted with Review Manager (RevMan 5) version 5.4 (The Cochrane Collaboration, 2020).

# Supplementary Text 3: Description of the types of intervention tested

A detailed description of the types of interventions is given in **Supplementary Table 5**, with **Supplementary Table 6** providing a summary overview of the key elements of the intervention provided in each trial.

Fourteen RCTs evaluated a form of telephone based remote intervention.^31, 32, 34, 36, 40-42, 45-47, 49, 52, 53, 57^ These ranged from telephone coaching, telephone-based feedback on risk factor control and telephone interventions in combination with other modalities such as web-based online platforms and computer based monitoring systems. Four RCTs evaluated mobile phone text messaging as part of their intervention.^39, 48, 54, 56^ One RCT evaluated an entire secondary prevention program delivered via text messaging.^39^ The intervention was based on risk factor modification and developed by health professionals in combination with guideline-based recommendations. Another RCT evaluated the efficacy of follow-up text-messaging reinforcement of health advice following an initial face-to-face interaction.^54^ Two RCTs used text-message reminders in combination with a web-based portal.^48, 56^ Three RCTs evaluated video-conferencing feedback or education.^35, 44, 50^ These interventions were nurse-led and ranged from individualized or group-based education, increased risk factor monitoring and modification in consultation with treating primary care physicians or endocrinologists.^35, 50^

Nine RCTs used a web-based data monitoring system or online interactive risk factor management system.^33, 37, 38, 43, 44, 48, 51, 55, 56^ These interventions focused on providing participants with online resources including diabetes-related information, online advice and counselling, tailored self-management instructions, weekly discussion forums and risk factor data logging and medication reminders. Some web-based interventions were combined with text-message reminders to participants^37^, individualized risk factor modification and education via videoconference^44^ or phone reminders.^38^ One RCT used a smart-phone application to deliver the entire intervention including regular risk factor assessment, dietitian input, motivational videos and behavioural intervention, individualized goal setting and reminders.^43^ In one RCT, both participant and their health care professionals received online education, electronic online monitoring system and periodic feedback.^48^ Additional supports provided to participants are further described in **Supplementary Table 5.**

**Supplementary Table 5** also describes the method of risk factor monitoring in each RCT. All but four RCTs^37, 38, 44, 49, 51, 57^ reported the method of obtaining biochemistry values including blood lipids and HbA1c measurements. Methods for venepuncture included in home blood sampling by trained professionals, clinic-based blood sampling during routine visits and assessments and used electronic patient medical records to obtain routine biochemistry and hematology data. Blood pressure measurements were also performed in various ways, using various techniques including nurse or physician-initiated measurements during routine visits, in-home measurement study personnel or automatic blood pressure measurements performed by participants. Nine RCTs did not specify how blood pressure measurements were performed.^37, 38, 44, 45, 49, 51, 54, 56, 57^ Wireless data-transmission was used to complement the online web-interface in four RCTs by automatically uploading collected risk factor data (blood glucose, blood pressure).^51, 55, 56^ One RCT used a feedback system integrated within the online data management software to provide participants with positive or negative feedback regarding blood glucose control.^56^

# Supplementary Table 1: Excluded studies and reasons for exclusion (n=19).

|  | | | | | | | | | **INTERVENTION GROUP** | | | | **CONTROL GROUP** | | | | |
| --- | --- | --- | --- | --- | --- | --- | --- | --- | --- | --- | --- | --- | --- | --- | --- | --- | --- |
| **Study** | **Reason for exclusion** | **Country** | **Design** | **Total Number randomized** | **Attrition** | **Duration of follow-up** | **Type of remote intervention tested** | **Population description** | **N** | **Age** | **Females** | **Diabetes duration** | **Control group description** | **N** | **Age** | **Females** | **Diabetes duration** |
| Azizi  [2016] | Incomplete outcome data | Iran | Two-arm parallel group RCT | 72 | 19  (26.4%) | 4-months | Web-based diabetic personal health record | People with type 2 diabetes attending an endocrinology practice | 36 | NR | NR | NR | Usual care | 36 | NR | NR | NR |
| Earle  [2010] | Incomplete outcome data | UK | Two-arm, parallel-group RCT | 137 | 42  (30.7%) | 6-months | Mobile-app supporting clinician blood pressure management | People with diabetes and hypertension | 72 | 59.6  (12.0) | NR | 13.3  (8.6) | Usual care | 65 | 57.1  (13.0) | NR | 11.7  (8.0) |
| Fang  [2018] | Both intervention and control group had remote-monitoring | China | Single-center RCT | 129 | 20  (15.5%) | 12-months | Text-messaging behaviour support | People with type 2 diabetes attending outpatient clinics with established CV-risk factors | 67 | NR | NR | NR | Phone calls only | 62 | NR | NR | NR |
| Fischer  [2012] | Incomplete outcome data | USA | Two-arm, single-center RCT | 762 | NR | 20-months | Nurse-led telephone outreach service | People with diabetes | 381 | 58.5  (12.4) | 244  (64.0%) | NR | Usual care | 381 | 58.3  (12.1) | 218  (57.2%) | NR |
| Frias  [2017] | Incomplete outcome data | USA | Non-blinded, multi-center, parallel-group  cluster-RCT | 69 | 6  (8.7%) | 3-months | Digital medicine and mobile app | People with type 2 diabetes & SBP ≥140 mmHg and HbA1c (≥7%) failing control on ≥2 antihypertensive meds and oral diabetes therapy | 40 | 56.7  (11.4) | 24  (60.0%) | NR | Standard care only | 29 | 61.6  (9.2) | 10  (34.4%) | NR |
| Goodarzi  [2012] | Incomplete outcome data | Iran | Non-blinded, two-arm, parallel-group single-centre RCT | 100 | 19  (19.0%) | 3-months | Text-messaging | People with type 2 diabetes | 50 | NR | NR | NR | NR | 50 | NR | NR | NR |
| Hansen [2017] | Incomplete outcome data | Denmark | 1:1 parallel group RCT | 165 | 26  (15.8%) | 14-months | Video consultations | Patients with poorly regulated type 2 diabetes | 83 | 57.8 (9.4) | 30 (36%) | 12.1  (6.6) | Usual care | 82 | 58.3 (9.3) | 29 (35.0%) | 12.5  (7.3) |
| Jarab [2012] | Incomplete outcome data | Jordan | Parallel group RCT | 171 | 15  (8.8%) | 6-months | Clinical pharmacist-led pharmaceutical care program | Patients with type 2 diabetes | 85 | 63.4 [10.1] | 36 (42.4%) | 9.7  (7.4) | Usual care | 86 | 65.3 [9.2] | 38 (44.2%) | 10.1  (7.7) |
| Kardas  [2016] | Incomplete outcome data | Poland | Two-arm, parallel-group RCT | 62 | 2  (3.2%) | 1.5-months | Smart-phone-based mobile health program | People with type 2 diabetes treated with metformin | 30 | 59.9  (5.3) | 13  (43.0%) | NR | Standard care only | 30 | 59.0  (8.1) | 11  (37.0%) | NR |
| Karhula  [2015] | Incomplete outcome data | Finland | Non-blinded, two-arm, parallel-group RCT | 287 | 62  (21.6%) | 12-months | Mobile-based health coaching program | People with heart disease or type 2 diabetes | 208 | NR | NR | NR | Standard care only | 79 | NR | NR | NR |
| McMahon  [2005] | Incomplete outcome data | USA | Two-arm, single-center RCT | 104 | 25  (24.0%) | 12-months | Web-based management program | People with diabetes and HbA1c >9% | 52 | 64  (7) | 1  (2%) | 12.4  (NR) | Education and usual care | 52 | 63  (7) | 0 | 12.2  (NR) |
| Mons  [2013] | Incomplete outcome data | Germany | Non-blinded, Two-arm, parallel-group, multi-center RCT | 204 | 19  (9.3%) | 18-months | Telephone counselling sessions | People with type 2 diabetes and HbA1c >7.5% | 103 | 68.0  [17] | 41  (39.8%) | 9.0  [7.5] | Usual care | 101 | 67.0  [15] | 38  (37.6%) | 9.0  [10.0] |
| O’Connor  [2014] | Incomplete outcome data | USA | Single-blinded, two-arm, parallel-group, multi-center cluster RCT | 2378 | 276  (11.6%) | Varied | Medication adherence via telephone outreach | People with type 2 diabetes, uncontrolled HbA1c, LDL, or BP, and a new related medication | 1220 | 61.7  (13.0) | 623  (51.1%) | NR | Usual care | 1158 | 62.0  (13.4) | 615  (53.1%) | NR |
| Pressman  [2014] | Incomplete outcome data | USA | Single-blinded, two-arm, Parallel-group RCT | 225 | 27  (12.0%) | 6-months | Telemonitoring device | People with type 2 diabetes and HbA1c between 7.5 and 10% | 118 | 54.8  (9.8) | 40  (37.3%) | NR | Diabetes nurse management | 107 | 56.4  (8.7) | 36  (39.6%) | NR |
| Ralston  [2009] | Incomplete outcome data | USA | Non-blinded, two-arm, parallel-group, single-center RCT | 83 | 9  (10.8%) | 12-months | Web-based care management | People with type 2 diabetes and HbA1c >7% | 42 | 57.0  (NR) | 20  (47.6%) | NR | Usual care | 41 | 57.6  (NR) | 21  (51.2%) | NR |
| Rasmussen  [2016] | Incomplete Outcome data | Denmark | Non-blinded, two-arm, parallel-group, single-center RCT | 40 | 0  (0.0%) | 6-months | Teleconferencing program with video consultations | People with type 2 diabetes aged 40-85 years | 18 | 60.7  [46-83] | 5  (27.8%) | 10.7  [2-21] | Usual care | 22 | 64.6  [36-80] | 8  (36.4%) | 8.4  [0-22] |
| Rodriquez-Idigoars  [2009] | Incomplete outcome data | Spain | Non-blinded, two-arm, parallel-group RCT | 328 | 31  (9.5%) | 12-months | Telemedicine program with phone consultations | People >30 years with type 2 diabetes and were self-monitoring for at least 6-months prior the study | 161 | 63.3  (11.0) | 74  (46.0%) | 11.3  (7.4) | Usual care | 167 | 64.5  (10.2) | 85  (50.9%) | 10.2  (7.3) |
| Soo  [2011] | Incomplete outcome data | South Korea | Multi-Arm RCT | 154 | 10  (6.5%) | 6-months | Individualized health management system | Elderly patients with type 2 diabetes | 51 | 67.2 (4.1) | 27 (52.9%) | 14.1 (10.1) | Usual care | 52 | 68.1 (5.5) | 31 (59.6%) | 15.8 (10.7) |
| Tutino  [2017] | Incomplete outcome data | China | Non-blinded, parallel-group, multi-center RCT | 3586 | 1027  (28.6%) | 12-months | Web-portal and remote monitoring with additional nursing support | People with type 2 diabetes | 1858 | 56.1 (11.6) | 847 (45.6%) | 5.0  [1.0-10.0] | Web-based portal and standard care | 1728 | 56.8 (11.7) | 787  (45.5%) | 5.0  [1.0-10.0] |

Legend: Data are presented as n (%), mean (standard deviation SD), or median [interquartile range] unless otherwise specified.

# Supplementary Table 2: Detailed descriptions of the inclusion and exclusion criteria for each study and the number of participants screened and excluded.

| **Study** | **Risk group based on entry criteria*** | **Inclusion criteria** | **Exclusion criteria** | **Number screened and excluded** |
| --- | --- | --- | --- | --- |
| Aytekin Kanadli [2016] | Unknown risk | People with Type 1 or Type 2 diabetes who were communicative and willing to have regular contact via phone calls. | Hearing, speech, or mental disorders. | Number screened NR; 3 excluded due to failure to contact via telephone. |
| Blackberry [2013] | Unknown risk | Diagnosis of Type 2 diabetes, with most recent HbA1c within past 12 months >7.5%, aged more than 18 years, receiving healthcare from participating general practice and contactable by telephone. | Complex debilitating coexisting medical condition (e.g. severe mental illness or end stage cancer) or were unable to provide signed consent. | 829 screened and 267 excluded. |
| Bond  [2007] | Unknown risk | People with Type 1 or Type 2 diabetes diagnosed at least 12-months ago, aged 60 years or older, living independently, and fluent in oral English. | Moderate or severe cognitive, visual, or physical impairment, or severe comorbid disease (e.g. end-stage renal disease, blindness, terminal cancer). | 67 screened and 5 excluded |
| Crowley  [2013] | Unknown risk | ≥18 years; self-reported black/African American race; ≥1 PCP visit in the past year, a type 2 diabetes International Classification of Diseases, Ninth Revision code (250.x0/250.x2) within 3 years, and ≥1 hemoglobin A1c (HbA1c) measurement in the past year. | Individuals were excluded for diagnosis of dementia, psychosis, or metastatic cancer; receipt of dialysis; recent (3 months) hospitalization for stroke, myocardial infarction, or coronary revascularization; pregnancy, expected pregnancy, or breast-feeding; nursing home residence; lack of telephone access; severely impaired speech/vision; or not speaking English. | 1508 screened and 1146 excluded. |
| Davis  [2010] | Unknown risk | People with Type 1 or Type 2 diabetes aged >35 years with HbA1c >7% seen within a community health center in the last 12 months and able to participate in a 12-month trial. | BMI <25, pregnancy, illness that prevents safe participation. | 869 screened and 704 excluded. |
| de Vasconcelos [2018] | Unknown risk | Patients with Type 2 diabetes for at least one year; are literate; are aged 18 years or over; had a telephone number (landline or mobile); and were able to attend the face-to-face stages of the study. | Patients were excluded if they had limb amputations or reported renal insufficiency, blindness, severe cardiovascular disease or uncontrolled hypertension. | 84 screened and 48 excluded. |
| Eakin  [2014] | Unknown risk | People with Type 2 diabetes aged 20-75 years, physically inactive (<5 days/week of at least 30 minutes planned exercise) and/or overweight or obese (BMI >25). | Not using weight loss medications and without previous or planned bariatric surgery, and no contraindications to unsupervised physical activity. | 1407 screened and 1105 excluded. |
| Harno  [2006] | Unknown risk | NR | NR | 265 screened and 90 excluded. |
| Holbrook  [2009] | Unknown risk | People with Type 2 diabetes aged 18 years or older who were fluent in English and able to understand the study description. | NR | 1610 screened and 1099 excluded. |
| Huo  [2019] | High risk | Adults ≥18 years of age, had documented discharge diagnoses of CHD and DM within the prior 3 years and had access to a mobile phone to read and send text messages. | Patients were excluded if they had cognitive or communication disorders that prevented them from comprehending, detecting, or applying language when attempting to speak or communicate with others. | 3392 screened and 2890 excluded. |
| Kempf  [2017] | Unknown risk | Adults aged between 25 and 79 years of age, had insufﬁcient glycemic control (HbA1c >7.5%), had a BMI > 27 kg/m^2^, and were being treated with at least two different anti-diabetes drugs. | Acute infections, chronic diseases other than Type 2 diabetes and hypertension (e.g., cancer, COPD, asthma, dementia, chronic gut diseases), smoking cessation for <3 months and/or planned smoking cessation, weight-inﬂuencing medication, pregnancy or breast-feeding, intolerance of any ingredient of the PRMR, acute chemotherapy or cortisol treatment. | NR |
| Krein  [2004] | Unknown risk | Those with at least one prescription for an oral hypoglycemic agent, insulin, or blood glucose monitoring supplies filled in the previous 12 months, whose most recent HbA1c was ≥ 8.5% (within the last year) and who had a general medicine clinic visit scheduled between May 1999 and January 2000. | Younger than 18 years, were never diagnosed with diabetes, had Type 1 diabetes or were diagnosed before the age of 30 years, had no telephone, did not speak English, were not competent for interview, reported primary source of diabetes care outside the VA, were being treated for cancer (other than non-melanoma skin cancer), had kidney failure, symptomatic heart failure, liver disease or blindness, spent winter at another residence, or planned to move. | 691 screened and 445 excluded. |
| Leichter  [2013] | Unknown risk | Adults aged 19-65 years with type 1 or type 2 diabetes who were computer literate and able to use self-monitoring blood glucose equipment. | Creatinine clearance <30mL/min, severe diabetic retinopathy, gastroparesis, CVD, hepatic disease, severe neuropathy, recreational drug or significant alcohol use, use of medications that affect blood pressure or blood glucose, pregnant. | NR |
| Lim  [2021] | Unknown risk | Presence of physician-diagnosed type 2 diabetes, aged between 21 to 75 years, BMI 23.0 or greater, literacy in English and smartphone access. | Participants with heart failure, advanced kidney disease, type 2 diabetes, severe cognitive or psychological disabilities, depression, untreated hypothyroidism, thalassemia or blood disorders or who were pregnant were excluded from the study. | 252 screened and 48 excluded. |
| Liou  [2014] | Unknown risk | Type 2 diabetes with HbA1c >7% for at least 12 months. | Pregnant, or had acute or chronic illnesses that prevented safe participation. | NR |
| Nicolucci  [2015] | High risk | Adults aged over 45 years with Type 2 diabetes with HbA1c between 7.5% and 10%, being treated with sulfonylureas or with insulin alone or in combination, and BP >130/80mmHg. | Diabetes being treated with lifestyle interventions or monotherapy with other agents, using multiple insulins, mental conditions preventing understanding or participation, pregnancy, major cardiovascular events within the last 6 months, and any serious health condition that would significantly reduce life expectancy. | NR |
| Odnoletkova  [2016] | Unknown risk | People with Type 2 diabetes aged 18-75 years, and receiving oral or injectable therapy for diabetes. | Corticoid therapy, debilitating comorbidity (e.g. dialysis, mental illness or cancer), residence in a long-term care facility, pregnancy, or unable to speak Dutch. | 684 screened and 110 excluded. |
| Quinn  [2011] | Unknown risk | For general practices: had at least three physicians without academic affiliation who provided diabetes care to at least 10% of their patients and were identified from a list of primary care practices in the study geographic areas. For patients: Physician diagnosis of type 2 diabetes for ≥ 6 months; glycated hemoglobin ≥ 7.5% within 3 months; age 18-64 years. | Patients excluded if they Medicare or Medicaid beneficiaries, uninsured, insulin pump users, not currently managed by study physicians, pregnant, active substance, alcohol or drug abuser (sober < 1 year), psychotic or schizophrenic under active care, severe hearing or visual impairment or no internet or email access. | 2602 screened and 2389 excluded. |
| Ramallo-Farina [2020] | Unknown risk | Patients with type 2 diabetes mellitus diagnosed at least 1 year before study enrollment, aged between 18 and 65 years, formal consent to participate in the study and regular usage of mobile phone. | CKD ≥ stage 3b, urinary albumin to creatinine ratio ≥ 300mg/g or urinary protein excretion ≥ 300mg/24 hours; Acute coronary syndrome (documented angina or myocardial infarction) or stroke in the last 6 months or class III or IV heart failure; proliferative diabetic retinopathy or clinically significant diabetic macular edema requiring previous treatment with retinal photocoagulation, vitrectomy or intravitreal injections of antivascular endothelial growth factor or triamcinolone acetonide 6 months before study inclusion; uncorrected severe hearing or visual impairment or corrected visual acuity ≤ 20/40 by any cause; diabetic foot with ulcers ≥ 2 according to the Wagner scale; Liver cirrhosis; Cancer, unless disease free 5 years after diagnosis; Other terminal illnesses; Intellectual retardation, dementia, and psychotic diseases; Active substance abuse, alcohol, or drugs (must be sober for 1 year); pregnancy; Insufficient (Spanish) language skills; Physical disability limiting participation in group education activities; Concurrent participation in another clinical trial or any other investigational study. | 6402 screened and 4068 excluded. |
| Shahid  [2014] | Unknown risk | 18-70 years of age, residing in rural areas of Pakistan, willing to participate in the study, Type 2 diabetes mellitus ≥ 3 months, ability to understand Urdu and HbA1c ≥8.0% presenting to the outpatient services of Department of Endocrinology, Liaquat National Hospital (LNH). | Patients with complications of DM like diabetic nephropathy, diabetic foot, status post-amputation of limb, diabetic retinopathy, psychiatric illness, pregnant females, patients using insulin, patients who do not have mobile phone facilities and those having other chronic illnesses were excluded from the study. | NR |
| Shea  [2009] | Unknown risk | Adults aged 55 or older, current Medicare beneficiary, has diabetes as defined by a physician’s diagnosis and being treated with diet, oral hypoglycemic agents or insulin, and with residence in a federally designated medically underserved area. | Moderate or severe cognitive impairment, severe visual, mobility or motor coordination impairment, severe comorbid condition, severe expressive or receptive communication impairment, lack of free electrical outlet for home telemedicine unit and spending >3 months a year at a location different from New York State residence. | 2201 screened and 235 excluded. |
| Tang  [2013] | Unknown risk | Age ≥ 18 years, diagnosis of Type 2 diabetes mellitus, HbA1c ≥7.5%, patient seen within past 12 months. | Initial diagnosis of diabetes was within the last 12 months, had an inability to speak or read English, lack of regular internet access with email capabilities, unwillingness to perform any self-monitoring at home (including blood glucose), a diagnosis of a terminal illness and/or entry into hospice care, pregnancy, planning a pregnancy or currently lactating, current enrollment in a care management program at PAMF or elsewhere, family household member enrolled in EMPOWER-D study, resident of a long-term care facility, plans to discontinue primary care at PAMF during the study period or uninsured. | 1594 screened and 1179 excluded. |
| Varney  [2014] | Unknown risk | Adults with Type 2 diabetes and HbA1c >7%. | Patients who were unable to provide informed consent, non-English speaking, cognitively impaired, receiving palliative care, severely hearing impaired or without telephone access were excluded. | 905 screened and 811 excluded. |
| Vinitha  [2019] | Unknown risk | Persons with newly diagnosed Type 2 diabetes and treatment naive, aged 20–60 years of both sexes with values of glycosylated hemoglobin (HbA1c) ≥6.5% (48 mmol/mol) and were able to read and understand the messages were selected. | Persons having Type 1 diabetes, any major illness such as cardiovascular disease, chronic liver or kidney disease, cognitive impairment or other mental imbalance, physical disability, not able to read and understand SMS, pregnant and lactating mothers were excluded from the study. | 261screened and 13 excluded. |
| Wild  [2016] | Unknown risk | Diagnosis of Type 2 diabetes managed in family practice, age over 17 years, availability of a mobile telephone signal at home, and poor glycemic control, defined as HbA1c > 58 mmol/mol (7.5%). | Blood pressure >210/135 mmHg, hypertension or renal disease managed in secondary care, treatment for a cardiac event or other life-threatening illness within the previous 6 months, major surgery within the last 3 months, atrial fibrillation unless successfully treated or cardioverted, inability to use self-monitoring equipment, and pregnancy. | 500 screened and 179 excluded. |
| Yoo  [2009] | High risk | Diagnosis of both Type 2 diabetes and hypertension at least 1 year previously by a physician, HbA1c 6.5 – 10%; blood pressure > 130/80 mmHg, BMI ≥ 23.0kg/m^2^ (overweight according to Asia-Pacific criteria). | Severe diabetic complications (e.g. diabetic foot or severe diabetic retinopathy); liver dysfunction with aspartate aminotransferase or alanine aminotransferase > 2.5 times the reference level, or renal dysfunction (serum creatinine > 132 µmol/l); medical history of congestive heart failure, angina pectoris, myocardial infarction, or stroke based on physician’s diagnosis; pregnancy or lactation; or other medical problems that could affect study results or trial participation. | NR |
| Zhou  [2014] | Unknown risk | Patients diagnosed with Type 2 diabetes according to WHO criteria in 1999 and referred to the First Affiliated Hospital of Jinan University from July 2012 to February 2013. Patients also had to be aged between 18 to 75 years with the ability to manage their glycemic meter at home. | Patients without an internet device as required for the home telemedicine system were excluded. | NR |

**Legend:** BMI= body mass index; HbA1c= glycated hemoglobin; PCP= primary care physician; WHO= World Health Organization. *The risk group was assigned for purposes of meta-analysis and was based on the following: a documented history of cardiovascular disease, a diabetes duration of greater than 10 years, HbA1c of > 10.0% and/or LDL of >2.0 mmol/L and/or systolic blood pressure of > 130 mmHg and/or a diastolic blood pressure of >80 mmHg or a previous history of diabetes related complications at entry.

# Supplementary Table 3: Prescribed medications in intervention and control groups at baseline and follow-up.

| **Study** | **BASELINE** | | | | | | | | **FOLLOW-UP** | | | | | | | |
| --- | --- | --- | --- | --- | --- | --- | --- | --- | --- | --- | --- | --- | --- | --- | --- | --- |
|  | **INTERVENTION GROUP** | | | | **CONTROL GROUP** | | | | **INTERVENTION GROUP** | | | | **CONTROL GROUP** | | | |
|  | **Insulin** | **Statin** | **Anti-platelet** | **ACEi/**  **ARB** | **Insulin** | **Statin** | **Anti-platelet** | **ACEi/**  **ARB** | **Insulin** | **Statin** | **Anti-platelet** | **ACEi/**  **ARB** | **Insulin** | **Statin** | **Anti-platelet** | **ACEi/**  **ARB** |
| Aytekin Kanadli  [2016] | 12  (27.2%) | NR | NR | NR | 11  (25.0%) | NR | NR | NR | NR | NR | NR | NR | NR | NR | NR | NR |
| Blackberry  [2013] | 54  (23.0%) | NR | NR | NR | 56  (24.0%) | NR | NR | NR | 88  (40.0%) | NR | NR | NR | 78  (36.0%) | NR | NR | NR |
| Bond  [2007] | NR | NR | NR | NR | NR | NR | NR | NR | NR | NR | NR | NR | NR | NR | NR | NR |
| Crowley  [2013] | 93  (51.0%) | NR | NR | NR | 92  (52.0%) | NR | NR | NR | NR | NR | NR | NR | NR | NR | NR | NR |
| Davis  [2010] | 41  (48.8%) | NR | NR | NR | 42  (51.9%) | NR | NR | NR | NR | NR | NR | NR | NR | NR | NR | NR |
| de Vasconcelos  [2018] | NR | NR | NR | NR | NR | NR | NR | NR | NR | NR | NR | NR | NR | NR | NR | NR |
| Eakin  [2014] | 23  (15.2%) | NR | NR | NR | 20  (13.2%) | NR | NR | NR | 35  (23.5%) | NR | NR | NR | 36  (23.9%) | NR | NR | NR |
| Harno  [2006]* | NR | NR | NR | NR | NR | NR | NR | NR | NR | NR | NR | NR | NR | NR | NR | NR |
| Holbrook  [2009] | 39  (15.4%) | NR | NR | NR | 47  (18.2%) | NR | NR | NR | NR | NR | NR | NR | NR | NR | NR | NR |
| Huo  [2019] | 16  (6.4%) | 147  (58.6%) | 154  (61.4%) | 70  (27.9%) | 18  (7.2%) | 146  (58.2%) | 153  (61.0%) | 76  (30.3%) | 27  (10.8%) | 211  (84.1%) | 217  (86.5%) | 118  (47.0%) | 35  (13.9%) | 219  (87.3%) | 220  (87.7%) | 107  (42.6%) |
| Kempf  [2017] | 62  (67.0%) | NR | NR | NR | 56  (76.0%) | NR | NR | NR | 54  (58.0%) | NR | NR | NR | 54  (73.0%) | NR | NR | NR |
| Krein  [2004] | 57  (46.0%) | 35  (28.0%) | 77  (63.0%) | NR | 61  (50.0%) | 29  (24.0%) | 68  (55.0%) | NR | 60  (55.0%) | 51  (46.0%) | 75  (71.0%) | NR | 63  (53.0%) | 40  (38.0%) | 64  (62.0%) | NR |
| Leichter  [2013] | 32  (65.3%) | NR | NR | NR | 32  (65.3%) | NR | NR | NR | NR | NR | NR | NR | NR | NR | NR | NR |
| Liou  [2014] | NR | NR | NR | NR | NR | NR | NR | NR | NR | NR | NR | NR | NR | NR | NR | NR |
| Nicolucci  [2015] | 12  (7.8%) | 85  (55.3%) | NR | 105  (68.4%) | 14  (9.4%) | 76  (51.1%) | NR | 98  (65.9%) | 8  (7.0%) | 64  (56.1%) | NR | 78  (68.5%) | 15  (11.1%) | 75  (55.6%) | NR | 93  (69.2%) |
| Odnoletkova  [2016] | NR | 197  (68.6%) | 138  (48.1%) | NR | NR | 209  (72.8%) | 136  (47.4%) | NR | NR | NR | NR | NR | NR | NR | NR | NR |
| Quinn  [2011] | NR | NR | NR | NR | NR | NR | NR | NR | NR | NR | NR | NR | NR | NR | NR | NR |
| Shahid  [2014] | 0  (0.0%) | NR | NR | NR | 0  (0.0%) | NR | NR | NR | NR | NR | NR | NR | NR | NR | NR | NR |
| Shea  [2009] | NR | NR | NR | NR | NR | NR | NR | NR | NR | NR | NR | NR | NR | NR | NR | NR |
| Tang  [2013] | NR | NR | NR | NR | NR | NR | NR | NR | NR | NR | NR | NR | NR | NR | NR | NR |
| Varney  [2014] | 25  (53.0%) | NR | NR | NR | 29  (62.0%) | NR | NR | NR | NR | NR | NR | NR | NR | NR | NR | NR |
| Vinitha  [2019] | NR | NR | NR | NR | NR | NR | NR | NR | NR | NR | NR | NR | NR | NR | NR | NR |
| Wild  [2016] | 26  (16.3%) | NR | NR | NR | 25  (15.5%) | NR | NR | NR | 34  (54.4%) | NR | NR | NR | 31  (49.9%) | NR | NR | NR |
| Yoo  [2009] | NR | NR | NR | NR | NR | NR | NR | NR | NR | NR | NR | NR | NR | NR | NR | NR |
| Zhou  [2014] | NR | NR | NR | NR | NR | NR | NR | NR | NR | NR | NR | NR | NR | NR | NR | NR |
| Lim  [2021] | 0  (0.0%) | NR | NR | NR | 0  (0.0%) | NR | NR | NR | NR | NR | NR | NR | NR | NR | NR | NR |
| Ramallo-Farina  [2020] | NR | NR | NR | NR | NR | NR | NR | NR | NR | NR | NR | NR | NR | NR | NR | NR |

**Legend:** ACEi= angiotensin converting enzyme inhibitor, ARB= angiotensin 2 receptor blocker. *Harno et al. 2006 did not report the absolute number of people taking different medication classes, but reported that use of aspirin, statins and ACEi did not differ between the groups at baseline or follow-up.

# Supplementary Table 4: Baseline risk factors between the intervention and control groups.

| **Study** | **Number Randomized:**  **Number Completed** | **Intervention group**  **(n=)** | **Control group**  **(n=)** | **Baseline HbA1c (%)** | | **Baseline systolic blood pressure (mmHg)** | | **Baseline diastolic blood pressure (mmHg)** | | **Baseline LDL-C**  **(mmol/L)** | | **Baseline TC (mmol/L)** | |
| --- | --- | --- | --- | --- | --- | --- | --- | --- | --- | --- | --- | --- | --- |
|  |  |  |  | **Intervention** | **Control** | **Intervention** | **Control** | **Intervention** | **Control** | **Intervention** | **Control** | **Intervention** | **Control** |
| Aytekin Kanadli  [2016] | 91:88 | 47:44 | 44:44 | 7.6  (0.6) | 7.7  (1.5) | 130.0  (16.4) | 127.7  (15.8) | 81.5 (10.3) | 80.4  (9.8) | 3.11  (1.04) | 3.24  (0.95) | 5.03  (1.45) | 5.57  (1.47) |
| Blackberry  [2013] | 473:440 | NR:221 | NR:219 | 7.98  (1.22) | 8.13  (1.34) | 139  (18) | 138  (18) | 79  (10) | 79  (11) | 2.40  (0.89) | 2.40  (0.85) | 4.51  (1.04) | 4.50  (1.20) |
| Bond  [2007] | 62:62 | 31:31 | 31:31 | 7.0  (1.1) | 7.11  (0.91) | 134  (15.0) | 128  (13.2) | 76  (7.7) | 73  (7.1) | NR | NR | 4.40  (1.14) | 4.58  (0.78) |
| Crowley  [2013] | 359:330 | 182:166 | 177:164 | 8.0  (1.35) | 8.0  (1.33) | 138.0  (21.7) | 136.8  (19.2) | NR | NR | 2.58  (1.01) | 2.55  (0.95) | NR | NR |
| Davis  [2010] | 165:165 | 85:85 | 80:80 | 9.3  (1.9) | 8.9  (1.8) | 135.3  (21.2) | 138.5  (19.9) | 76.2  (12.0) | 74.8  (10.4) | 2.81  (0.94) | 2.77  (0.86) | NR | NR |
| de Vasconcelos  [2018] | 36:31 | 18:16 | 18:15 | 8.0  (2.14) | 6.9  (1.31) | 130.3  (19.5) | 137.2  (22.7) | 72.1  (8.2) | 77.2  (10.0) | 2.51  (1.16) | 2.80  (0.91) | 4.40  (1.49) | 4.77  (1.24) |
| Eakin  [2014] | 302:249 | 151:118 | 151:131 | 7.4  (1.54) | 7.3  (1.73) | 134.2  (15.0) | 132.0  (13.8) | 81.9  (9.7) | 81.2  (8.0) | 2.62  (1.02) | 2.47  (0.89) | NR | NR |
| Harno  [2006] | 175:NR | 101:NR | 74:NR | 7.8  (1.3) | 8.2  (1.6) | 134.0  (18.1) | 136.0  (15.5) | 81.0  (10.0) | 84.0  (9.5) | 2.70  (0.80) | 2.65  (0.77) | 4.95  (0.10) | 4.91  (0.86) |
| Holbrook  [2009] | 511:445 | 253:224 | 258:221 | 7.0  (1.4) | 7.1  (1.6) | 135.2  (17.6) | 134.8  (18.4) | 76.1  (11.1) | 74.7  (10.3) | 2.41  (0.65) | 2.59  (0.87) | NR | NR |
| Huo  [2019] | 502:468 | 251:234 | 251:234 | 6.9  (1.4) | 7.1  (1.4) | 135.9  (18.4) | 135.9  (18.1) | NR | NR | 2.6  (0.8) | 2.6  (0.8) | NR | NR |
| Kempf  [2017] | 202:133 | 102:77 | 100:56 | 8.4  (1.3) | 8.2  (1.2) | 139.0  (16.0) | 134.0  (13.0) | 93.0  (10.0) | 81.0  (9.0) | 2.97  (1.03) | 3.03  (0.93) | 5.04  (1.16) | 5.02  (1.24) |
| Krein  [2004] | 246:209 | 123:NR | 123:NR | 9.3  (1.5) | 9.2  (1.4) | 145  (21) | 145  (20) | 86  (12) | 86  (11) | 3.18  (0.96) | 3.18  (0.98) | NR | NR |
| Leichter  [2013] | 98:70 | 49:33 | 49:37 | 7.7  (1.5) | 7.3  (1.2) | 133.2  (14.1) | 132.4  (17.3) | 79.3  (6.1) | 76.9  (9.7) | 2.40  (0.84) | 2.57  (1.60) | NR | NR |
| Lim  [2021] | 204:195 | 99:94 | 105:101 | 7.4  (1.2) | 7.5  (1.3) | 134.7  (13.5) | 135.3  (13.0) | 82.7  (8.8) | 85.7  (9.8) | 2.67  (0.84) | 2.74  (0.9) | 4.62  (0.93) | 4.74  (1.06) |
| Liou  [2014] | 95:95 | 54:54 | 41:41 | 8.3  (1.2) | 8.1  (1.2) | 128.5  (15.2) | 125.7  (13.7) | 79.0 (11.1) | 77.3  (11.8) | 2.87  (0.86) | 3.33  (0.88) | 4.86  (1.10) | 5.46  (0.92) |
| Nicolucci  [2015] | 302:249 | 153:114 | 149:135 | 7.94  (0.8) | 7.99  (0.8) | 142.4  (7.3) | 143.2  (9.7) | 86.6  (3.7) | 87.2  (4.8) | 3.23  (0.91) | 3.10  (0.85) | 4.86  (1.03) | 4.73  (0.98) |
| Odnoletkova  [2016] | 574:474^a^ | 287:228^a^ | 287:246^a^ | 7.0  (1.1) | 7.0  (1.0) | 133.0  (18.0) | 132.0  (17.0) | 75.0  (10.0) | 76.0  (10.0) | 2.40  (0.80) | 2.51  (0.83) | 4.47  (0.96) | 4.60  (1.01) |
| Quinn  [2011] | 213:163 | NR:23^b^ | NR:56 | 9.3 (1.8) | 9.2  (1.7) | 130.0 (18.0) | 130  (22.0) | 79.0 (11.0) | 78  (12) | 2.66 (0.75) | 2.64  (0.93) | 4.68 (0.91) | 4.71  (1.32) |
|  |  | NR:22^c^ |  | 9.0 (1.8) |  | 133.0 (14.0) |  | 79.0 (9.0) |  | 2.66 (0.85) |  | 4.58 (1.09) |  |
|  |  | NR:62^d^ |  | 9.9 (2.1) |  | 130.0 (14.0) |  | 79.0 (9.0) |  | 2.74 (0.85) |  | 4.76 (1.06) |  |
| Ramallo-Farina [2020]^e^ | 1123:846 | 537:375 | 586:471 | 7.3  (1.5) | 7.3  (1.5) | 132.8  (18.1) | 132.6  (18.3) | 84.2  (10.3) | 83.8  (10.2) | 2.82  (0.87) | 2.80  (0.94) | 4.90  (1.0) | 4.83  (1.02) |
| Shahid  [2014] | 440:440 | 220:220 | 220:220 | 10.09 (1.71) | 9.85 (1.37) | 120.8 (10.1) | 121.09 (8.99) | 81.15 (7.37) | 74.54 (5.91) | 2.89 (0.81) | 2.80 (0.56) | NR | NR |
| Shea  [2009] | 1665:793 | 844:330 | 821:463 | 7.36  (1.48) | 7.40  (1.60) | 142.8  (24.2) | 142.5  (23.6) | 71.6  (11.4) | 71.0  (10.4) | 2.76  (0.09) | 2.80  (0.92) | 4.73  (0.96) | 4.78  (1.00) |
| Tang  [2013] | 415:379 | 202:186 | 213:193 | 9.24  (1.59) | 9.28  (1.74) | 126.1  (12.5) | 127.0  (14.4) | 72.7  (9.5) | 72.6  (9.4) | 2.54  (0.88) | 2.46  (0.9) | NR | NR |
| Varney  (2014) | 94:71 | 47:35 | 47:36 | 8.2  (2.6) | 8.5  (1.2) | 140.0  (16.6) | 134.0  (18.4) | 80.0  (12.1) | 76.0  (10.7) | 2.3  (0.6) | 2.4  (0.9) | 4.1  (0.8) | 4.5  (1.2) |
| Vinitha  [2019] | 248:218 | 126:112 | 122:106 | 9.5  (2.1) | 9.5  (1.9) | 129.5  (13.2) | 131.7  (15.0) | 83.2  (8.9) | 83.8  (9.8) | 2.8  (0.9) | 2.9  (0.9) | 4.8  (1.1) | 4.8  (0.9) |
| Wild  [2016] | 321:285 | 160:146 | 161:139 | 8.9  (1.3) | 8.8  (1.1) | 134  (11.9) | 134.9  (11.6) | 78.9  (8.8) | 78.6  (8.7) | NR | NR | 4.2  (1.0) | 4.3  (1.0) |
| Yoo  [2009] | 123:111 | 62:57 | 61:54 | 7.6  (0.9) | 7.4  (0.9) | 140.0  (18.0) | 138.0  (18.0) | 84.0  (10.0) | 83.0  (10.0) | 2.6  (0.7) | 2.4  (0.7) | 4.6  (0.8) | 4.5  (0.9) |
| Zhou  [2014] | 114:108 | 57:53 | 57:55 | 8.44  (1.58) | 8.22  (1.58) | 128.6  (14.3) | 131.9  (12.3) | 79.4  (9.5) | 76.9  (8.1) | 2.75  (0.93) | 2.89  (1.13) | 4.99  (1.21) | 5.23  (1.31) |

**Legend:** Data are presented as raw numbers, or mean (standard deviation) unless otherwise specified. NR = not reported.

^a^ For Odnoletkova et al. (2016) the number of participants for whom outcome data was reported in the two groups differed; HbA1c 240 vs 246 participants, LDL-c 237 vs 239 participants and SBP/DBP 241 vs 247 participants.

Quinn et al. (2011) had three intervention groups, all of which are reported: ^b^ online coaching only, ^c^ coaching and primary care provider portal, and ^d^ coach primary care provider portal with decision-support.

^e^ Ramello-Farina et al. (2020) had several interventional groups and only data from the ‘patient intervention’ group were included.

# Supplementary Table 5: Detailed descriptions of study definitions and primary and secondary outcomes as described in each included trial.

| **Study** | **Description of intervention** | **Description of control** | **Primary and secondary outcomes** | **Method of outcome measurement** | **Additional supports provided** | **Measures of adherence and adverse outcomes** |
| --- | --- | --- | --- | --- | --- | --- |
| Aytekin Kanadli  [2016] | Initial group-based education session followed by weekly phone calls for one month then fortnightly calls for two months. The education session ran for 20-25 minutes in groups of 8-10 participants, and included a diabetes patient education booklet, a visual presentation, and question and answer session. Phone calls involved review of metabolic control, and continued education focused on shortcomings in patient’s care. | Routine treatment and care | NR | **Blood pressure:** Average of two measurements two minutes apart were obtained with patients in a sitting position after resting for at least 10 minutes with right arm supported at heart level.  **HbA1c and lipids:** obtained from patient files. | NR | NR |
| Blackberry [2013] | Usual general practice care + 5 telephone coaching sessions by practice nurses at 6-weekly intervals for first 6 months, 1x telephone coaching session at 8 and 10 months, 1x face-to-face coaching session at 12months and 1x telephone coaching session at 15 months. Nurses discuss progress, empower patients, educate and negotiate plans to attain targets and titrate medication treatments during these sessions. | Usual general practice care which may have included referral to diabetes educators, dietitians and diabetes specialists as part of the standard diabetes care provided by that practice. | **Primary:** HbA1c at 18 months post baseline.  **Secondary:** Lipid profile, renal function, blood pressure, BMI, waist circumference, smoking status, quality of life, diabetes self-efficacy, diabetes support, depression status and intensification of treatment. | **Blood pressure:** Measured at GP practice at baseline, 12 months and 18 months.  **HbA1c and lipids:** Measured at usual local pathology laboratory. HbA1c assay methods used by lab are DCCT aligned and quality assured. | Patients able to contact practice nurses in between coaching sessions if required. Practice nurses had support from research team via monthly telephone calls, a group meeting and a visit to the practice. | NR |
| Bond  [2007] | Access to a website with various resources including diabetes-related information, receiving online advice and counseling from a nurse, tailored self-management instructions, weekly discussion forums, and risk factor data logging. | Standard diabetes care from usual provider including access to education through traditional methods. | **Primary:** HbA1c, weight, blood pressure, HDL, total cholesterol  **Secondary:** NR | **Blood pressure:** Done in participants’ homes by a trained research assistant or phlebotomist using a blood pressure device and calibrated scale.  **HbA1c and lipids:** Done in participants’ homes by a trained research assistant or phlebotomist; single-use HbA1c testing kit and Cholestech LDX analyzer for cholesterol readings. | Instructions on diet, exercise, dealing with physical and emotional demands of diabetes. Intervention participants received computer and internet access and relevant training if needed. | NR |
| Crowley [2013] | Intervention group received monthly self-management support from nurses via telephone calls and quarterly medication management facilitation via nurse-primary care provider communication. | Usual care but this was not defined. Participants received educational material about CVD reduction at baseline. | **Primary:** Change in SBP, HbA1c and LDL from baseline to 12 months.  **Secondary:** Self-reported medication adherence. | **Blood pressure, HbA1c, and lipids:** Collected via review of electronic medical records | All research staff underwent interactive training focusing on cultural sensitivity and awareness of issues facing African Americans. 2 nurse interventionists received intensive training in motivational interviewing. | Adherence was self-reported and based on the Morisky Self-reported Medication-Taking Scale |
| Davis  [2010] | Diabetes self-management education intervention through videoconferencing and in-person sessions. Group and individualised goal setting was used, with participants completing logs of self-monitored risk factors. Clinical guidelines were used to inform on the interventions. | Usual care including access to existing services including care managers and nurse practitioners, in addition to one individual 20-minute diabetes education session using ADA materials at randomisation by a licensed nurse. | **Primary:** HbA1c.  **Secondary:** LDL, blood pressure, and albumin-creatinine ratio. | **Blood pressure:** Average of second and third readings using an Omron HEM-907 IntelliSense device.  **HbA1c and lipids:** determined using Olympus AU400 via immunoassay / absorption spectroscopy. | Intervention participants were offered optional retinal imaging by a nurse under the supervision of an ophthalmologist. They also received a pedometer to track steps. | NR |
| De Vasconcelos [2018] | Intervention group received usual care and telecoaching for 24 weeks. Telecoaching was a programme of guidance/coaching on diabetes and conducted via telephone calls made by a research nurse. The telephone calls were 2x weekly and provided guidance, motivated participants and provided encouragement to adhere to the therapeutic regimen. | Usual nursing care | NR | **Blood pressure:** Nurses did blood pressure measurements during face-to-face contact every 3 months.  **HbA1c and lipids:** High-performance liquid chromatography was used for HbA1c and enzymatic colorimetric was used for lipid measurements. | NR | NR |
| Eakin  [2014] | Telephone-delivered weight loss and physical activity intervention. Participants received a detailed workbook and up to 27 calls over 18 months and used motivational interviewing and emphasized behaviour change. This included targets, goal setting and self-monitoring. | Usual care including being mailed a brief summary of their results after each assessment and given standard diabetes self-management education brochures. | **Primary:** Weight, physical activity, HbA1c.  **Secondary:** Dietary energy intake, diet quality, waist circumference, fasting lipid levels, and blood pressure. | **Blood pressure:** Home visits by a nurse measured in duplicate in a seated position.  **HbA1c and lipids:** Whole blood samples obtained by nurses in the morning after an overnight fast. HbA1c measured using HPLC and lipids by enzymatic colorimetric assay. | Participants in intervention group also provided with a pedometer and a set of digital scales. | ActiGraph accelerometer used to monitor physical activity. Adverse events were collected during telephone interviews. Reported post-intervention medication adherence. |
| Harno  [2006] | Web-based database where patients uploaded their blood glucose readings with follow-up text messages from diabetes team. | Standard care including regular general practitioner visits approximately every 3 months | NR | NR | The home care link was provided free of charge to study patients. | NR |
| Holbrook  [2009] | Web-based diabetes tracker which included 13 diabetes risk factors with set targets. The tracker interfaced with clinician records and an automated telephone reminder system for patients. A tracker page was mailed 4 times per year and taken to physician appointments. | Usual care from respective primary care providers. | **Primary:** Improvement in composite score of relevant variables (HbA1c, LDL-c, BMI, albuminuria, blood pressure, foot checking, smoking, physical activity).  **Secondary:** 8-item composite score for clinical marker outcomes including quality of life, effect on perceived usefulness and continuity of care. | NR | NR | NR |
| Huo  [2019] | Text-messaging-based program with the automatic delivery of 6 text messages per week during the 6-month study period. One message per week was focused each on: disease information, glucose monitoring, blood pressure control, medication adherence, physical activity, and lifestyle changes. | Standard care and 2 thank you text messages (without risk factor modification support) per month. | **Primary:** Change in HbA1c.  **Secondary:** Change in proportion of patients achieving HbA1c, FBG, LDL-c, blood pressure, and physical activity targets. | **Blood pressure:** Measured using an electronic device (HEM-7111; Omron) and calculated as the average of 2 resting, seated measurements taken at least 5 minutes apart. If the difference between the 2 SBP or diastolic blood pressure readings was >5 mmHg, a third measurement was performed, and the mean value of the 3 readings was used.  **HbA1c and lipids:** Blood sample tests were analyzed at the central NCCD lab in Beijing by trained researchers according to manufacturer instructions. HbA1c was measured using HPLC with the ADAMS A 1C HA-8180 (ARKRAY, Inc, Japan). | NR | Acceptability and utility survey. Reported on number of people using cardio-protective medication and insulin at follow-up. |
| Kempf  [2017] | Telemedicine program including weekly ~20-minute calls with diabetes coaches about type 2 diabetes, medications, healthy diet and physical activity. Participants were advised to measure steps and weight daily. Devices automatically collected and transferred data into a personalized online portal. | Routine care (quarterly visits with their attending physician for routine health care visits as deﬁned by the Disease Management Programs [DMP] for Type 2 Diabetes in Germany). | **Primary:** Change in HbA1C from baseline to 12 months.  **Secondary:** Differences in body weight and composition, anti-diabetes medication, CVD risk factors, quality of life, and eating behaviours. | **Blood pressure:** Measured by attending physician at baseline. Blood pressure on both arms was determined using a mean of two measurements after a 5-min rest in a sitting position.  **HbA1c and lipids:** Venous blood sample and were analyzed at local laboratories. | Participants in both groups received a self-management guide, a weighing scale, and a step counter. | The medication effect  score (MES) based on the potency and dosage of anti-diabetes medication was calculated |
| Krein  [2004] | Intervention group assigned a case manager who was a nurse practitioner. Nurse practitioner would contact patients via telephone and scheduled follow-ups according to individual patient needs. Nurse practitioners would encourage patient self-management; reminders for recommended screenings/tests; help with appointment scheduling; monitor home glucose and blood pressure levels and identify and initiate medication and dose changes as needed. | Usual care from primary care providers. | **Primary:** Glycemic control (reported as HbA1c).  **Secondary:** LDL cholesterol and blood pressure. | **Blood pressure:** measured using an automated blood pressure machine.  **HbA1c and lipids:** HbA1c measured using Boronate affinity binding assay (Abbott IMx immunoassay system). | All study participants given an A&D Medical semi-automatic blood pressure monitor, home blood pressure monitoring guidelines, a lay version of the VA diabetes clinical guidelines and a periodic study newsletter. | Reported number of participants with medication changes and adherence to optimized medical therapy at follow-up. |
| Leichter  [2013] | Participants were provided with data management software for their blood glucose monitor, and a weight scale. Quarterly visits (two office and two phone (endocrinologist) to review monitored data. | Conducted self-monitoring of blood glucose at home, but were not instructed in transmitting blood glucose data via the Internet. Quarterly office visits to review monitored blood glucose data, and therapy adjustments made as needed. | **Primary:** HbA1c, BMI, systolic blood pressure, and lipids.  **Secondary:** Comparison of dropout rate and time spent during consultations. | **Blood pressure:** measured at each clinic visit for the control group; and for two of four visits for the intervention group, with two visits self-reported through internet and telephone.  **HbA1c and lipids:** measured at each clinic visit for the control group and for the intervention group, lipid laboratory values were obtained prior to internet interactions. | All participants received an Accu-Chek blood glucose meter and test strips. Intervention group additionally received an ACCU-CHEK 360 software and training to use this software, a bodyweight weight scale and a digital blood pressure cuff. | Adverse outcomes based on number experiencing hypoglycemia or hyperglycemia which did/did not require hospitalization. |
| Lim  [2021] | Usual care + single advisory session from registered research dietitian concerning diet and physical activity + intervention: Use of the app to track weight (2x weekly), diet and physical activity (daily) and for communication with research dietitian. Research dietitian would message participants via the app at least once weekly for the first 3 months, at a duration of between 1 to 15 minutes each. | Usual care defined as standard diabetes care from usual health care professionals + single advisory session from registered research dietitian concerning diet and physical activity. | **Primary:** Change in body weight 6 months post-intervention.  **Secondary:** Changes in body weight 3 months post-intervention, metabolic profiles (including HbA1c, fasting blood glucose, blood pressure, total cholesterol, triglycerides, low-density and high-density lipoprotein levels), creatinine levels and dietary intake. | **Blood pressure:** measured using automatic blood pressure monitor (Omron Healthcare) by research staff.  **HbA1c and lipids:** HbA1c, Total cholesterol, LDL, and HDL values were obtained via accredited pathology laboratories. | All participants were issued a standardized digital weighing scale (Omron Healthcare). All intervention group participants were provided with a glucometer (Abbott Laboratories). | NR |
| Liou  [2014] | Six monthly education sessions (four in-person and two through videoconferencing) covering diabetes, diet, medication, stress, goal setting, and foot care. | Usual care in addition to one individual diabetes education session by a licensed practical nurse. | NR | NR | NR | NR |
| Nicolucci  [2015] | Participants received a weight scale, blood pressure device, glucometer, and Bluetooth hub that transmitted data to a central system for use by their general practitioner. The system generated automated reminders, notifications and warnings for the practitioner or patient. Monthly calls by nurses discussed self-monitoring habits. | Usual care by general practitioner. | **Primary:** HbA1c at 6 and 12-months after randomisation.  **Secondary:** Changes in and % of patients with HbA1c target of <7%, blood pressure <130/80 mmHg, and LDL <100 mg/dL. Change in body weight, number of visits, change in medication and quality of life. | **Blood pressure:** NR  **HbA1c and lipids:** profiles were measured in a centralized laboratory. | NR | Reported medication adherence at follow-up. |
| Odnoletkova  [2016] | Nurse-led telephone coaching program to identify treatment gaps and achieve guideline targets, adjust lifestyle habits and adherence to medications. Five phone sessions of ~30 minute duration every 5 weeks. Participants also received a nutrition guide, waist circumference meter, BMI calculator, blood glucose monitor and reports sent to GPs. | Usual care by general practitioners including diabetes education, and annual consultation with an endocrinologist. | **Primary:** Change in HbA1c from baseline to 6-months.  **Secondary:** Change in HbA1c from baseline to 18-months, and proportion of those achieving HbA1c, cholesterol, blood pressure, and BMI targets. Change in self-perceived health status. | **Blood pressure:** Taken using a manual sphygmomanometer when participant is in a sitting position with two measurements taken.  **HbA1c and lipids:** HbA1c analyzed using ion exchange chromatography, TC by enzymatic colorimetric methods, HDL through neutralization of LDL and VLDL, LDL by the Friedewald equation. | All participants received a DVD with educational materials on Type 2 diabetes. Lab results were mailed to participants and their GPs. | NR |
| Quinn [2011] | Intervention group received automated, real-time educational and behavioural messaging in response to individually analyzed blood glucose values, diabetes medications and lifestyle behaviours communicated by mobile phone. | Usual care defined as provider-driven care that is office-based with no special diabetes management. Patients also self-monitored their blood glucose. | **Primary:** Change in HbA1c over a 12-month period.  **Secondary:** Changes in patient-reported diabetes symptoms, diabetes distress, depression and other clinical (blood pressures) and laboratory (lipid) values. | **Blood pressure:** measurements were obtained from the GP’s office records.  **HbA1c and lipids:** Lipid measurements were obtained from the GP’s office records. | Participants in the intervention group received mobile phones, 1-year unlimited data and service plan, study mobile diabetes management software and access to the web-based portal. All patients received a One Touch Ultra 2 (LifeScan, Milpitas, CA) glucose meter and supplies. | NR |
| Ramallo-Farina [2020] | Intervention group received a group educational program (3-monthly face-to-face group sessions; 8 sessions total), and completed a web-based questionnaire weekly and an expanded questionnaire monthly. Text messages were delivered to participant’s mobile based on information provided in the questionnaire. | Usual care defined as usual care for type 2 diabetes received in primary health care. | **Primary:** Mean change in HbA1c levels from baseline to 24 months of follow-up.  **Secondary:** Changes in BMI, weight, waist circumference, waist-to-hip ratio, SBP and DBP from baseline to 3, 6, 12, 18 and 24 months. Changes in HDL, LDL, triglycerides and fasting glucose from baseline to 6, 12 and 24 months. Changes in serum creatinine and GFR from baseline to 12 and 24 months. | **Blood pressure:** measured twice in one arm (right when possible) in a sitting position, with a digital sphygmomanometer OMRON model M6 and the average of two readings recorded.  **HbA1c and lipids:** HbA1c quantified based on the Diabetes Control and Complications Trial assay. LDL estimated via Friedewald formula. | Intervention group received a paper-supported or web-based workbook. Workbook was used for gathering daily information. Intervention group also received a free phone service to fill in the online workbooks. | NR |
| Shahid [2014] | Intervention group received 8 phone calls over a period of 4 months by the principal investigators. The topics covered during the phone calls were questions on self-monitoring of blood glucose, medication intake, physical activity and healthy eating. The study team contacted the participant via mobile phone. | Usual care defined as being seen by healthcare professionals and receiving services according to the standard guidelines of managing diabetes. | **Primary:** Attaining HbA1c ≤ 7.0%.  **Secondary:** Low Density Lipoprotein (LDL) <100mg/dl in the intervention group. | NR | Both groups of participants were trained to use a self-monitored blood glucose form and the correct use of a glucometer. Written information leaflets were also provided to all participants and include information relating to diet, health lifestyles, hypo and hyperglycemia symptoms and diabetic complications. | NR |
| Shea  [2009] | Participants received a computer, web camera, home glucose meter and blood pressure machine. Participants could access their own clinical data and educational site. Nurses case managers delivered the videoconferences and were supervised by an endocrinologist. | Usual clinical care from primary care providers including periodic mailings. | **Primary:** Change in HbA1c, LDL-c, and blood pressure from baseline to 5-years.  **Secondary:** NR | **Blood pressure:** Resting blood pressure measured using a Dinimap Monitor Pro 100, with three measurements after 5 minutes of rest, using the average of the second and third measurement.  **HbA1c and lipids:** HbA1c was analyzed by boronic affinity chromatography, and lipids using enzymatic colorimetric methods. | Educational webpage was in both English and Spanish and had regular and low-literacy versions. Nurses also contact primary care physicians if it was determined that a change in management was needed for the participant. | NR |
| Tang  [2013] | Intervention group uploaded home glucometer readings and logged their dietary intake, physical activity, blood pressure, insulin record and weight. The study’s dietitian and nurse care manager contacted the participant via online messaging and provided clinical feedback, with medication management if needed. Intervention group also received personalized text and video educational nuggets dispensed by the care team. | Usual care defined as standard-of-care treatment, including reminders about annual and preventive guideline-based laboratory tests and screening. | **Primary:** Glucose control, measured by A1c, over a 12-month period.  **Secondary:** Blood pressure, LDL cholesterol, 10-year Framingham cardiovascular risk, satisfaction and psychosocial well-being. | NR | Participants in the intervention group received a diabetes status report, which provides an organized and consolidated summary of key parameters of diabetes care | NR |
| Varney  [2014] | Usual diabetes care + 6 months of telephone coaching by dietitian with experience in type 2 diabetes and CVD. Participants received an average of 6 coaching sessions and coaching sessions varied in duration. Advice given during coaching sessions consistent with Australian guidelines. | Usual diabetes care which included 3-6 monthly visit to a diabetes clinic staffed by endocrinologists, diabetes educators and dietitians. Participants also visited their GP at their own discretion. | **Primary:** HbA1c at 6 months.  **Secondary:** HbA1c at 12 months, 6- and 12-month adjusted mean fasting glucose, lipids, blood pressure, weight, waist circumference, body mass index, physical activity and Kesler Psychological Distress Scale score. | NR | Participants in the intervention group and their GP received a letter summarizing the participant’s goals following each coaching session. | Researcher-generated questions were used to determine adherence to guideline recommendations. |
| Vinitha  [2019] | 2–3 educatory text messages per week as reinforcement to healthy lifestyle practices and adherence to medication. Text message contents were on the causes, principles of diabetes management and associated complications. Text messages on the beneﬁts of healthy lifestyle, pros and cons of modifying and not changing the behaviour. | Standard care | **Primary:** Improvement in glycaemia measured by an HbA1c value of <7% (53 mmol/mol).  **Secondary:** Changes in fasting plasma glucose, 2-hour plasma glucose, lipid, body weight, waist circumference, blood pressure, physical activity, quality of life. | **Blood pressure:** NR  **HbA1c and lipids:** HbA1c by immunoturbidimetry. Lipid parameters were  estimated at the central laboratory. | NR | NR |
| Wild  [2016] | Telemonitoring program where intervention group transmit their blood pressure, blood glucose and weight readings to password-protected servers for primary care nurses to check and to organize treatment changes based on national guidelines if required. | Diabetes management in a general practice setting. In the UK, GPs are financially incentivized if patients meet targets for glycemic and blood pressure control. Well-controlled patients are reviewed at least annually, with more frequent reviews performed for people with poor glycemic or blood pressure control. | **Primary:** Adjusted mean difference in HbA1c.  **Secondary:** Adjusted mean difference in daytime ambulatory systolic and diastolic blood pressures and weight between treatment groups. | **Blood pressure:** Ambulatory blood pressure monitoring by research nurses.  **HbA1c and lipids:** Based on blood samples collected by research nurses. | A modem was supplied to the intervention group for transmission of readings to a remote secure server. | Adverse outcomes were extracted from participants’ electronic GP records. Medication adherence reported. |
| Yoo  [2009] | Intervention group provided with Ubiquitous Chronic Disease Care (UCDC) system. The UCDC comprised a patient-based cellphone which transmitted patient self-measured data e.g. body weight, exercise fulfillment physician. Physician then provides feedback and individualized recommendations to patient via text-message. | Usual care which is defined as visiting the outpatient clinic according to patient’s routine schedule and receiving usual out-patient treatment from their physicians. | NR | **Blood pressure:** NR  **HbA1c and lipids:** Serum total cholesterol, triglycerides and high-density lipoproteins-cholesterol were determined by enzymatic methods using a chemical analyzer (Hitachi 747, Tokyo, Japan). HbA1c was measured using high-performance liquid chromatography using a Variant II analyzer (Bio-Rad Laboratories, Hercules, CA, USA). | Patients in the intervention groups received a cellular phone (LG-SV280; LG Electronics, Seoul, Korea) with a modular blood glucose measuring device (Anycheck; Insung Information Co., Seoul, Korea), strips, and lancets. They also received an automatic blood pressure monitoring device (T5M; Omron, Kyoto, Japan), as well as body weight scales (HD308; Tanita, Tokyo, Japan). | NR |
| Zhou  [2014] | Intervention group used a Diabetes Telemedicine system which allowed patients to enter data relating to: capillary glucose, blood pressures, body weight etc. Staff members would analyse the data and provide guidance to the participants through the internet, short messages or telephone. | Usual care defined as patients visiting outpatient department as usual. | NR | NR | All participants also attended a 30-60 minute educational session for diabetes self-management and nutrition and received a pamphlet of diabetes management and a schedule of diabetes courses. | NR |

**Legend:** HbA1c= glycated hemoglobin A1c, TC= total cholesterol, LDL-c= low density lipoprotein cholesterol, SBP= systolic blood pressure, DBP= diastolic blood pressure.

# Supplementary Table 6: Summary of key aspects of the telehealth interventions in each randomized controlled trial.

| **Study** | **Focus areas for types of interventions utilized** | | | | | | | | | | |
| --- | --- | --- | --- | --- | --- | --- | --- | --- | --- | --- | --- |
|  | **PATIENT EDUCATION** | | **MONITORING OF RISK FACTORS** | | **COACHING REGARDING RISK FACTOR MODIFICATION** | | | **CONSULTATION** | | **PHARMACOLOGICAL MANGEMENT** | |
|  | **Individual education for achieving risk factor modification** | **Group education for achieving risk factor modification** | **Home based monitoring of risk factors/ activity** | **System for remote risk factor monitoring by treating team** | **Behavioural interventions focused on risk factors** | **Lifestyle interventions specifically focused on diet and exercise modification** | **Interventions to improve medication use** | **Review by physician or health care professional** | **Reminders about medical appointments** | **Pharmacotherapy based interventions (medication dose and class changes)** | **Medication reminders** |
| Aytekin Kanadli [2016] | Y (phone) | Y (in-person) |  |  | Y (phone reminder) |  |  |  |  |  |  |
| Blackberry [2013] | Y (phone) |  |  |  | Y (phone reminder) |  | Y (phone) | Y (In-person with primary care doctor) | Y (phone) | Y (recommendations made to treating team) |  |
| Bond  [2007]* | Y (internet) | Y (internet discussion board) | Y (BSL, BP, PA, weight) | Y (participant entered data logs) | Y (feedback by nurses via internet) |  |  |  |  |  |  |
| Crowley  [2013] | Y (phone) |  |  |  | Y (phone reminder) |  | Y (phone) | Y (In-person with primary care doctor) |  | Y (recommendations made to treating team) | Y (phone) |
| Davis  [2010] | Y (video conferencing) | Y (in-person initially and remote video conferencing) | Y  (BSL and PA) | Y (participant entered data logs) | Y  (feedback by video conferencing) | Y  (video conferencing) | Y  (video conferencing) | Y (video conferencing by nurse/educator/dietitian) |  |  |  |
| de Vasconcelos [2018] | Y (phone) |  |  |  | Y (via phone) |  | Y (phone) | Y (phone calls with tele-nurse) |  |  |  |
| Eakin  [2014] | Y (phone) |  | Y (PA, weight) |  |  | Y (phone reviews of diet) |  |  |  |  |  |
| Harno  [2006] |  |  | Y (BSL) | Y (automated data transfer to database) | Y (SMS) |  |  |  |  |  |  |
| Holbrook  [2009] |  |  | Y (BP, PA, weight) | Y (electronic tracker integrated into medical record) | Y (automated voice message and online reminders) | Y (online reminders for exercise and weight loss) |  |  | Y (automated voice message) | Y (online recommendations to see physician) | Y (automated voice message) |
| Huo  [2019] | Y (automated SMS) |  |  |  | Y (automated SMS) | Y (automated SMS health messages) | Y (automated SMS) |  |  |  |  |
| Kempf  [2017] | Y (phone) |  | Y (BSL, BP, PA, weight) | Y (automated transfer to personalized online portal) | Y (phone) | Y (phone dietary intervention and advise on exercise) | Y (phone calls) | Y (phone calls from trained diabetes coaches) |  |  |  |
| Krein  [2004] | Y (phone) |  | Y (BP, BSL) |  | Y (phone) | Y (phone) |  | Y (phone calls from case manager) | Y (phone) | Y (medication treatment algorithms) |  |
| Leichter  [2013] |  |  | Y (BSL, BP, weight) | Y (stored on the web and reviewed by endocrinologist) |  |  |  | Y (remote review by endocrinologist) |  | Y (email and phone) |  |
| Lim  [2021] | Y (short educational videos via APP) |  | Y (BSL and weight) | Y (via APP) | Y (via APP) | Y (individualized program via APP) |  | Y (remote consultation by dietitian via the APP) |  |  |  |
| Liou  [2014] | Y (in person, videoconferencing, and internet) |  |  |  | Y (via internet and videoconferencing) | Y (in-person and internet) | Y (via internet and videoconferencing) | Y (shared care team  by interactive videoconferencing) |  |  |  |
| Nicolucci  [2015] | Y (SMS, email or phone) |  | Y (BSL, BP, weight) | Y (automated data transfer to centralized internet database) | Y (automated messages by SMS, email or phone) |  | Y (SMS, email or phone) | Y (phone call once a month by tele-health nurse) |  |  | Y (SMS, email or phone) |
| Odnoletkova  [2016] | Y (DVD, phone education) |  | Y (BSL) |  | Y (phone calls with diabetes nurse educator) | Y (nutrition guide provided in-person) | Y (phone) | Y (phone calls with tele-coach diabetes educator) | Y (phone) | Y (recommendations made to discuss with GP) |  |
| Quinn  [2011] | Y (automated SMS) |  | Y (BSL) | Y (automated data transfer to mobile app and web portal) | Y (automated SMS and individualized messages) |  | Y (mobile-based coaching) | Y (phone calls to educators as needed or contact via email) |  | Y (recommendations via quarterly reports to GP) |  |
| Ramallo-Farina [2020] |  | Y (in-person) | Y (BSL, BP, PA) | Y (manual entry into web portal) | Y (semi-automated SMS) |  | Y (semi-automated SMS) |  |  | Y (recommendations given to GP) |  |
| Shahid  [2014] | Y (phone) |  | Y (BSL) |  | Y (phone-based feedback) | Y (in-person nutrition information given) | Y (phone) |  |  |  |  |
| Shea  [2009] |  | Y (web page) | Y (BP, BSL) | Y (automated data transfer to home tele-health unit) | Y (via videoconferencing) |  | Y (videoconferencing) | Y (videoconferencing with tele-nurse) |  | Y (recommendations made to GP) |  |
| Tang  [2013] | Y (via internet ) |  | Y (BSL) | Y (automated data transfer to electronic health record) | Y (brief texts or videos) | Y (nutrition and exercise logs provided remotely) | Y (via internet report) | Y (remotely with nurse care managers) | Y (via internet report) | Y (made by study team) |  |
| Varney  [2014] | Y (phone) |  |  |  | Y (via phone) | Y (phone advice from dietitian) |  |  | Y (via phone) | Y (recommendations made to discuss with GP) |  |
| Vinitha  [2019] | Y (SMS) |  |  |  |  | Y (in-person healthy diet pattern and PA advice) | Y (SMS) |  |  |  | Y (SMS) |
| Wild  [2016] |  |  | Y (BSL, BP, weight) | Y (automated data transfer to remote secure server shared with GP) | Y (web-based reports) |  |  |  |  | Y (made by study team) |  |
| Yoo  [2009] | Y (SMS) |  | Y (BSL, BP, weight and PA via SMS) | Y (automated transfer of data to web portal) | Y (automated alerts) | Y (automated alerts regarding PA via SMS) |  |  |  |  |  |
| Zhou  [2014] | Y (internet web based) |  | Y (BSL, PA) | Y (manual upload by participant to telemedicine system) | Y (internet based self-education or phone) |  |  |  |  |  |  |

**Legend:** Data are presented as Y= yes (this was included in the study). SMS = text messaging, BP= blood pressure, BSL= blood sugar level, PA= physical activity, APP= mobile phone application, GP= general practitioner/ primary care physician. The green highlighted areas indicate remotely delivered interventions, whereas the yellow-colored boxes represent interventions delivered in-person and red-colored boxes represent interventions that were not included as a part of the trial. *Bond et al. (2007) recorded medication data remotely but did not make changes to these based on the description of their intervention. Davis et al. (2010) offered retinal imaging in the primary-care setting when participants were due for their annual eye exam, which is not captured in the above table. For Lim et al. (2020) both the treatment group and control group received an initial in-person education session regarding diet and physical activity. Although Blackberry et al. (2013) included remote pharmacotherapy, only 18% of their remote consultations actually addressed intensification of hypoglycemic agents and Crowley et al. (2013) stated that primary care providers replied to 76% (332/436) of the contacts, and of these replies, 18% (59/332) resulted in medication change recommendations.

# Supplementary Table 7: Risk of bias assessment for each included study using the Cochrane Collaboration updated Risk of Bias 2 (RoB-2) tool

| **RoB-2 Tool criterion** | **STUDY** | | | | | | | | | | | | | | | | | | | | | | | | | | |
| --- | --- | --- | --- | --- | --- | --- | --- | --- | --- | --- | --- | --- | --- | --- | --- | --- | --- | --- | --- | --- | --- | --- | --- | --- | --- | --- | --- |
| **Risk of bias assessment area or question** | Aytekin Kanadli  [2016] | Blackberry  [2013] | Bond  [2007] | Crowley  [2013] | Davis  [2010] | de Vasconcelos [2018] | Eakin  [2014] | Harno  [2006] | Holbrook  [2009] | Huo  [2019] | Kempf  [2017] | Krein  [2004] | Leichter  [2013] | Lim [2021] | Liou  [2014] | Nicolucci  [2015] | Odnoletkova  [2016] | Quinn [2011] | Ramallo-Farina [2020] | Shahid [2014] | Shea  [2009] | Tang [2013] | Varney  [2014] | Vinitha  [2019] | Wild  [2016] | Yoo  [2009] | Zhou [2014] |
| 1.1 Was allocation sequence random? | Y/PY | Y | Y | Y | NI | PY | Y | NI | Y | Y | Y | Y | N | Y | NI | Y | Y | Y | Y | NI | Y | Y | Y | Y | Y | NI | Y |
| 1.2 Was allocation sequence concealed until participants were assigned to intervention/ control? | NI | Y | NI | Y | NI | NI | NI | PN | Y | NI | Y | NI | N | Y | NI | NI | NI | Y | Y | NI | NI | NI | Y | Y | N | NI | NI |
| 1.3 Did baseline differences between groups suggest a problem with randomisation? | N | N | N | N | N | N | N | Y | N | N | N | PY | PY | N | PY | N | N | PY | N | Y | N | N | N | N | N | N | N |
| **1. Risk of bias judgement** | **Some** | **Low** | **Some** | **Low** | **Some** | **Some** | **Some** | **High** | **Low** | **Some** | **Low** | **High** | **Some** | **Low** | **High** | **Some** | **Some** | **Some** | **Low** | **High** | **Some** | **Some** | **Low** | **Low** | **High** | **Some** | **Some** |
| 2.1 Were participants aware of their assigned intervention during the trial? | PY/Y | Y | PY/Y | Y | PY/Y | Y | Y | PY/Y | PY/Y | Y | PY/Y | PY/Y | PY/Y | Y | PY/Y | PY/Y | Y | Y | Y | Y | PY/Y | Y | Y | PY/Y | Y | Y | Y |
| 2.2 Were carers and people delivering the interventions aware of participants' assigned intervention during the trial? | PY/Y | Y | PY/Y | Y | PY/Y | Y | Y | Y | Y | PN/N | PY/Y | PY/Y | Y | Y | Y | Y | Y | Y | Y | Y | Y | Y | Y | Y | Y | Y | Y |
| 2.3. If Y/PY/NI to 2.1 or 2.2:  Were there deviations from the intended intervention that arose because of the trial context? | N | N | N | N | N | N | N | N/PN | N | N | N | N | N | N | NI | N | N | N | N | N | N | N | PN | N | PN | N | N |
| 2.4 If Y/PY/NI to 2.3: Were these deviations likely to have affected the outcome? | **-** | **-** | - | - | - | - | - | - | - | - | - | - | **-** | - | -/PN | - | - | - | - | - | - | - | - | - | **-** | - | - |
| 2.5. If Y/PY to 2.4: Were these deviations from intended intervention balanced between groups? | **-** | **-** | **-** | **-** | - | **-** | **-** | **-** | **-** | **-** | **-** | **-** | **-** | **-** | **-** | **-** | **-** | **-** | **-** | **-** | **-** | **-** | **-** | **-** | **-** | **-** | **-** |
| 2.6 Was an appropriate analysis used to estimate the effect of assignment to intervention? | NI | Y | NI | NI | NI | NI | NI | NI | Y | Y | Y | Y | N | Y | NI | N | Y | Y | Y | PN | Y | Y | NI | Y | Y | Y | NI |
| 2.7 If N/PN/NI to 2.6: Was there potential for a substantial impact (on the result) of the failure to analyse participants in the group to which they were randomized? | PN | - | PN | N | N | N | N | PN | - | - | - | - | N/PN | - | PN | PY | - | - | - | NI | - | - | PN | - | **-** | - | PN |
| **2. Risk of bias judgement** | **High** | **Low** | **High** | **Some** | **Some** | **Some** | **Some** | **High** | **Low** | **Low** | **Low** | **Low** | **Some** | **Low** | **Some** | **High** | **Low** | **Low** | **Low** | **High** | **Low** | **Low** | **Some** | **Low** | **Low** | **Low** | **Some** |
| 3.1 Were data for this outcome available for all, or nearly all, participants randomized? | Y | N | Y | PY | Y | N | Y | NI | N | Y | PN/N | Y | N | Y | NI | N | N | N | N | Y | PN/N | Y | N | PY/Y | Y | Y | Y |
| 3.2 If N/PN/NI to 3.1: Is there evidence that the result was not biased by missing outcome data? | **-** | Y | - | - | - | N | - | PY | PY | - | PN/N | - | PN/N | - | Y/- | N | PN | Y | Y | - | Y | - | PY | - | **-** | - | - |
| 3.3 If N/PN to 3.2: Could missingness in the outcome depend on its true value? | **-** | **-** | **-** | **-** | - | NI | **-** | **-** | **-** | **-** | NI | **-** | NI | **-** | **-** | PY/Y | PY | **-** | **-** | **-** | **-** | **-** | **-** | **-** | **-** | **-** | **-** |
| 3.4 If Y/PY/NI to 3.3: Is it likely that missingness in the outcome depended on its true value? | **-** | **-** | **-** | **-** | - | NI | **-** | **-** | **-** | **-** | Y | **-** | PN | **-** | **-** | PY | NI | **-** | **-** | **-** | **-** | **-** | **-** | **-** | **-** | **-** | **-** |
| **3. Risk of bias judgement** | **Low** | **Low** | **Low** | **Low** | **Low** | **High** | **Low** | **Low** | **Low** | **Low** | **High** | **Low** | **Some** | **Low** | **Low** | **High** | **High** | **Low** | **Low** | **Low** | **Low** | **Low** | **Low** | **Low** | **Low** | **Low** | **Low** |
| 4.1 Was the method of measuring the outcome inappropriate? | N/PN | N | N/PN | N | N | N | N | N | N | N | N/PN | N | N | N | N | N | N | N | N | N | N | N | PN | N/PN | N | N | N |
| 4.2 Could measurement or ascertainment of the outcome have differed between intervention groups? | N/PN | N | N/PN | N | N | N | N | Y | N/PN | N | N | N | Y | N | N | N | N | N | N | N | N | N | PN | N | N | N | PY |
| 4.3 If N/PN/NI to 4.1 and 4.2: Were outcome assessors aware of the intervention received by study participants? | NI | Y | N | Y | NI | Y | N | - | N | N | N | NI | -/Y | PN | NI | NI | N | Y | N | PY | N | N | Y | N | Y | NI | - |
| 4.4 If Y/PY/NI to 4.3: Could assessment of the outcome have been influenced by knowledge of intervention received? | N/PN | PN | N/- | PN | N/PN | N | - | - | - | - | - | N/PN | -/Y | - | PY | N/PN | N/- | PN | - | N | N/- | - | PN | - | PN | N | - |
| 4.5 If Y/PY/NI to 4.4: Is it likely that assessment of the outcome was influenced by knowledge of intervention received? | - | - | **-** | - | - | - | **-** | - | **-** | **-** | **-** | **-** | -/PN | - | NI | **-** | **-** | - | - | - | **-** | - | **-** | **-** | - | - | - |
| **4. Risk of bias judgement** | **Low** | **Low** | **Low** | **Low** | **Low** | **Low** | **Low** | **High** | **Low** | **Low** | **Low** | **Low** | **High** | **Low** | **High** | **Low** | **Low** | **Low** | **Low** | **Low** | **Low** | **Low** | **Low** | **Low** | **Low** | **Low** | **High** |
| 5.1 Were the data that produced this result analysed in accordance with a pre-specified analysis plan that was finalised before unblended outcome data were available for analysis? | NI | Y | NI | Y | NI | N | Y | NI | Y | Y | NI | NI | NI | N | NI | NI | Y | Y | Y | N | Y | N | Y | NI | Y | N | N |
| 5.2 Is the numerical result being assessed likely to have been selected, on the basis of the results from multiple eligible outcome measurements (e.g. scales, definitions, time points) within the outcome domain? | N | N | N | N | N | N | N | N | N/PN | N/PN | N | N | N | N | N/PN | N | N | N | N | N | N | N | N | N | N | N | N |
| 5.3 Is the numerical result being assessed likely to have been selected, on the basis of the results from multiple eligible analyses of the data? | N | N | N | N | N | N | N | PN | N/PN | N/PN | N | N | N | N | N/PN | N | N | N | N | N | N | N | N | N | N | N | N |
| **5. Risk of bias judgement** | **Some** | **Low** | **Some** | **Low** | **Some** | **Some** | **Low** | **Some** | **Low** | **Low** | **Some** | **Some** | **Some** | **Some** | **Some** | **Low** | **Low** | **Low** | **Low** | **Some** | **Low** | **Some** | **Low** | **Some** | **Low** | **Some** | **Some** |
| **Overall risk of bias judgement** | **High risk** | **Low risk** | **High risk** | **Some concerns** | **Some concerns** | **High Risk** | **Some concerns** | **High Risk** | **Low risk** | **Some concerns** | **High Risk** | **Some concerns** | **High Risk** | **Some concerns** | **High Risk** | **High Risk** | **High Risk** | **Some concerns** | **Low risk** | **High Risk** | **Some concerns** | **Some concerns** | **Some concerns** | **Some concerns** | **High Risk** | **Some concerns** | **High Risk** |

**Legend:** The possible outcomes were ‘yes’ (Y), ‘probably yes’ (PY), ‘no information’ (NI), ‘probably no’ (PN), or ‘no’ (N). Where a final consensus could not be made on a particular prompt, the individual ratings of the two authors are reported as author 1/author 2 where the ratings were not mutually exclusive. Overall consensus was made where the two scores were mutually exclusive.

# Supplementary Table 8: Effect of intervention versus control on the three primary outcomes at the last follow-up.

| **Study** | **Number Randomized:**  **Number Completed** | **Intervention group**  **(n=)** | **Control group**  **(n=)** | **Post-intervention HbA1c (%)** | | **Post-intervention systolic blood pressure (mmHg)** | | **Post-intervention diastolic blood pressure (mmHg)** | | **Post-intervention LDL-C (mmol/L)** | | **Post-intervention TC (mmol/L)** | |
| --- | --- | --- | --- | --- | --- | --- | --- | --- | --- | --- | --- | --- | --- |
|  |  |  |  | **Intervention** | **Control** | **Intervention** | **Control** | **Intervention** | **Control** | **Intervention** | **Control** | **Intervention** | **Control** |
| Aytekin Kanadli  [2016] | 91:88 | 47:44 | 44:44 | 7.5  (0.7) | 7.9  (1.5) | 126  (13) | 130  (15) | 82  (10) | 82  (9) | 2.98  (0.92) | 3.29  (1.00) | 4.94  (1.59) | 5.93  (1.46) |
| Blackberry  [2013] | 473:440 | 236:221 | 237:219 | 7.9  (1.2) | 7.9  (1.4) | 133  (14) | 136  (16) | 76  (9) | 77  (11) | 2.22  (0.87) | 2.26  (0.84) | 4.20  (0.95) | 4.28  (1.05) |
| Bond  [2007] | 62:62 | 31:31 | 31:31 | 6.4  (1.2) | 7.1  (1.0) | 128  (13) | 131  (10) | 70  (7) | 73  (7) | NR | NR | 4.27  (0.98) | 4.54  (0.96) |
| Crowley  [2013] | 359:330 | 182:166 | 177:164 | 7.8  (1.3) | 7.9  (1.3) | 138  (24) | 133  (17) | NR | NR | 2.32  (1.64) | 2.67  (1.57) | NR | NR |
| Davis  [2010] | 165:165 | 85:85 | 80:80 | 8.2  (3.7) | 8.6  (2.7) | 128  (37) | 131  (34) | 70  (20) | 71  (20) | 2.32  (1.61) | 2.67  (1.61) | NR | NR |
| de Vasconcelos [2018] | 36:31 | 18:16 | 18:15 | 7.2  (1.2) | 7.3  (1.7) | 126  (14) | 141  (24) | 71  (8) | 76  (9) | 2.53  (0.93) | 2.60  (0.79) | 4.56  (1.17) | 4.49  (1.13) |
| Eakin  [2014] | 302:249 | 151:118 | 151:131 | 7.4  (1.3) | 7.6  (1.6) | 133  (16) | 131  (15) | 79  (9) | 79  (9) | 2.35  (0.88) | 2.16  (0.81) | NR | NR |
| Harno  [2006] | 175:NR | 101:NR | 74:NR | 7.3  (1.1) | 7.8  (1.7) | 135 (22) | 137  (20) | 79  (11) | 82  (13) | 2.52  (0.80) | 2.76  (0.86) | 4.74  (1.11) | 5.03  (1.03) |
| Holbrook  [2009] | 511:445 | 253:224 | 258:221 | 6.8  (1.2) | 7.3  (1.6) | 131  (16) | 135  (18) | 74  (10) | 75  (11) | 2.43  (0.78) | 2.54  (0.81) | NR | NR |
| Huo  [2019] | 502:468 | 251:234 | 251:234 | 6.7  (1.3) | 7.2  (1.5) | 135  (19) | 132  (18) | NR | NR | 2.5  (0.70) | 2.5  (0.80) | NR | NR |
| Kempf  [2017] | 202:133 | 102:77 | 100:56 | 7.6  (1.2) | 8.2  (1.3) | 136  (17) | 133  (12) | 80  (10) | 79  (9) | 3.03  (1.16) | 2.95  (0.93) | 4.97  (1.06) | 4.89  (1.16) |
| Krein  [2004] | 246:209 | NR: 106 | NR: 103 | 9.3  (2.1) | 9.2  (2.0) | 146  (24) | 144  (22) | 83  (12) | 83  (11) | 2.74  (0.85) | 2.82  (0.87) | NR | NR |
| Leichter  [2013] | 98:70 | 49:33 | 49:37 | 7.4  (1.1) | 7.1  (1.2) | 135  (16) | 133  (16) | 79  (7) | 77  (7) | 2.06  (0.71) | 2.35  (0.71) | NR | NR |
| Lim  [2021] | 204:195 | 99:94 | 105:101 | 6.7  (1.0) | 7.1  (1.1) | 131  (12) | 135  (13) | 79  (8) | 84  (8) | 2.41  (0.84) | 2.54  (0.76) | 4.33  (0.99) | 4.51  (0.86) |
| Liou  [2014] | 95:95 | 54:54 | 41:41 | 7.6  (1.1) | 8.1  (1.3) | 130 (13) | 132  (15) | 80  (11) | 78  (9) | 2.70  (0.84) | 2.97  (1.00) | 4.64  (1.01) | 4.95  (0.95) |
| Nicolucci  [2015] | 302:249 | 153:114 | 149:135 | 7.4  (1.0) | 7.8  (1.1) | 136  (10) | 136  (12) | 80  (7) | 79  (7) | 3.05  (0.85) | 2.95  (0.78) | 4.76  (1.03) | 4.63  (0.91) |
| Odnoletkova  [2016] | 574:487^†^ | 287:241^†^ | 287:246^†^ | 6.9  (1.0) | 7.0  (1.1) | 128  (14) | 130  (15) | 75  (9) | 76  (9) | 2.15  (0.75) | 2.25  (0.78) | 4.19  (0.88) | 4.40  (1.27) |
| Quinn  [2011] | 213:163 | 38:23‡ | 62:56 | 7.7  (1.0) | 8.5  (1.8) | 134  (25) | 133  (20) | 82  (11) | 79  (13) | 2.43  (0.83) | 2.35  (0.88) | 3.91  (0.88) | 4.34  (1.03) |
|  |  | 33:22§ |  | 7.9  (1.4) |  | 134  (16) |  | 78  (9) |  | 2.43  (1.22) |  | 4.34  (1.34) |  |
|  |  | 80:62\|\| |  | 7.9  (1.7) |  | 128  (19) |  | 78  (10) |  | 2.64  (0.83) |  | 4.5  (1.09) |  |
| Ramallo-Farina [2020]^*^ | 1123:846 | 537:375 | 586:471 | 7.3  (1.4) | 7.3  (1.4) | 128  (16) | 130  (17) | 81  (10) | 83  (10) | 2.74  (0.88) | 2.68  (0.86) | 4.79  (1.03) | 4.72  (0.97) |
| Shahid  [2014] | 440:440 | 220:220 | 220:220 | 8.6  (1.3) | 9.4  (1.2) | 118  (9) | 120  (8) | 76  (7) | 75  (6) | 2.29  (0.47) | 2.57  (0.50) | NR | NR |
| Shea  [2009] | 1665:793 | 844:330 | 821:463 | 7.1  (1.2) | 7.3  (1.5) | 136  (20) | 140  (22) | 67  (10) | 69  (11) | 2.38  (0.89) | 2.44  (0.90) | 4.14  (0.92) | 4.72  (1.08) |
| Tang  [2013] | 415:379 | 202:186 | 213:193 | 8.1  (1.7) | 8.3  (1.8) | 120  (11) | 121  (12) | 72  (9) | 73  (8) | 2.30  (0.87) | 2.54  (0.84) | NR | NR |
| Varney  [2014] | 94:71 | 47:35 | 47:36 | 8.2  (1.1) | 8.4  (1.1) | 132  (14) | 138  (14) | 77  (9) | 80  (9) | 2.2  (0.8) | 2.5  (0.8) | 4.2  (0.9) | 4.4  (0.8) |
| Vinitha  [2019] | 248:218 | 126:112 | 122:106 | 7.4  (1.5) | 7.8  (1.5) | 124  (8) | 128  (12) | 82  (6) | 82  (7) | 2.6  (0.9) | 2.7  (0.9) | 4.6  (1.1) | 4.7  (1.0) |
| Wild  [2016] | 321:285 | 160:146 | 161:139 | 7.9  (1.4) | 8.4  (1.3) | 131  (12) | 134  (11) | 76  (9) | 78  (9) | NR | NR | 4.1  (0.9) | 4.3  (1.2) |
| Yoo  [2009] | 123:111 | 62:57 | 61:54 | 7.1  (0.8) | 7.6  (1.0) | 133  (16) | 134  (14) | 80  (9) | 82  (8) | 2.2  (0.6) | 2.3  (0.7) | 4.1  (0.7) | 4.5  (0.8) |
| Zhou  [2014] | 114:108 | 57:53 | 57:55 | 6.8  (1.2) | 7.6  (1.6) | 125  (12) | 129  (12) | 77  (8) | 75  (7) | 2.77  (0.99) | 2.63  (0.82) | 4.91  (1.00) | 4.70  (0.84) |

**Legend:** Data are presented as raw numbers, or mean (standard deviation) unless otherwise specified. NR = not reported. ^*^ Ramello-Farina et al. (2020) had several interventional groups and only data from the ‘patient intervention’ group were included

^†^ For Odnoletkova et al. (2016) the number of participants for whom outcome data was reported in the two groups differed; HbA1c 240 vs 246 participants, LDL-c 237 vs 239 participants and SBP/DBP 241 vs 247 participants. Quinn et al (2011) had three intervention groups, all of which are reported: ‡ online coaching only, § coaching and primary care provider portal, and || coach primary care provider portal with decision-support. The control group was split into three groups for purposes of meta-analysis to include all three intervention arms.

# Supplementary Table 9: Data on the secondary outcomes of interest reported in the included randomized controlled trials.

| **Study** | **Total adverse outcomes** | | **MACE** | | **Limb amputation/ Re-vascularization** | | **Hospitalization** | | **Hypoglycemia** | | **Hypotension** | | **Other intervention related adverse event** | | **Death** | | **Microvascular disease progression** | |
| --- | --- | --- | --- | --- | --- | --- | --- | --- | --- | --- | --- | --- | --- | --- | --- | --- | --- | --- |
|  | Intervention | Control | Intervention | Control | Intervention | Control | Intervention | Control | Intervention | Control | Intervention | Control | Intervention | Control | Intervention | Control | Intervention | Control |
| Aytekin Kanadli  [2016] | NR | NR | NR | NR | NR | NR | NR | NR | NR | NR | NR | NR | NR | NR | 0 | 0 | NR | NR |
| Blackberry [2013] | 4 | 4 | NR | NR | NR | NR | NR | NR | NR | NR | NR | NR | NR | NR | 4 | 4 | NR | NR |
| Bond  [2007] | NR | NR | NR | NR | NR | NR | NR | NR | NR | NR | NR | NR | NR | NR | 0 | 0 | NR | NR |
| Crowley [2013] | 2 | 2 | NR | NR | NR | NR | NR | NR | NR | NR | NR | NR | NR | NR | 2 | 2 | NR | NR |
| Davis  [2010] | NR | NR | NR | NR | NR | NR | NR | NR | NR | NR | NR | NR | NR | NR | 0 | 0 | NR | NR |
| de Vasconcelos [2018] | NR | NR | NR | NR | NR | NR | NR | NR | NR | NR | NR | NR | NR | NR | NR | NR | NR | NR |
| Eakin  [2014] | 21 | 33 | NR | NR | NR | NR | 4 | 4 | 0 | 0 | NR | NR | 17 | 28 | 0 | 1 | NR | NR |
| Harno  [2006] | NR | NR | NR | NR | NR | NR | NR | NR | NR | NR | NR | NR | NR | NR | NR | NR | NR | NR |
| Holbrook  [2009] | 0 | 1 | NR | NR | NR | NR | NR | NR | NR | NR | NR | NR | NR | NR | 0 | 1 | NR | NR |
| Huo  [2019] | 3 | 1 | NR | NR | NR | NR | NR | NR | NR | NR | NR | NR | NR | NR | 3 | 1 | NR | NR |
| Kempf  [2017]^a^ | 0 | 0 | NR | NR | NR | NR | NR | NR | NR | NR | NR | NR | NR | NR | NR | NR | NR | NR |
| Krein  [2004] | 6 | 10 | NR | NR | NR | NR | NR | NR | NR | NR | NR | NR | NR | NR | 6 | 10 | NR | NR |
| Leichter  [2013] | 0 | 0 | NR | NR | NR | NR | NR | NR | NR | NR | NR | NR | NR | NR | NR | NR | NR | NR |
| Lim  [2021] | 3 | 0 | NR | NR | NR | NR | 0 | 0 | 3 | 0 | NR | NR | NR | NR | 0 | 0 | NR | NR |
| Liou  [2014] | NR | NR | NR | NR | NR | NR | NR | NR | NR | NR | NR | NR | NR | NR | NR | NR | NR | NR |
| Nicolucci  [2015]^a^ | 4 | 2 | NR | NR | NR | NR | NR | NR | NR | NR | NR | NR | NR | NR | 2 | 0 | NR | NR |
| Odnoletkova  [2016] | 2 | 5 | NR | NR | NR | NR | NR | NR | NR | NR | NR | NR | NR | NR | 1 | 4 | NR | NR |
| Quinn  [2011] | NR | NR | NR | NR | NR | NR | NR | NR | NR | NR | NR | NR | NR | NR | 0 | 0 | NR | NR |
| Ramallo-Farina [2020] | NR | NR | NR | NR | NR | NR | NR | NR | NR | NR | NR | NR | NR | NR | NR | NR | NR | NR |
| Shahid  [2014] | NR | NR | NR | NR | NR | NR | NR | NR | NR | NR | NR | NR | NR | NR | NR | NR | NR | NR |
| Shea  [2009] | 176 | 169 | NR | NR | NR | NR | NR | NR | NR | NR | NR | NR | NR | NR | 176 | 169 | NR | NR |
| Tang  [2013] | NR | NR | NR | NR | NR | NR | NR | NR | NR | NR | NR | NR | NR | NR | 0 | 0 | NR | NR |
| Varney  [2014] | 2 | 3 | NR | NR | NR | NR | NR | NR | NR | NR | NR | NR | NR | NR | 1 | 2 | NR | NR |
| Vinitha  [2019] | NR | NR | NR | NR | NR | NR | NR | NR | NR | NR | NR | NR | NR | NR | NR | NR | NR | NR |
| Wild  [2016]^b^ | 8 | 6 | 2 | 2 | NR | NR | 2 | 1 | 2 | 2 | 1 | 0 | NR | NR | 1 | 1 | NR | NR |
| Yoo  [2009] | NR | NR | NR | NR | NR | NR | NR | NR | NR | NR | NR | NR | NR | NR | NR | NR | NR | NR |
| Zhou  [2014] | 7 | 16 | NR | NR | NR | NR | NR | NR | 7 | 16 | NR | NR | NR | NR | NR | NR | NR | NR |

**Legend:** Data is presented as raw numbers or mean (standard deviation). ^a^ Reported microvascular disease presence at baseline, but not follow-up. For Eakin et al. (2014), the adverse events reported included 4 participants requiring hospitalization (not described) and events plausibly related to study participation were reported by 17 and 28 participants, but no hypoglycemic events were reported. For Lim et al. (2021), 3 cases of hypoglycemia were reported in the intervention group but none required hospitalization. For Shea et al. (2009), none of the deaths were attributable directly to the intervention. For Wild et al. (2016), adverse events in the intervention group included 2 hypoglycemic episodes, 1 hypotensive episode, 1 case of peripheral angioplasty and 1 case of myocardial infarction, 1 admission to hospital for heart failure and 1 cerebral infarction with hemorrhagic transformation. In the usual care group there were 2 hypoglycemic episodes, 1 abscess in the presence of hyperglycemia, 1 ischemic stroke, 1 coronary angioplasty and 1 hospital admission for urinary tract infection and hyperglycemia. For Zhou et al. (2014), all adverse events were hypoglycemic events. MACE = major cardiovascular adverse events including myocardial infarction and stroke.

# Supplementary Table 10: Data on the secondary outcomes of interest reported in the included randomized controlled trials.

| **Study** | **Cost of intervention** | | **Quality-of-life tool(s) used** | **Baseline quality of life score** | | **Quality-of-life score at follow up** | |
| --- | --- | --- | --- | --- | --- | --- | --- |
|  | Intervention | Control |  | Intervention | Control | Intervention | Control |
| Aytekin Kanadli  [2016] | NR | NR | NR | NR | NR | NR | NR |
| Blackberry [2013] | NR | NR | AQoL | 0.78  (0.18) | 0.78  (0.17) | 0.79  (0.17) | 0.77  (0.18) |
| Bond  [2007] | NR | NR | NR | NR | NR | NR | NR |
| Crowley [2013] | NR | NR | NR | NR | NR | NR | NR |
| Davis  [2010] | NR | NR | NR | NR | NR | NR | NR |
| de Vasconcelos [2018] | NR | NR | NR | NR | NR | NR | NR |
| Eakin  [2014] | NR | NR | NR | NR | NR | NR | NR |
| Harno  [2006] | NR | NR | NR | NR | NR | NR | NR |
| Holbrook  [2009] | NR | NR | SF-12 | NR | NR | NR | NR |
| Huo  [2019] | NR | NR | Seattle Angina Questionnaire | 84.9  (13.3) | 86.2  (11.5) | 87.4  (12.7) | 86.6  (12.3) |
| Kempf  [2017]^a^ | NR | NR | SF-23 | 43 (11)  38 (6) | 41 (12)  38 (5) | 48 (10)*  39 (5) | 43 (12)  38 (6) |
| Krein  [2004] | NR | NR | NR | NR | NR | NR | NR |
| Leichter  [2013] | NR | NR | NR | NR | NR | NR | NR |
| Lim  [2021] | NR | NR | NR | NR | NR | NR | NR |
| Liou  [2014] | NR | NR | NR | NR | NR | NR | NR |
| Nicolucci  [2015]^a^ | NR | NR | SF-36 | 44.3 (10.2)  48.6 (10.0) | 45.3 (8.7)  48.0 (10.1) | 46.9 (8.8)*  50.0 (10.8) | 45.4 (10.2)  46.7 (10.2) |
| Odnoletkova  [2016] | NR | NR | NR | NR | NR | NR | NR |
| Quinn  [2011] | NR | NR | NR | NR | NR | NR | NR |
| Ramallo-Farina [2020] | NR | NR | NR | NR | NR | NR | NR |
| Shahid  [2014] | NR | NR | NR | NR | NR | NR | NR |
| Shea  [2009] | NR | NR | NR | NR | NR | NR | NR |
| Tang  [2013] | NR | NR | NR | NR | NR | NR | NR |
| Varney  [2014] | NR | NR | NR | NR | NR | NR | NR |
| Vinitha  [2019] | NR | NR | EQ-5D | 0.99  (0.02) | 0.99  (0.03) | 0.99  (0.03) | 0.99  (0.03) |
| Wild  [2016]^b^ | NR | NR | EQ-5D | 0.8  (0.3) | 0.8  (0.2) | 0.8  (0.3) | 0.8  (0.3) |
| Yoo  [2009] | NR | NR | NR | NR | NR | NR | NR |
| Zhou  [2014] | NR | NR | NR | NR | NR | NR | NR |

**Legend:** Data is presented as raw numbers or mean (standard deviation). ^a^ For Kempf et al (2017) and Nicolucci et al (2015); quality of life data for the SF-23 and SF-36 respectively is presented as mean (standard deviation) for the physical component, then the mental component. ^b^ Wild et al. (2016) reported the cost of the intervention, which costs significantly more than usual care (mean difference per patient £286.00 [95% CI £154.27 to £409.62]) due to telemonitoring service costs and additional nurse phone consultations. For Eakin et al. (2014), the adverse events reported included 4 participants requiring hospitalisation (not described) and events plausibly related to study participation were reported by 17 and 28 participants, but no hypoglycaemic events were reported. For Lim et al. (2021), 3 cases of hypoglycaemia were reported in the intervention group but none required hospitalisation. For Shea et al. (2009), none of the deaths were attributable directly to the intervention. For Wild et al. (2016), adverse events in the intervention group included 2 hypoglyacemic episodes, 1 hypotensive episode, 1 case of peripheral angioplasty and 1 case of myocardial infarction, 1 admission to hospital for heart failure and 1 cerebral infarction with haemorrhagic transformation. In the usual care group there were 2 hypoglycaemic episodes, 1 abscess in the presence of hyperglycaemia, 1 ischaemic stroke, 1 coronary angioplasty and 1 hospital admission for urinary tract infection and hyperglycaemia. For Zhou et al. (2014) all adverse events were hypoglyacemic events. * Was statistically significant between groups.

# Supplementary Results: Additional analyses, plots, and figures

## 1. Funnel plots for primary outcomes

### 1.1 Impact of remote intervention on glycated haemoglobin A1c (HbA1c)


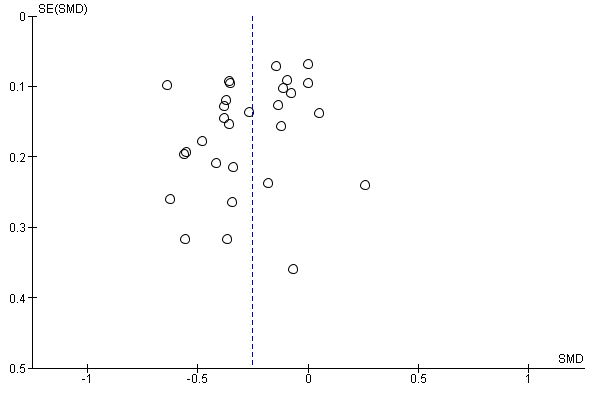


### 1.2 Impact of remote intervention on Total cholesterol (TC)


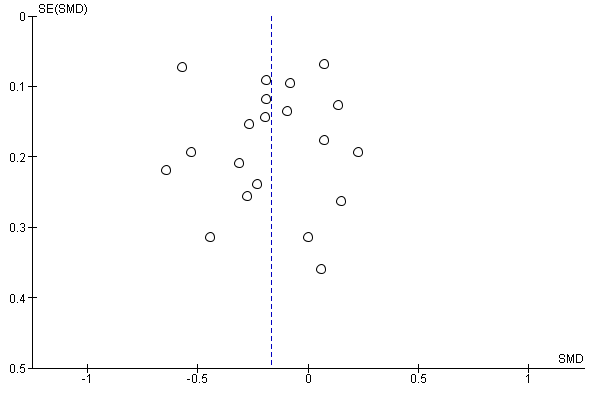


### 1.3 Impact of remote intervention on low-density lipoprotein cholesterol (LDL-c)


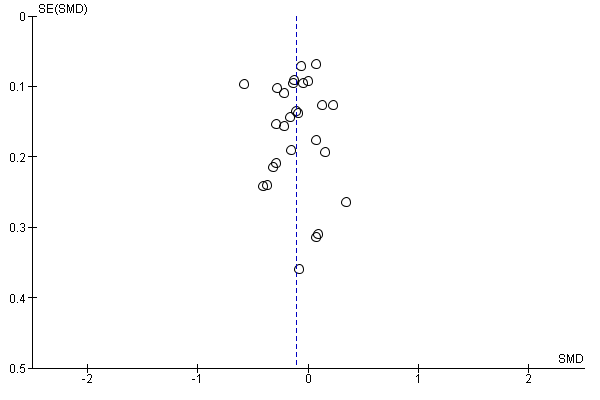


### 1.4 Impact of remote intervention on systolic blood pressure (SBP)


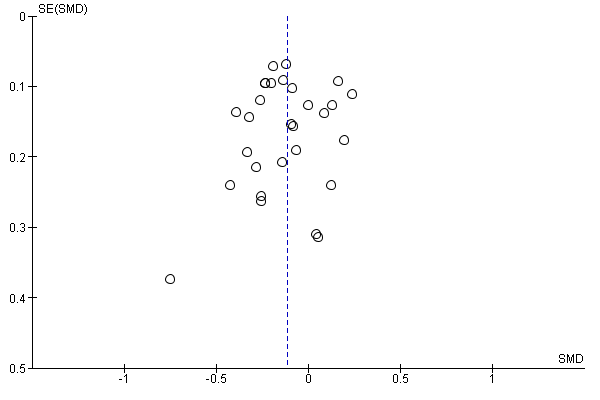


### 1.5 Impact of remote intervention on diastolic blood pressure (DBP)


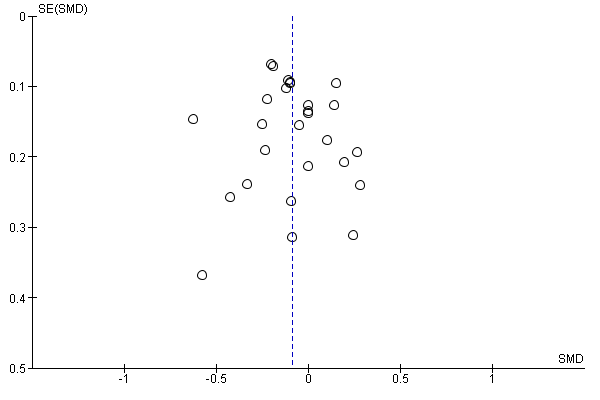


## 2. Leave one out (LOO) sensitivity analyses for primary outcomes

### 2.1 Impact of remote intervention on glycated haemoglobin A1c (HbA1c)

| Study excluded | Effect size estimate SMD  [95% CI] | Heterogeneity | Test for overall effect (Z) |
| --- | --- | --- | --- |
| None | -0.25 [-0.33, -0.17] | Heterogeneity: Tau² = 0.03; Chi² = 70.70, df = 28 (P < 0.0001); I² = 60% | Test for overall effect: Z = 6.17 (P < 0.00001) |
| Aytekin Kanadli 2016 | -0.25 [-0.33, -0.17] | Heterogeneity: Tau² = 0.03; Chi² = 70.39, df = 27 (P < 0.00001); I² = 62% | Test for overall effect: Z = 6.00 (P < 0.00001) |
| Blackberry 2013 | -0.27 [-0.35, -0.18] | Heterogeneity: Tau² = 0.02; Chi² = 65.02, df = 27 (P < 0.0001); I² = 58% | Test for overall effect: Z = 6.41 (P < 0.00001) |
| Bond 2007 | -0.25 [-0.33, -0.17] | Heterogeneity: Tau² = 0.02; Chi² = 68.26, df = 27 (P < 0.0001); I² = 60% | Test for overall effect: Z = 5.99 (P < 0.00001) |
| Crowley 2013 | -0.26 [-0.35, -0.18] | Heterogeneity: Tau² = 0.03; Chi² = 68.92, df = 27 (P < 0.0001); I² = 61% | Test for overall effect: Z = 6.18 (P < 0.00001) |
| Davis 2010 | -0.26 [-0.34, -0.18] | Heterogeneity: Tau² = 0.03; Chi² = 70.30, df = 27 (P < 0.0001); I² = 62% | Test for overall effect: Z = 6.11 (P < 0.00001) |
| de Vasconcelos 2018 | -0.26 [-0.34, -0.17] | Heterogeneity: Tau² = 0.03; Chi² = 70.52, df = 27 (P < 0.00001); I² = 62% | Test for overall effect: Z = 6.15 (P < 0.00001) |
| Eakin 2014 | -0.26 [-0.34, -0.18] | Heterogeneity: Tau² = 0.03; Chi² = 70.25, df = 27 (P < 0.0001); I² = 62% | Test for overall effect: Z = 6.08 (P < 0.00001) |
| Harno 2006 | -0.25 [-0.33, -0.17] | Heterogeneity: Tau² = 0.03; Chi² = 69.87, df = 27 (P < 0.0001); I² = 61% | Test for overall effect: Z = 5.93 (P < 0.00001) |
| Holbrook 2009 | -0.25 [-0.33, -0.17] | Heterogeneity: Tau² = 0.03; Chi² = 68.63, df = 27 (P < 0.0001); I² = 61% | Test for overall effect: Z = 5.84 (P < 0.00001) |
| Huo 2019 | -0.25 [-0.33, -0.17] | Heterogeneity: Tau² = 0.03; Chi² = 68.44, df = 27 (P < 0.0001); I² = 61% | Test for overall effect: Z = 5.83 (P < 0.00001) |
| Kempf 2017 | -0.25 [-0.33, -0.17] | Heterogeneity: Tau² = 0.03; Chi² = 68.54, df = 27 (P < 0.0001); I² = 61% | Test for overall effect: Z = 5.93 (P < 0.00001) |
| Krein 2004 | -0.27 [-0.35, -0.18] | Heterogeneity: Tau² = 0.02; Chi² = 66.81, df = 27 (P < 0.0001); I² = 60% | Test for overall effect: Z = 6.40 (P < 0.00001) |
| Leichter 2013 | -0.26 [-0.34, -0.18] | Heterogeneity: Tau² = 0.02; Chi² = 66.72, df = 27 (P < 0.0001); I² = 60% | Test for overall effect: Z = 6.46 (P < 0.00001) |
| Lim 2021 | -0.25 [-0.33, 0.17] | Heterogeneity: Tau² = 0.03; Chi² = 69.47, df = 27 (P < 0.0001); I² = 61% | Test for overall effect: Z = 5.91 (P < 0.00001) |
| Liou 2014 | -0.25 [-0.33, -0.17] | Heterogeneity: Tau² = 0.03; Chi² = 69.81, df = 27 (P < 0.0001); I² = 61% | Test for overall effect: Z = 5.97 (P < 0.00001) |
| Nicolucci 2015 | -0.25 [-0.33, -0.17] | Heterogeneity: Tau² = 0.03; Chi² = 69.14, df = 27 (P < 0.0001); I² = 61% | Test for overall effect: Z = 5.89 (P < 0.00001) |
| Odnoletkova 2016 | -0.26 [-0.35, -0.18] | Heterogeneity: Tau² = 0.03; Chi² = 68.66, df = 27 (P < 0.0001); I² = 61% | Test for overall effect: Z = 6.14 (P < 0.00001) |
| Quinn 2011 (a b c) | -0.24 [-0.32, -0.15] | Heterogeneity: Tau² = 0.03; Chi² = 66.01, df = 24 (P < 0.00001); I² = 64% | Test for overall effect: Z = 5.53 (P < 0.00001) |
| Quinn 2011a | -0.25 [-0.33, -0.17] | Heterogeneity: Tau² = 0.03; Chi² = 69.58, df = 27 (P < 0.0001); I² = 61% | Test for overall effect: Z = 6.03 (P < 0.00001) |
| Quinn 2011b | -0.25 [-0.33, -0.17] | Heterogeneity: Tau² = 0.03; Chi² = 70.48, df = 27 (P < 0.00001); I² = 62% | Test for overall effect: Z = 6.05 (P < 0.00001) |
| Quinn 2011c | -0.25 [-0.33, -0.17] | Heterogeneity: Tau² = 0.03; Chi² = 70.47, df = 27 (P < 0.00001); I² = 62% | Test for overall effect: Z = 6.03 (P < 0.00001) |
| Ramallo Farina 2020 | -0.27 [-0.35, -0.19] | Heterogeneity: Tau² = 0.02; Chi² = 59.24, df = 27 (P = 0.0003); I² = 54% | Test for overall effect: Z = 6.61 (P < 0.00001) |
| Shahid 2014 | -0.23 [-0.30, -0.15] | Heterogeneity: Tau² = 0.02; Chi² = 51.26, df = 27 (P = 0.003); I² = 47% | Test for overall effect: Z = 6.17 (P < 0.00001) |
| Shea 2009 | -0.26 [-0.35, -0.18] | Heterogeneity: Tau² = 0.03; Chi² = 69.47, df = 27 (P < 0.0001); I² = 61% | Test for overall effect: Z = 5.98 (P < 0.00001) |
| Tang 2013 | -0.26 [-0.34, -0.18] | Heterogeneity: Tau² = 0.03; Chi² = 69.57, df = 27 (P < 0.0001); I² = 61% | Test for overall effect: Z = 6.10 (P < 0.00001) |
| Varney 2014 | -0.26 [-0.34, -0.17] | Heterogeneity: Tau² = 0.03; Chi² = 70.67, df = 27 (P < 0.00001); I² = 62% | Test for overall effect: Z = 6.09 (P < 0.00001) |
| Vinitha 2019 | -0.25 [-0.34, -0.17] | Heterogeneity: Tau² = 0.03; Chi² = 70.58, df = 27 (P < 0.00001); I² = 62% | Test for overall effect: Z = 5.96 (P < 0.00001) |
| Wild 2016 | -0.25 [-0.33, -0.17] | Heterogeneity: Tau² = 0.03; Chi² = 69.09, df = 27 (P < 0.0001); I² = 61% | Test for overall effect: Z = 5.87 (P < 0.00001) |
| Yoo 2009 | -0.25 [-0.33, -0.16] | Heterogeneity: Tau² = 0.02; Chi² = 67.75, df = 27 (P < 0.0001); I² = 60% | Test for overall effect: Z = 5.94 (P < 0.00001) |
| Zhou 2014 | -0.24 [-0.33, -0.16] | Heterogeneity: Tau² = 0.02; Chi² = 67.66, df = 27 (P < 0.0001); I² = 60% | Test for overall effect: Z = 5.94 (P < 0.00001) |

Legend: 95% CI = 95% confidence interval. SMD= standardised mean difference. Quinn et al. had three intervention groups: interventional group a: online coaching only, group b: coaching and primary care providers portal, and group c: coach PCP portal with decision-support.

### 2.2 Impact of remote intervention on total cholesterol (TC)

| Study excluded | Effect size estimate SMD  [95% CI] | Heterogeneity | Test for overall effect (Z) |
| --- | --- | --- | --- |
| None | -0.17 [-0.29, -0.04] | Heterogeneity: Tau² = 0.05; Chi² = 66.96, df = 19 (P < 0.00001); I² = 72% | Test for overall effect: Z = 2.64 (P = 0.008) |
| Aytekin Kanadli 2016 | -0.15 [-0.27, -0.02] | Heterogeneity: Tau² = 0.05; Chi² = 62.25, df = 18 (P < 0.00001); I² = 71% | Test for overall effect: Z = 2.31 (P = 0.02) |
| Blackberry 2013 | -0.17 [-0.31, -0.04] | Heterogeneity: Tau² = 0.05; Chi² = 65.93, df = 18 (P < 0.00001); I² = 73% | Test for overall effect: Z = 2.54 (P = 0.01) |
| Bond 2007 | -0.16 [-0.29, -0.04] | Heterogeneity: Tau² = 0.05; Chi² = 66.80, df = 18 (P < 0.00001); I² = 73% | Test for overall effect: Z = 2.50 (P = 0.01) |
| de Vasconcelos 2018 | -0.17 [-0.30, -0.05] | Heterogeneity: Tau² = 0.05; Chi² = 66.55, df = 18 (P < 0.00001); I² = 73% | Test for overall effect: Z = 2.67 (P = 0.008) |
| Harno 2006 | -0.16 [-0.29, -0.03] | Heterogeneity: Tau² = 0.05; Chi² = 66.56, df = 18 (P < 0.00001); I² = 73% | Test for overall effect: Z = 2.42 (P = 0.02) |
| Kempf 2017 | -0.18 [-0.31, -0.05] | Heterogeneity: Tau² = 0.05; Chi² = 64.98, df = 18 (P < 0.00001); I² = 72% | Test for overall effect: Z = 2.75 (P = 0.006) |
| Lim 2021 | -0.16 [-0.30, -0.03] | Heterogeneity: Tau² = 0.05; Chi² = 66.94, df = 18 (P < 0.00001); I² = 73% | Test for overall effect: Z = 2.47 (P = 0.01) |
| Liou 2014 | -0.16 [-0.29, -0.03] | Heterogeneity: Tau² = 0.05; Chi² = 66.50, df = 18 (P < 0.00001); I² = 73% | Test for overall effect: Z = 2.45 (P = 0.01) |
| Nicolucci 2015 | -0.19 [-0.31, -0.06] | Heterogeneity: Tau² = 0.05; Chi² = 60.85, df = 18 (P < 0.00001); I² = 70% | Test for overall effect: Z = 2.90 (P = 0.004) |
| Odnoletkova 2016 | -0.16 [-0.30, -0.03] | Heterogeneity: Tau² = 0.06; Chi² = 66.91, df = 18 (P < 0.00001); I² = 73% | Test for overall effect: Z = 2.40 (P = 0.02) |
| Quinn 2011 (a b c) | -0.17 [-0.31, -0.04] | Heterogeneity: Tau² = 0.05; Chi² = 64.42, df = 16 (P < 0.00001); I² = 75% | Test for overall effect: Z = 2.60 (P = 0.009) |
| Quinn 2011a | -0.16 [-0.28, -0.03] | Heterogeneity: Tau² = 0.05; Chi² = 66.21, df = 18 (P < 0.00001); I² = 73% | Test for overall effect: Z = 2.47 (P = 0.01) |
| Quinn 2011b | -0.17 [-0.30, -0.04] | Heterogeneity: Tau² = 0.05; Chi² = 66.66, df = 18 (P < 0.00001); I² = 73% | Test for overall effect: Z = 2.65 (P = 0.008) |
| Quinn 2011c | -0.18 [-0.30, -0.05] | Heterogeneity: Tau² = 0.05; Chi² = 65.47, df = 18 (P < 0.00001); I² = 73% | Test for overall effect: Z = 2.76 (P = 0.006) |
| Ramallo Farina 2020 | -0.19 [-0.31, -0.06] | Heterogeneity: Tau² = 0.04; Chi² = 51.90, df = 18 (P < 0.0001); I² = 65% | Test for overall effect: Z = 2.97 (P = 0.003) |
| Shea 2009 | -0.12 [-0.22, -0.03] | Heterogeneity: Tau² = 0.02; Chi² = 31.70, df = 18 (P = 0.02); I² = 43% | Test for overall effect: Z = 2.52 (P = 0.01) |
| Varney 2014 | -0.16 [-0.29, -0.04] | Heterogeneity: Tau² = 0.05; Chi² = 66.90, df = 18 (P < 0.00001); I² = 73% | Test for overall effect: Z = 2.51 (P = 0.01) |
| Vinitha 2019 | -0.17 [-0.30, -0.04] | Heterogeneity: Tau² = 0.05; Chi² = 66.62, df = 18 (P < 0.00001); I² = 73% | Test for overall effect: Z = 2.57 (P = 0.01) |
| Wild 2016 | -0.16 [-0.30, -0.03] | Heterogeneity: Tau² = 0.05; Chi² = 66.94, df = 18 (P < 0.00001); I² = 73% | Test for overall effect: Z = 2.45 (P = 0.01) |
| Yoo 2009 | -0.15 [-0.27, -0.02] | Heterogeneity: Tau² = 0.05; Chi² = 63.46, df = 18 (P < 0.00001); I² = 72% | Test for overall effect: Z = 2.32 (P = 0.02) |
| Zhou 2014 | -0.19 [-0.31, -0.06] | Heterogeneity: Tau² = 0.05; Chi² = 62.61, df = 18 (P < 0.00001); I² = 71% | Test for overall effect: Z = 2.91 (P = 0.004) |

Legend: 95% CI = 95% confidence interval. SMD= standardised mean difference. Quinn et al. had three intervention groups: interventional group a: online coaching only, group b: coaching and primary care providers portal, and group c: coach PCP portal with decision-support.

### 2.3 Impact of remote intervention on low-density lipoprotein cholesterol (LDL-c)

| Study excluded | Effect size estimate SMD  [95% CI] | Heterogeneity | Test for overall effect (Z) |
| --- | --- | --- | --- |
| None | -0.11 [-0.19, -0.03] | Heterogeneity: Tau² = 0.02; Chi² = 58.88, df = 26 (P = 0.0002); I² = 56% | Test for overall effect: Z = 2.78 (P = 0.006) |
| Aytekin Kanadli 2016 | -0.11 [-0.18, -0.03] | Heterogeneity: Tau² = 0.02; Chi² = 57.85, df = 25 (P = 0.0002); I² = 57% | Test for overall effect: Z = 2.61 (P = 0.009) |
| Blackberry 2013 | -0.11 [-0.20, -0.03] | Heterogeneity: Tau² = 0.02; Chi² = 58.50, df = 25 (P = 0.0002); I² = 57% | Test for overall effect: Z = 2.72 (P = 0.007) |
| Crowley 2013 | -0.11 [-0.19, -0.02] | Heterogeneity: Tau² = 0.02; Chi² = 57.75, df = 25 (P = 0.0002); I² = 57% | Test for overall effect: Z = 2.54 (P = 0.01) |
| Davis 2010 | -0.11 [-0.19, -0.03] | Heterogeneity: Tau² = 0.02; Chi² = 58.34, df = 25 (P = 0.0002); I² = 57% | Test for overall effect: Z = 2.60 (P = 0.009) |
| de Vasconcelos 2018 | -0.11 [-0.19, -0.03] | Heterogeneity: Tau² = 0.02; Chi² = 58.87, df = 25 (P = 0.0001); I² = 58% | Test for overall effect: Z = 2.74 (P = 0.006) |
| Eakin 2014 | -0.13 [-0.20, -0.05] | Heterogeneity: Tau² = 0.02; Chi² = 52.00, df = 25 (P = 0.001); I² = 52% | Test for overall effect: Z = 3.22 (P = 0.001) |
| Harno 2006 | -0.10 [-0.18, -0.02] | Heterogeneity: Tau² = 0.02; Chi² = 57.38, df = 25 (P = 0.0002); I² = 56% | Test for overall effect: Z = 2.55 (P = 0.01) |
| Holbrook 2009 | -0.11 [-0.19, -0.03] | Heterogeneity: Tau² = 0.02; Chi² = 58.73, df = 25 (P = 0.0002); I² = 57% | Test for overall effect: Z = 2.59 (P = 0.010) |
| Huo 2019 | -0.12 [-0.20, -0.04] | Heterogeneity: Tau² = 0.02; Chi² = 57.54, df = 25 (P = 0.0002); I² = 57% | Test for overall effect: Z = 2.80 (P = 0.005) |
| Kempf 2017 | -0.12 [-0.20, -0.04] | Heterogeneity: Tau² = 0.02; Chi² = 57.84, df = 25 (P = 0.0002); I² = 57% | Test for overall effect: Z = 2.86 (P = 0.004) |
| Krein 2004 | -0.11 [-0.19, -0.03] | Heterogeneity: Tau² = 0.02; Chi² = 58.87, df = 25 (P = 0.0001); I² = 58% | Test for overall effect: Z = 2.69 (P = 0.007) |
| Leichter 2013 | -0.10 [-0.18, -0.03] | Heterogeneity: Tau² = 0.02; Chi² = 57.32, df = 25 (P = 0.0002); I² = 56% | Test for overall effect: Z = 2.60 (P = 0.009) |
| Lim 2021 | -0.11 [-0.19, -0.03] | Heterogeneity: Tau² = 0.02; Chi² = 58.70, df = 25 (P = 0.0002); I² = 57% | Test for overall effect: Z = 2.63 (P = 0.009) |
| Liou 2014 | -0.11 [-0.19, -0.03] | Heterogeneity: Tau² = 0.02; Chi² = 58.03, df = 25 (P = 0.0002); I² = 57% | Test for overall effect: Z = 2.61 (P = 0.009) |
| Nicolucci 2015 | -0.12 [-0.20, -0.04] | Heterogeneity: Tau² = 0.02; Chi² = 55.61, df = 25 (P = 0.0004); I² = 55% | Test for overall effect: Z = 3.00 (P = 0.003) |
| Odnoletkova 2016 | -0.11 [-0.19, -0.03] | Heterogeneity: Tau² = 0.02; Chi² = 58.78, df = 25 (P = 0.0002); I² = 57% | Test for overall effect: Z = 2.59 (P = 0.010) |
| Quinn 2011 (a b c) | -0.12 [-0.20, -0.04] | Heterogeneity: Tau² = 0.02; Chi² = 55.26, df = 23 (P = 0.0002); I² = 58% | Test for overall effect: Z = 3.05 (P = 0.002) |
| Quinn 2011a | -0.11 [-0.19, -0.03] | Heterogeneity: Tau² = 0.02; Chi² = 58.48, df = 25 (P = 0.0002); I² = 57% | Test for overall effect: Z = 2.81 (P = 0.005) |
| Quinn 2011b | -0.11 [-0.19, -0.03] | Heterogeneity: Tau² = 0.02; Chi² = 58.56, df = 25 (P = 0.0002); I² = 57% | Test for overall effect: Z = 2.80 (P = 0.005) |
| Quinn 2011c | -0.12 [-0.20, -0.04] | Heterogeneity: Tau² = 0.02; Chi² = 56.01, df = 25 (P = 0.0004); I² = 55% | Test for overall effect: Z = 2.99 (P = 0.003) |
| Ramallo Farina 2020 | -0.12 [-0.20, -0.04] | Heterogeneity: Tau² = 0.02; Chi² = 51.83, df = 25 (P = 0.001); I² = 52% | Test for overall effect: Z = 3.04 (P = 0.002) |
| Shahid 2014 | -0.08 [-0.14, -0.02] | Heterogeneity: Tau² = 0.01; Chi² = 33.72, df = 25 (P = 0.11); I² = 26% | Test for overall effect: Z = 2.57 (P = 0.01) |
| Shea 2009 | -0.11 [-0.20, -0.03] | Heterogeneity: Tau² = 0.02; Chi² = 58.59, df = 25 (P = 0.0002); I² = 57% | Test for overall effect: Z = 2.65 (P = 0.008) |
| Tang 2013 | -0.10 [-0.18, -0.02] | Heterogeneity: Tau² = 0.02; Chi² = 55.77, df = 25 (P = 0.0004); I² = 55% | Test for overall effect: Z = 2.49 (P = 0.01) |
| Varney 2014 | -0.11 [-0.18, -0.03] | Heterogeneity: Tau² = 0.02; Chi² = 57.62, df = 25 (P = 0.0002); I² = 57% | Test for overall effect: Z = 2.61 (P = 0.009) |
| Vinitha 2019 | -0.11 [-0.19, -0.03] | Heterogeneity: Tau² = 0.02; Chi² = 58.87, df = 25 (P = 0.0001); I² = 58% | Test for overall effect: Z = 2.67 (P = 0.008) |
| Yoo 2009 | -0.11 [-0.19, -0.03] | Heterogeneity: Tau² = 0.02; Chi² = 58.81, df = 25 (P = 0.0002); I² = 57% | Test for overall effect: Z = 2.67 (P = 0.008) |
| Zhou 2014 | -0.12 [-0.20, -0.04] | Heterogeneity: Tau² = 0.02; Chi² = 57.08, df = 25 (P = 0.0003); I² = 56% | Test for overall effect: Z = 2.93 (P = 0.003) |

Legend: 95% CI = 95% confidence interval. SMD= standardised mean difference. Quinn et al. (2011) had three intervention groups: intervention group a: online coaching only, group b: coaching and primary care providers portal, and group c: coach PCP portal with decision-support.

### 2.4 Impact of remote intervention on systolic blood pressure (SBP)

| Study excluded | Effect size estimate SMD  [95% CI] | Heterogeneity | Test for overall effect (Z) |
| --- | --- | --- | --- |
| None | -0.11 [-0.18, -0.04] | Heterogeneity: Tau² = 0.01; Chi² = 50.35, df = 28 (P = 0.006); I² = 44% | Test for overall effect: Z = 3.25 (P = 0.001) |
| Aytekin Kanadli 2016 | -0.11 [-0.18, -0.04] | Heterogeneity: Tau² = 0.01; Chi² = 49.71, df = 27 (P = 0.005); I² = 46% | Test for overall effect: Z = 3.09 (P = 0.002) |
| Blackberry 2013 | -0.11 [-0.18, -0.04] | Heterogeneity: Tau² = 0.01; Chi² = 49.47, df = 27 (P = 0.005); I² = 45% | Test for overall effect: Z = 2.98 (P = 0.003) |
| Bond 2007 | -0.10 [-0.18, -0.03] | Heterogeneity: Tau² = 0.01; Chi² = 50.04, df = 27 (P = 0.005); I² = 46% | Test for overall effect: Z = 3.13 (P = 0.002) |
| Crowley 2013 | -0.13 [-0.19, -0.07] | Heterogeneity: Tau² = 0.01; Chi² = 39.74, df = 27 (P = 0.05); I² = 32% | Test for overall effect: Z = 4.06 (P < 0.0001) |
| Davis 2010 | -0.11 [-0.18, -0.04] | Heterogeneity: Tau² = 0.01; Chi² = 50.32, df = 27 (P = 0.004); I² = 46% | Test for overall effect: Z = 3.17 (P = 0.002) |
| de Vasconcelos 2018 | -0.11 [-0.17, -0.04] | Heterogeneity: Tau² = 0.01; Chi² = 47.43, df = 27 (P = 0.009); I² = 43% | Test for overall effect: Z = 3.15 (P = 0.002) |
| Eakin 2014 | -0.12 [-0.19, -0.05] | Heterogeneity: Tau² = 0.01; Chi² = 46.63, df = 27 (P = 0.01); I² = 42% | Test for overall effect: Z = 3.54 (P = 0.0004) |
| Harno 2006 | -0.11 [-0.18, -0.04] | Heterogeneity: Tau² = 0.01; Chi² = 50.34, df = 27 (P = 0.004); I² = 46% | Test for overall effect: Z = 3.16 (P = 0.002) |
| Holbrook 2009 | -0.11 [-0.17, -0.04] | Heterogeneity: Tau² = 0.01; Chi² = 48.59, df = 27 (P = 0.007); I² = 44% | Test for overall effect: Z = 2.95 (P = 0.003) |
| Huo 2019 | -0.13 [-0.19, -0.06] | Heterogeneity: Tau² = 0.01; Chi² = 41.00, df = 27 (P = 0.04); I² = 34% | Test for overall effect: Z = 3.93 (P < 0.0001) |
| Kempf 2017 | -0.12 [-0.19, -0.05] | Heterogeneity: Tau² = 0.01; Chi² = 47.21, df = 27 (P = 0.009); I² = 43% | Test for overall effect: Z = 3.51 (P = 0.0005) |
| Krein 2004 | -0.12 [-0.19, -0.05] | Heterogeneity: Tau² = 0.01; Chi² = 48.24, df = 27 (P = 0.007); I² = 44% | Test for overall effect: Z = 3.42 (P = 0.0006) |
| Leichter 2013 | -0.12 [-0.18, -0.05] | Heterogeneity: Tau² = 0.01; Chi² = 49.38, df = 27 (P = 0.005); I² = 45% | Test for overall effect: Z = 3.33 (P = 0.0009) |
| Lim 2021 | -0.10 [-0.17, -0.04] | Heterogeneity: Tau² = 0.01; Chi² = 48.26, df = 27 (P = 0.007); I² = 44% | Test for overall effect: Z = 2.99 (P = 0.003) |
| Liou 2014 | -0.11 [-0.18, -0.04] | Heterogeneity: Tau² = 0.01; Chi² = 50.33, df = 27 (P = 0.004); I² = 46% | Test for overall effect: Z = 3.15 (P = 0.002) |
| Nicolucci 2015 | -0.12 [-0.19, -0.05] | Heterogeneity: Tau² = 0.01; Chi² = 49.55, df = 27 (P = 0.005); I² = 46% | Test for overall effect: Z = 3.28 (P = 0.001) |
| Odnoletkova 2016 | -0.11 [-0.18, -0.04] | Heterogeneity: Tau² = 0.01; Chi² = 50.27, df = 27 (P = 0.004); I² = 46% | Test for overall effect: Z = 3.04 (P = 0.002) |
| Quinn 2011 (a b c) | -0.11 [-0.18, -0.04] | Heterogeneity: Tau² = 0.01; Chi² = 49.52, df = 25 (P = 0.002); I² = 50% | Test for overall effect: Z = 3.15 (P = 0.002) |
| Quinn 2011a | -0.11 [-0.18, -0.05] | Heterogeneity: Tau² = 0.01; Chi² = 50.10, df = 27 (P = 0.004); I² = 46% | Test for overall effect: Z = 3.25 (P = 0.001) |
| Quinn 2011b | -0.11 [-0.18, -0.05] | Heterogeneity: Tau² = 0.01; Chi² = 50.07, df = 27 (P = 0.004); I² = 46% | Test for overall effect: Z = 3.26 (P = 0.001) |
| Quinn 2011c | -0.11 [-0.18, -0.04] | Heterogeneity: Tau² = 0.01; Chi² = 50.05, df = 27 (P = 0.004); I² = 46% | Test for overall effect: Z = 3.14 (P = 0.002) |
| Ramallo Farina 2020 | -0.11 [-0.18, -0.04] | Heterogeneity: Tau² = 0.02; Chi² = 50.34, df = 27 (P = 0.004); I² = 46% | Test for overall effect: Z = 3.01 (P = 0.003) |
| Shahid 2014 | -0.11 [-0.17, -0.04] | Heterogeneity: Tau² = 0.01; Chi² = 48.61, df = 27 (P = 0.007); I² = 44% | Test for overall effect: Z = 2.95 (P = 0.003) |
| Shea 2009 | -0.11 [-0.18, -0.04] | Heterogeneity: Tau² = 0.01; Chi² = 49.10, df = 27 (P = 0.006); I² = 45% | Test for overall effect: Z = 2.93 (P = 0.003) |
| Tang 2013 | -0.11 [-0.18, -0.04] | Heterogeneity: Tau² = 0.01; Chi² = 50.29, df = 27 (P = 0.004); I² = 46% | Test for overall effect: Z = 3.13 (P = 0.002) |
| Varney 2014 | -0.11 [-0.17, -0.04] | Heterogeneity: Tau² = 0.01; Chi² = 48.65, df = 27 (P = 0.006); I² = 45% | Test for overall effect: Z = 3.08 (P = 0.002) |
| Vinitha 2019 | -0.10 [-0.17, -0.03] | Heterogeneity: Tau² = 0.01; Chi² = 46.01, df = 27 (P = 0.01); I² = 41% | Test for overall effect: Z = 2.96 (P = 0.003) |
| Wild 2016 | -0.11 [-0.17, -0.04] | Heterogeneity: Tau² = 0.01; Chi² = 48.75, df = 27 (P = 0.006); I² = 45% | Test for overall effect: Z = 2.98 (P = 0.003) |
| Yoo 2009 | -0.11 [-0.18, -0.04] | Heterogeneity: Tau² = 0.01; Chi² = 50.30, df = 27 (P = 0.004); I² = 46% | Test for overall effect: Z = 3.20 (P = 0.001) |
| Zhou 2014 | -0.11 [-0.17, -0.04] | Heterogeneity: Tau² = 0.01; Chi² = 49.06, df = 27 (P = 0.006); I² = 45% | Test for overall effect: Z = 3.06 (P = 0.002) |

Legend: 95% CI = 95% confidence interval. SMD= standardised mean difference. Quinn et al. (2011) had three intervention groups: intervention group a: online coaching only, group b: coaching and primary care providers portal, and group c: coach PCP portal with decision-support.

### 2.5 Impact of remote intervention on diastolic blood pressure (DBP)

| Study excluded | Effect size estimate SMD  [95% CI] | Heterogeneity | Test for overall effect (Z) |
| --- | --- | --- | --- |
| None | -0.09 [-0.16, -0.02] | Heterogeneity: Tau² = 0.01; Chi² = 46.29, df = 26 (P = 0.008); I² = 44% | Test for overall effect: Z = 2.38 (P = 0.02) |
| Aytekin Kanadli 2016 | -0.09 [-0.16, -0.02] | Heterogeneity: Tau² = 0.01; Chi² = 46.07, df = 25 (P = 0.006); I² = 46% | Test for overall effect: Z = 2.37 (P = 0.02) |
| Blackberry 2013 | -0.09 [-0.16, -0.01] | Heterogeneity: Tau² = 0.02; Chi² = 46.29, df = 25 (P = 0.006); I² = 46% | Test for overall effect: Z = 2.20 (P = 0.03) |
| Bond 2007 | -0.08 [-0.15, -0.01] | Heterogeneity: Tau² = 0.01; Chi² = 44.68, df = 25 (P = 0.009); I² = 44% | Test for overall effect: Z = 2.21 (P = 0.03) |
| Davis 2010 | -0.09 [-0.16, -0.01] | Heterogeneity: Tau² = 0.01; Chi² = 46.19, df = 25 (P = 0.006); I² = 46% | Test for overall effect: Z = 2.32 (P = 0.02) |
| de Vasconcelos 2018 | -0.08 [-0.15, -0.01] | Heterogeneity: Tau² = 0.01; Chi² = 44.62, df = 25 (P = 0.009); I² = 44% | Test for overall effect: Z = 2.26 (P = 0.02) |
| Eakin 2014 | -0.09 [-0.17, -0.02] | Heterogeneity: Tau² = 0.01; Chi² = 45.66, df = 25 (P = 0.007); I² = 45% | Test for overall effect: Z = 2.39 (P = 0.02) |
| Harno 2006 | -0.08 [-0.15, -0.01] | Heterogeneity: Tau² = 0.01; Chi² = 45.28, df = 25 (P = 0.008); I² = 45% | Test for overall effect: Z = 2.15 (P = 0.03) |
| Holbrook 2009 | -0.09 [-0.16, -0.01] | Heterogeneity: Tau² = 0.02; Chi² = 46.29, df = 25 (P = 0.006); I² = 46% | Test for overall effect: Z = 2.21 (P = 0.03) |
| Kempf 2017 | -0.09 [-0.17, -0.02] | Heterogeneity: Tau² = 0.01; Chi² = 44.94, df = 25 (P = 0.008); I² = 44% | Test for overall effect: Z = 2.49 (P = 0.01) |
| Krein 2004 | -0.09 [-0.16, -0.02] | Heterogeneity: Tau² = 0.01; Chi² = 45.77, df = 25 (P = 0.007); I² = 45% | Test for overall effect: Z = 2.38 (P = 0.02) |
| Leichter 2013 | -0.09 [-0.17, -0.02] | Heterogeneity: Tau² = 0.01; Chi² = 43.75, df = 25 (P = 0.01); I² = 43% | Test for overall effect: Z = 2.59 (P = 0.01) |
| Lim 2021 | -0.07 [-0.13, -0.01] | Heterogeneity: Tau² = 0.01; Chi² = 33.18, df = 25 (P = 0.13); I² = 25% | Test for overall effect: Z = 2.27 (P = 0.02) |
| Liou 2014 | -0.09 [-0.17, -0.02] | Heterogeneity: Tau² = 0.01; Chi² = 44.27, df = 25 (P = 0.01); I² = 44% | Test for overall effect: Z = 2.55 (P = 0.01) |
| Nicolucci 2015 | -0.10 [-0.17, -0.03] | Heterogeneity: Tau² = 0.01; Chi² = 42.56, df = 25 (P = 0.02); I² = 41% | Test for overall effect: Z = 2.68 (P = 0.007) |
| Odnoletkova 2016 | -0.08 [-0.16, -0.01] | Heterogeneity: Tau² = 0.02; Chi² = 46.27, df = 25 (P = 0.006); I² = 46% | Test for overall effect: Z = 2.18 (P = 0.03) |
| Quinn 2011 (a b c) | -0.09 [-0.17, -0.02] | Heterogeneity: Tau² = 0.02; Chi² = 45.05, df = 23 (P = 0.004); I² = 49% | Test for overall effect: Z = 2.36 (P = 0.02) |
| Quinn 2011a | -0.09 [-0.16, -0.02] | Heterogeneity: Tau² = 0.01; Chi² = 45.05, df = 25 (P = 0.008); I² = 45% | Test for overall effect: Z = 2.47 (P = 0.01) |
| Quinn 2011b | -0.09 [-0.16, -0.01] | Heterogeneity: Tau² = 0.01; Chi² = 46.29, df = 25 (P = 0.006); I² = 46% | Test for overall effect: Z = 2.32 (P = 0.02) |
| Quinn 2011c | -0.09 [-0.16, -0.01] | Heterogeneity: Tau² = 0.01; Chi² = 46.29, df = 25 (P = 0.006); I² = 46% | Test for overall effect: Z = 2.31 (P = 0.02) |
| Ramallo Farina 2020 | -0.08 [-0.15, -0.00] | Heterogeneity: Tau² = 0.01; Chi² = 43.85, df = 25 (P = 0.01); I² = 43% | Test for overall effect: Z = 2.02 (P = 0.04) |
| Shahid 2014 | -0.10 [-0.17, -0.03] | Heterogeneity: Tau² = 0.01; Chi² = 38.82, df = 25 (P = 0.04); I² = 36% | Test for overall effect: Z = 2.95 (P = 0.003) |
| Shea 2009 | -0.08 [-0.15, -0.00] | Heterogeneity: Tau² = 0.01; Chi² = 44.53, df = 25 (P = 0.009); I² = 44% | Test for overall effect: Z = 2.04 (P = 0.04) |
| Tang 2013 | -0.08 [-0.16, -0.01] | Heterogeneity: Tau² = 0.02; Chi² = 46.26, df = 25 (P = 0.006); I² = 46% | Test for overall effect: Z = 2.19 (P = 0.03) |
| Varney 2014 | -0.08 [-0.15, -0.01] | Heterogeneity: Tau² = 0.01; Chi² = 45.35, df = 25 (P = 0.008); I² = 45% | Test for overall effect: Z = 2.22 (P = 0.03) |
| Vinitha 2019 | -0.09 [-0.16, -0.02] | Heterogeneity: Tau² = 0.01; Chi² = 45.74, df = 25 (P = 0.007); I² = 45% | Test for overall effect: Z = 2.39 (P = 0.02) |
| Wild 2016 | -0.08 [-0.15, -0.01] | Heterogeneity: Tau² = 0.01; Chi² = 45.17, df = 25 (P = 0.008); I² = 45% | Test for overall effect: Z = 2.11 (P = 0.03) |
| Yoo 2009 | -0.08 [-0.16, -0.01] | Heterogeneity: Tau² = 0.01; Chi² = 45.79, df = 25 (P = 0.007); I² = 45% | Test for overall effect: Z = 2.21 (P = 0.03) |
| Zhou 2014 | -0.10 [-0.17, -0.03] | Heterogeneity: Tau² = 0.01; Chi² = 42.70, df = 25 (P = 0.02); I² = 41% | Test for overall effect: Z = 2.68 (P = 0.007) |

Legend: 95% CI = 95% confidence interval. SMD= standardised mean difference. Quinn et al. (2011) had three intervention groups: intervention group a: online coaching only, group b: coaching and primary care providers portal, and group c: coach PCP portal with decision-support.

## 3. Sensitivity analyses for primary outcomes with studies at high risk of bias excluded

### 3.1 Forest Plot showing the impact of remote intervention on glycated haemoglobin A1c (excluding studies at high risk of bias)


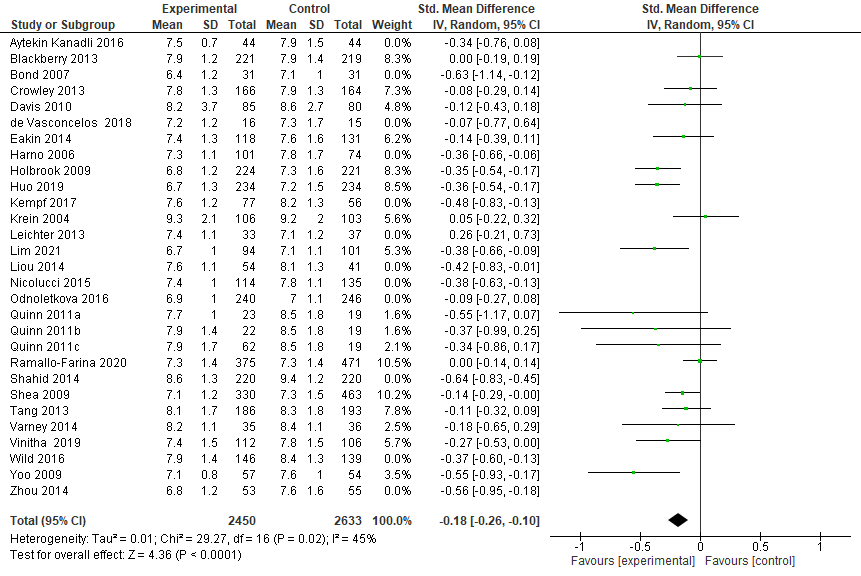


### 3.2 Funnel plot showing the impact of remote intervention on glycated haemoglobin A1c (excluding studies at high risk of bias)


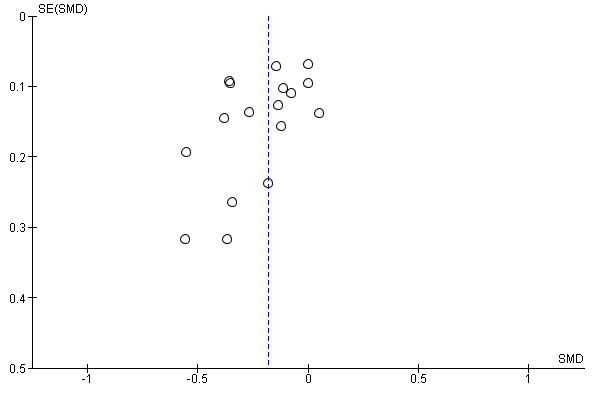


### 3.3 Forest Plot showing the impact of remote intervention on total cholesterol (excluding studies at high risk of bias)


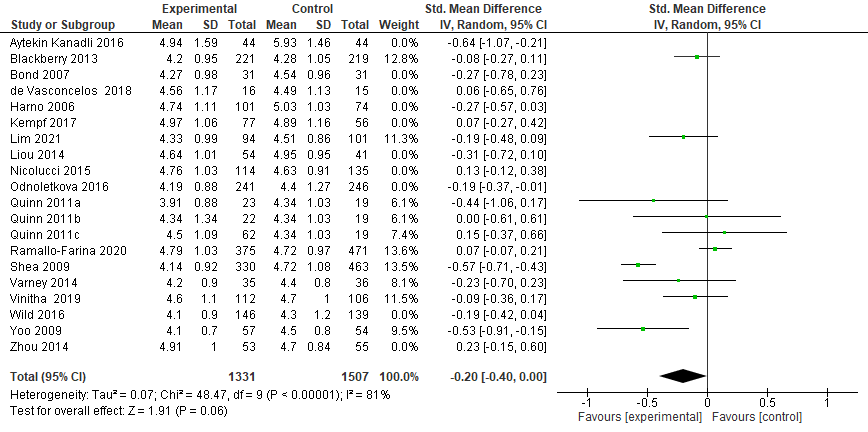


### 3.4 Funnel plot showing the impact of remote intervention on total cholesterol (excluding studies at high risk of bias)


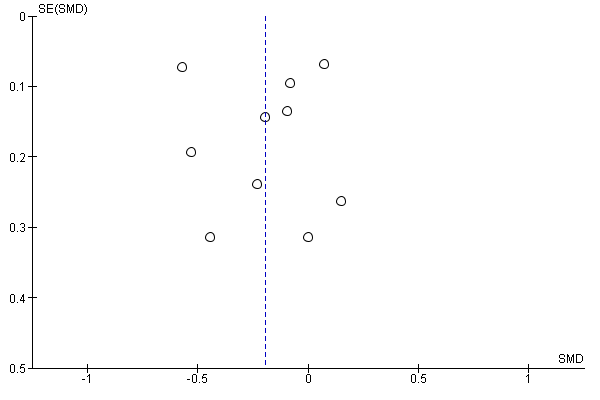


### 3.5 Forest Plot showing the impact of remote intervention on LDL-c (excluding studies at high risk of bias)


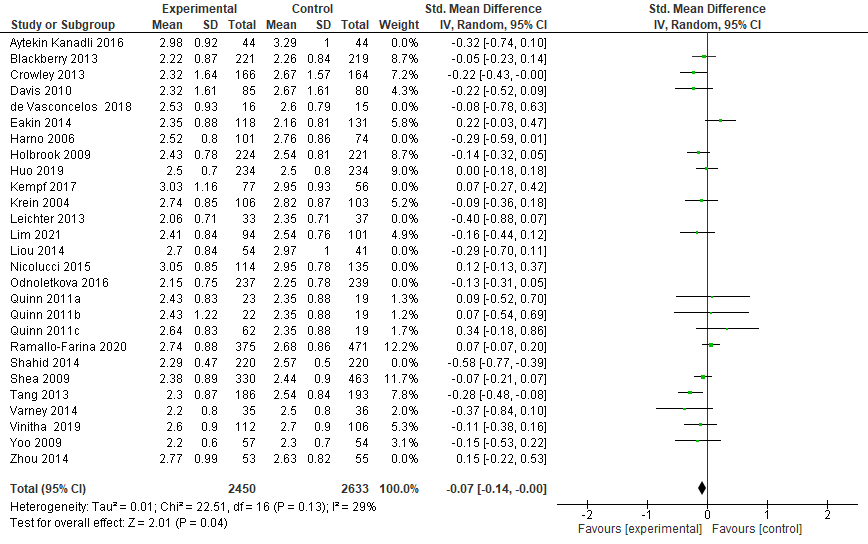


### 3.6 Funnel plot showing the impact of remote intervention on LDL-c (excluding studies at high risk of bias)


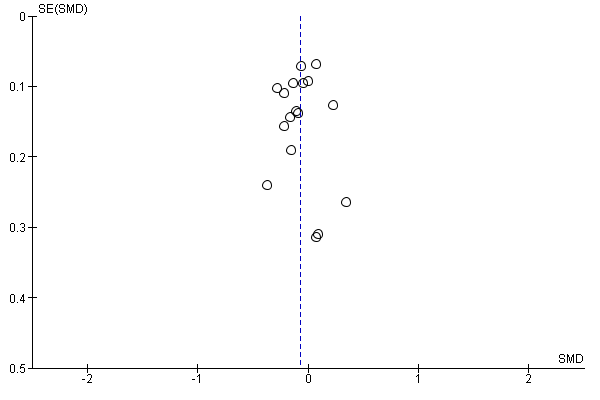


### 3.7 Forest Plot showing the impact of remote intervention on SBP (excluding studies at high risk of bias)


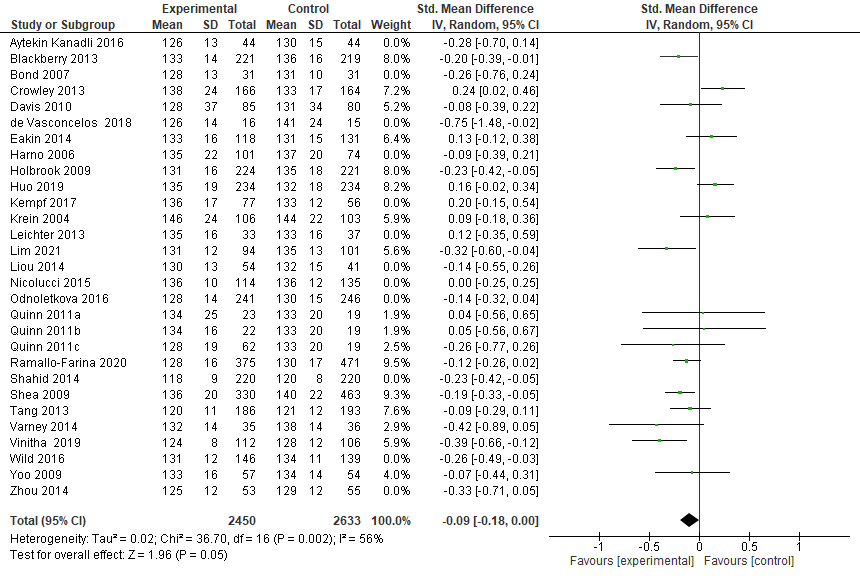


### 3.8 Funnel plot showing the impact of remote intervention on SBP (excluding studies at high risk of bias)


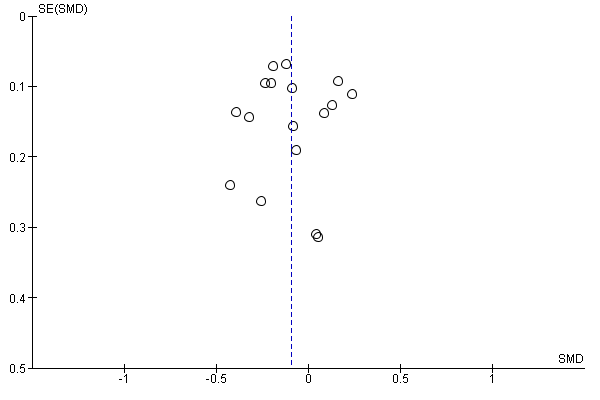


### 3.9 Forest Plot showing the impact of remote intervention on DBP (excluding studies at high risk of bias)


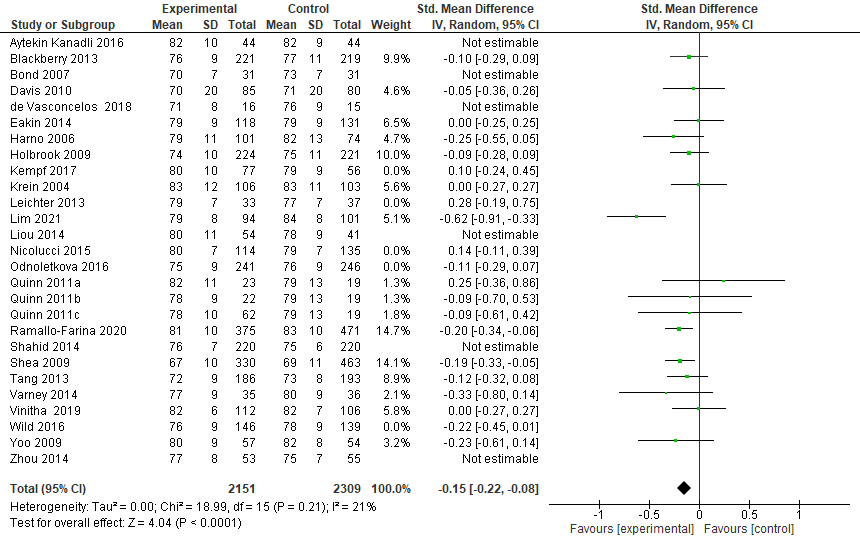


### 3.10 Funnel plot showing the impact of remote intervention on DBP (excluding studies at high risk of bias)


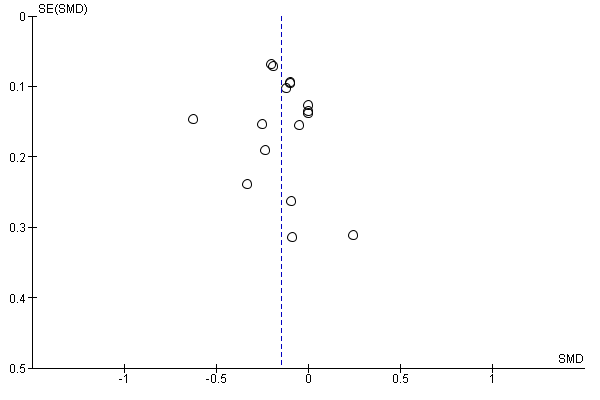


## 4. Subgroup analyses of primary outcomes based on specific remote intervention components

### 4.1 Forest Plot showing the impact of remote interventions with remote patient education on glycated haemoglobin A1c


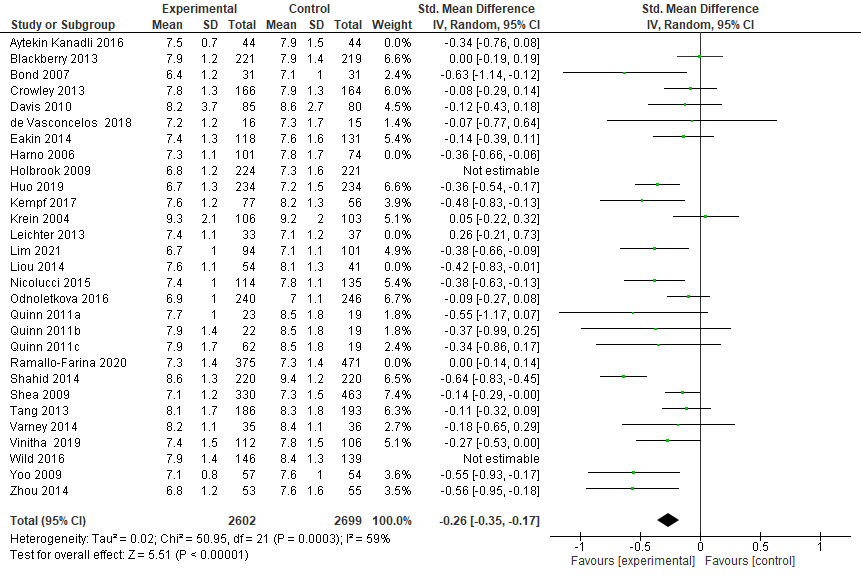


### 4.2 Forest Plot showing the Impact of remote interventions with remote monitoring of risk factors on glycated haemoglobin A1c


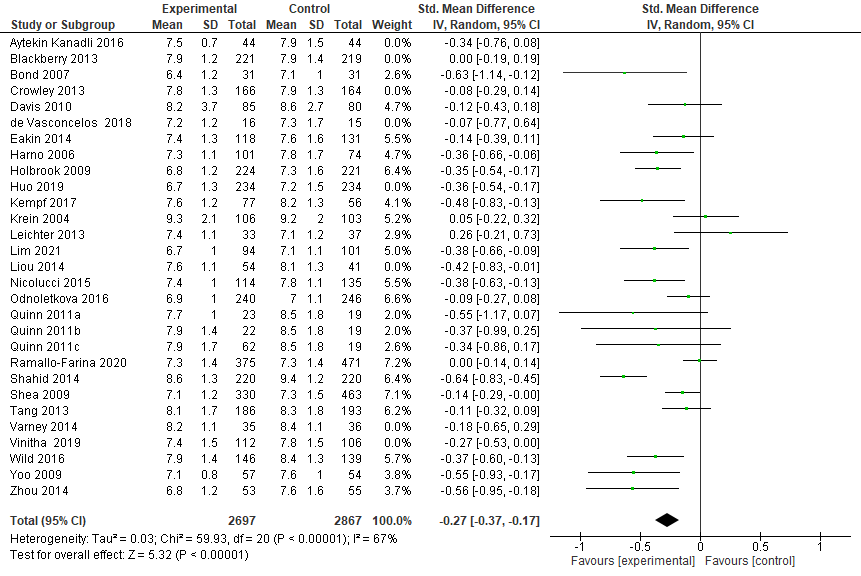


### 4.3 Forest Plot showing the Impact of remote interventions with remote coaching for risk factor modification on glycated haemoglobin A1c


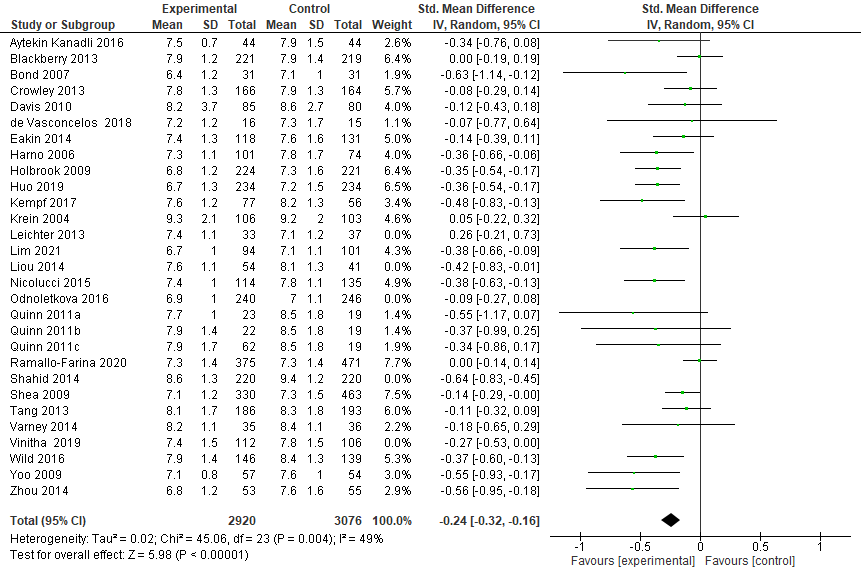


### 4.4 Forest Plot showing the Impact of remote interventions with remote consultation on glycated haemoglobin A1c


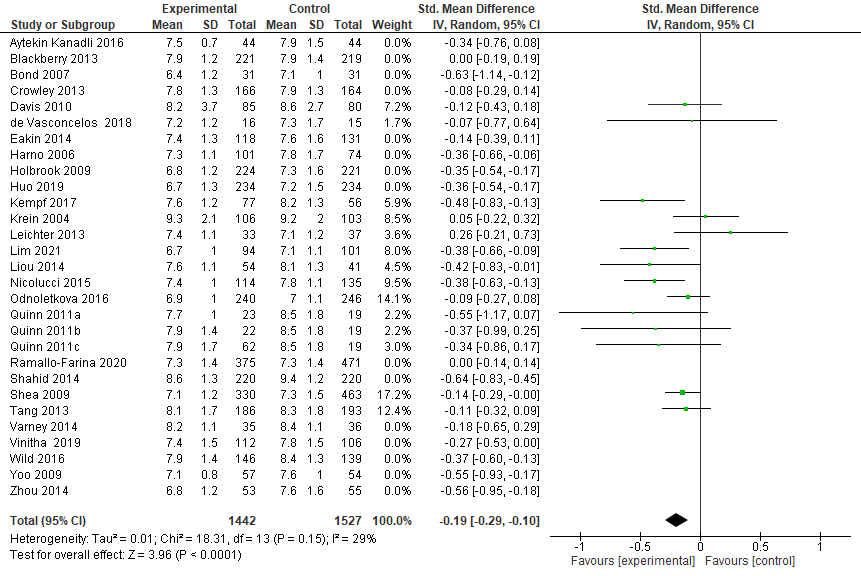


### 4.5 Forest Plot showing the Impact of remote interventions with remote pharmacological management on glycated haemoglobin A1c


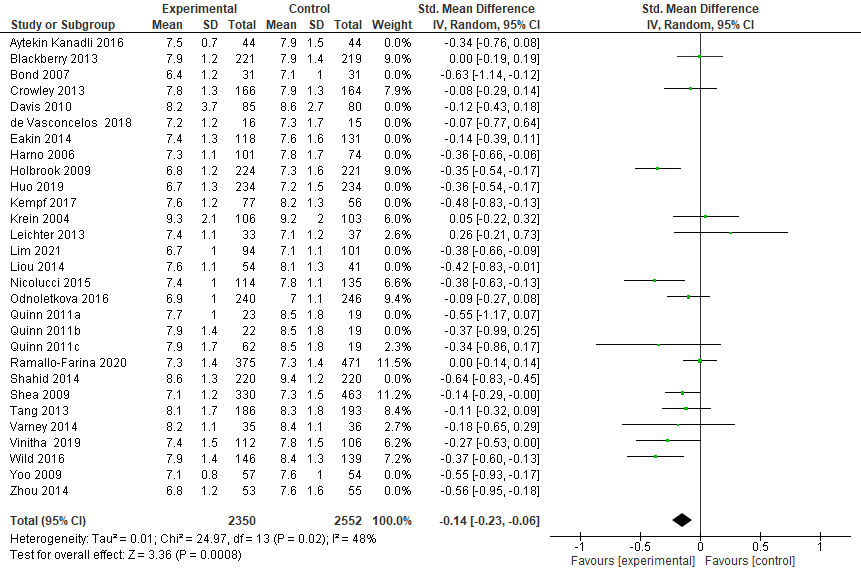


### 4.6 Forest Plot showing the impact of remote interventions with remote patient education on total cholesterol


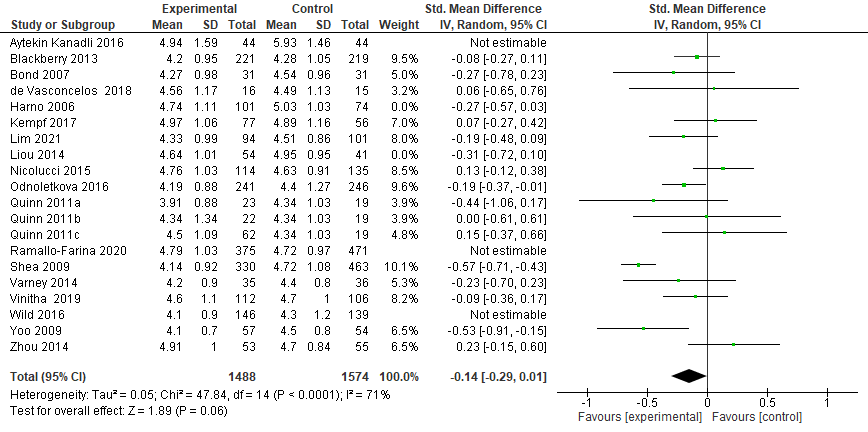


### 4.7 Forest Plot showing the Impact of remote interventions with remote monitoring of risk factors on total cholesterol


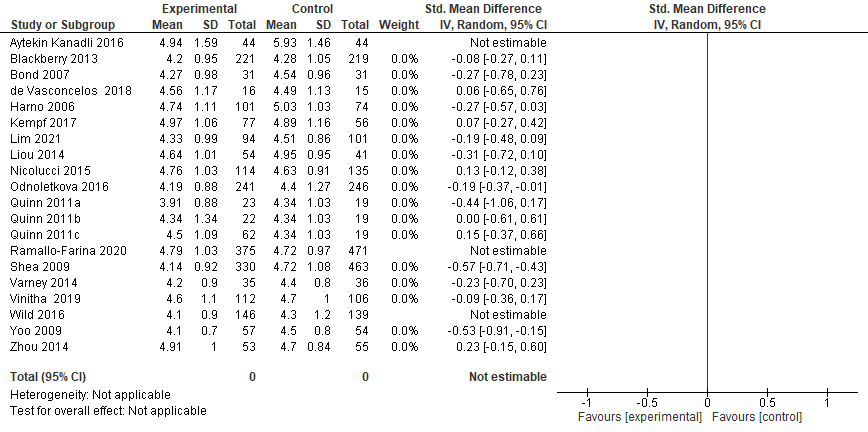


### 4.8 Forest Plot showing the Impact of remote interventions with remote coaching for risk factor modification on total cholesterol


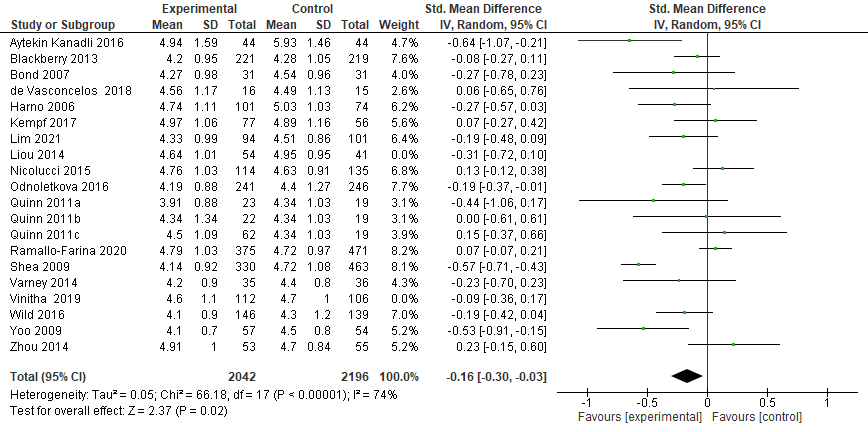


### 4.9 Forest Plot showing the Impact of remote interventions with remote consultation on total cholesterol


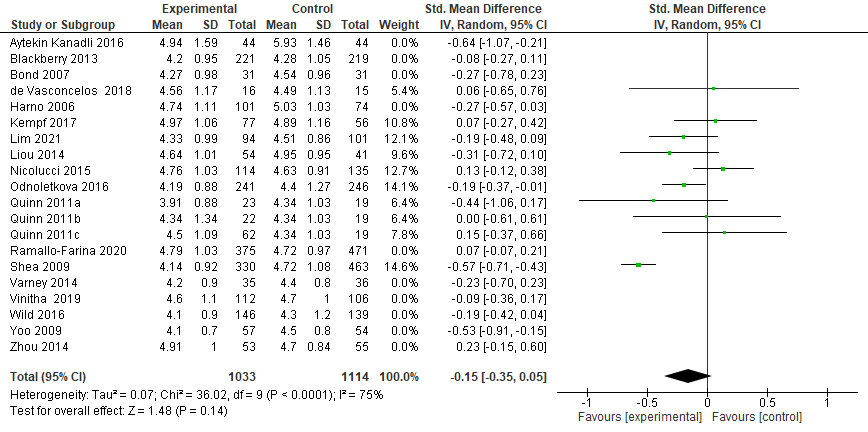


### 4.10 Forest Plot showing the Impact of remote interventions with remote pharmacological management on total cholesterol


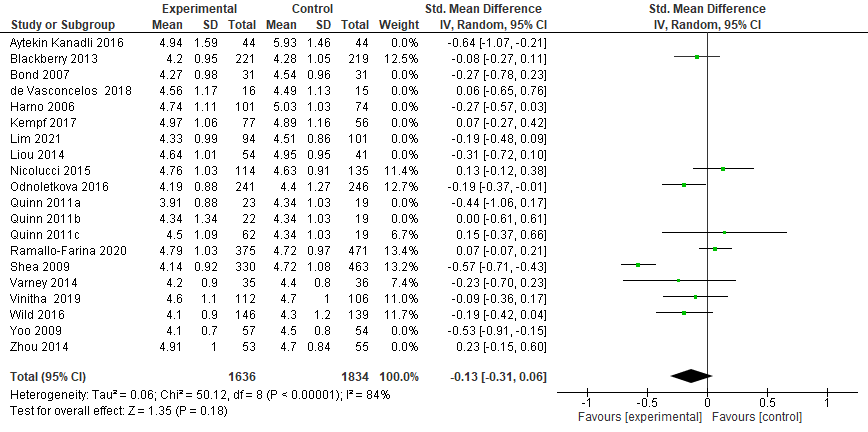
4

### 4.11 Forest Plot showing the impact of remote interventions with remote patient education on LDL-c


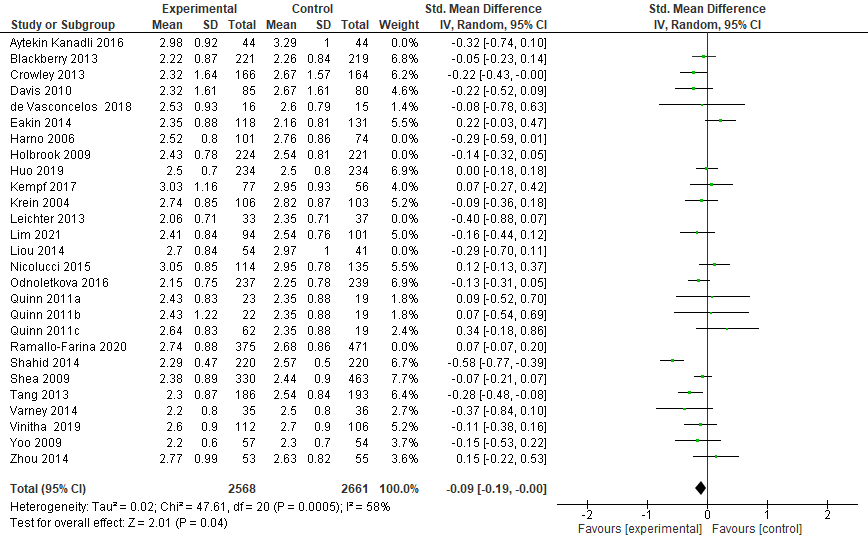


### 4.12 Forest Plot showing the Impact of remote interventions with remote monitoring of risk factors on LDL-c


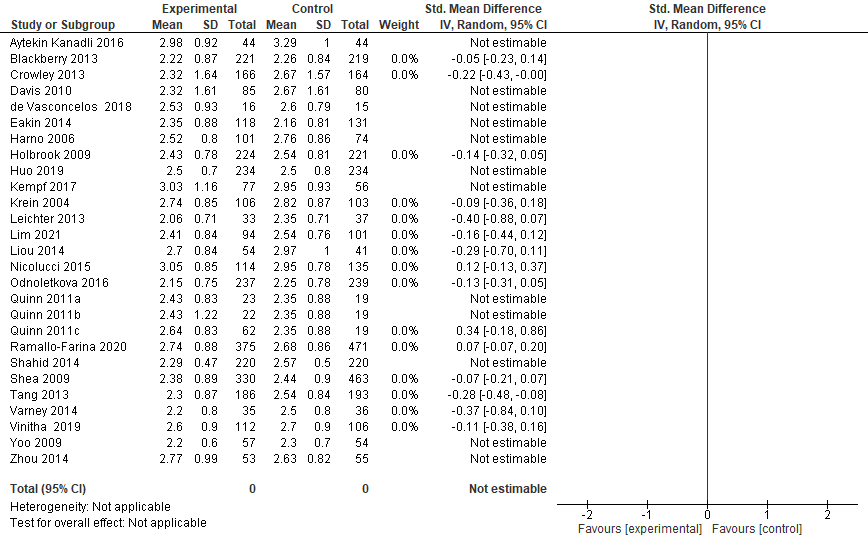


### 4.13 Forest Plot showing the Impact of remote interventions with remote coaching for risk factor modification on LDL-c


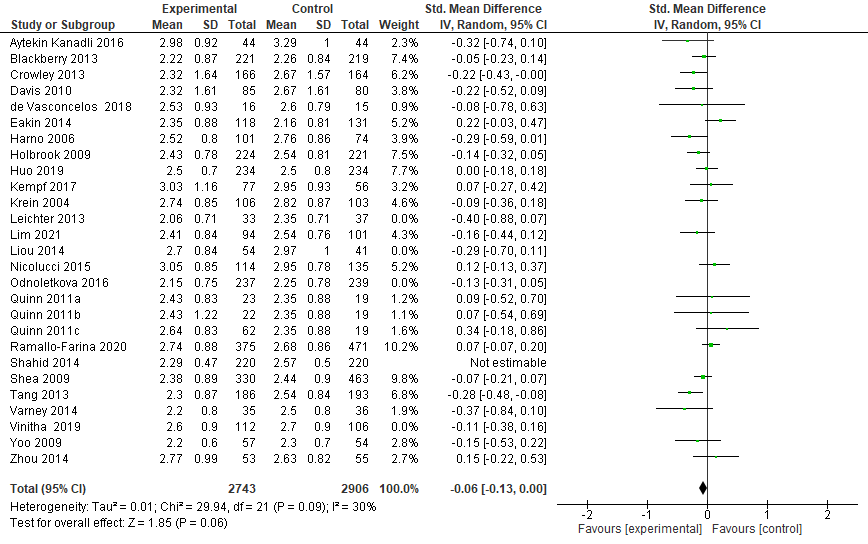


### 4.14 Forest Plot showing the Impact of remote interventions with remote consultation on LDL-c


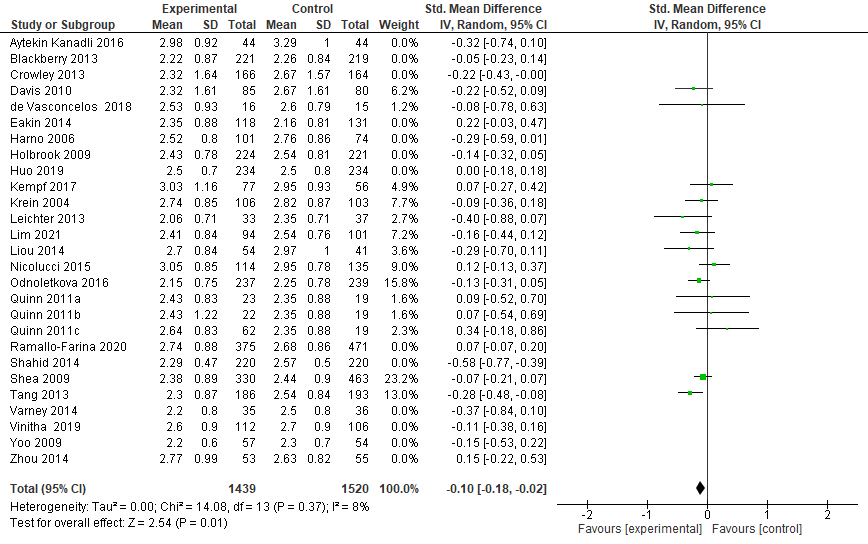


### 4.15 Forest Plot showing the Impact of remote interventions with remote pharmacological management on LDL-c


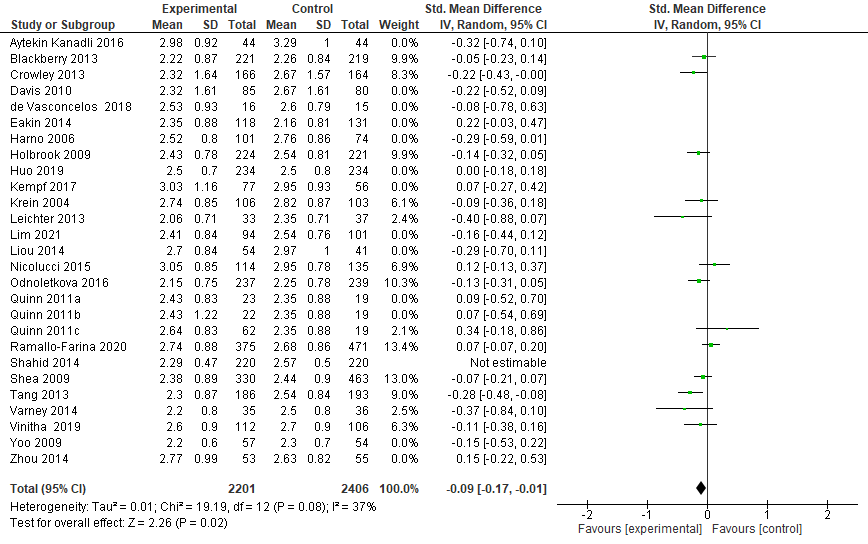


### 4.16 Forest Plot showing the impact of remote interventions with remote patient education on SBP


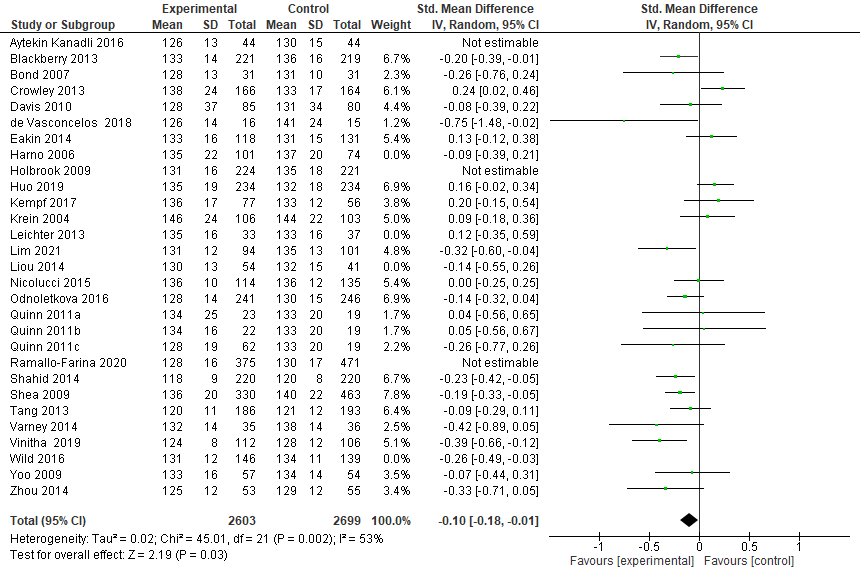


### 4.17 Forest Plot showing the Impact of remote interventions with remote monitoring of risk factors on SBP


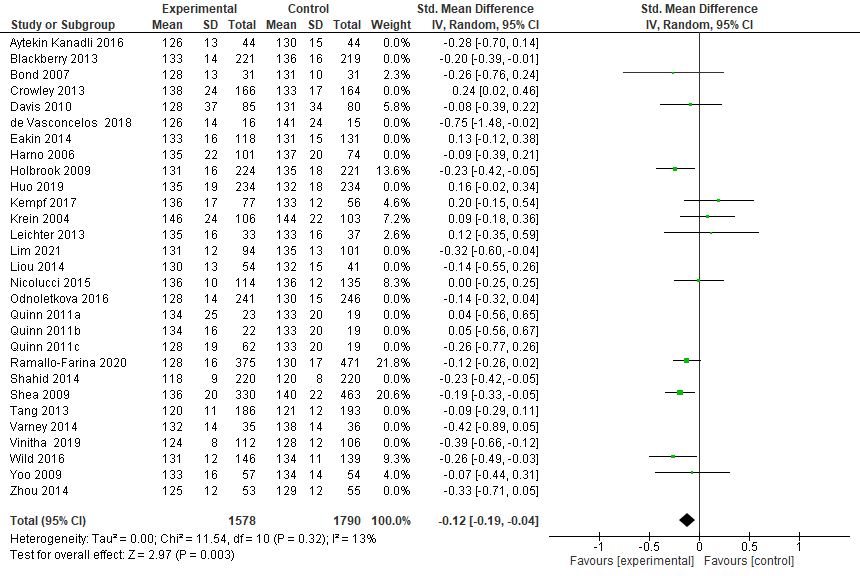


### 4.18 Forest Plot showing the Impact of remote interventions with remote coaching for risk factor modification on SBP


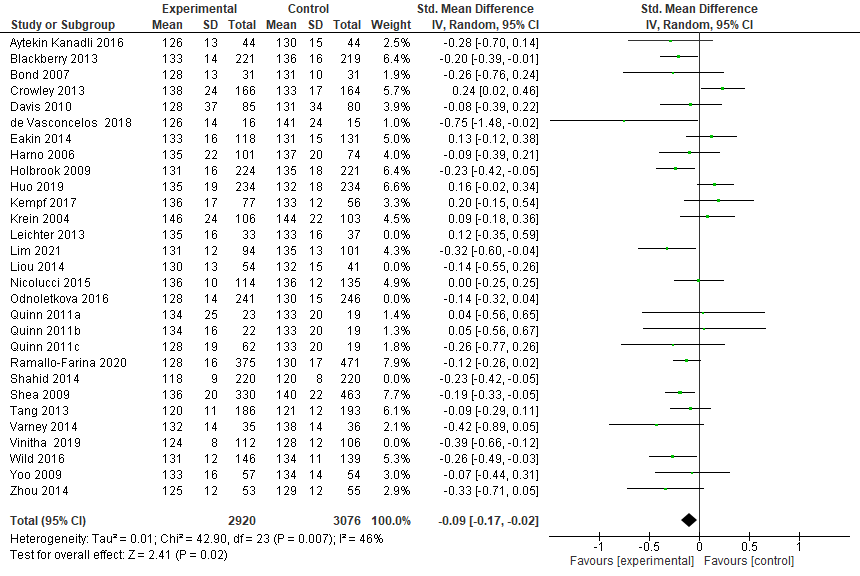


### 4.19 Forest Plot showing the Impact of remote interventions with remote consultation on SBP


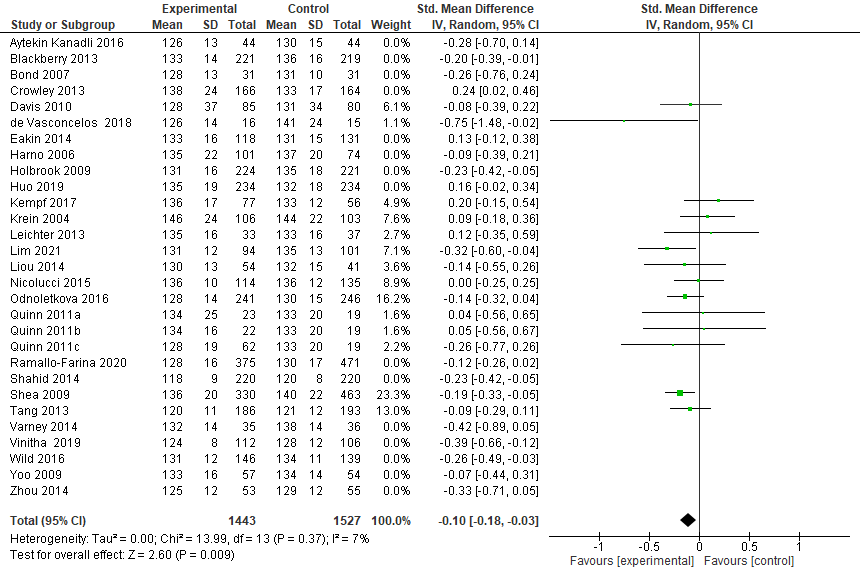


### 4.20 Forest Plot showing the Impact of remote interventions with remote pharmacological management on SBP


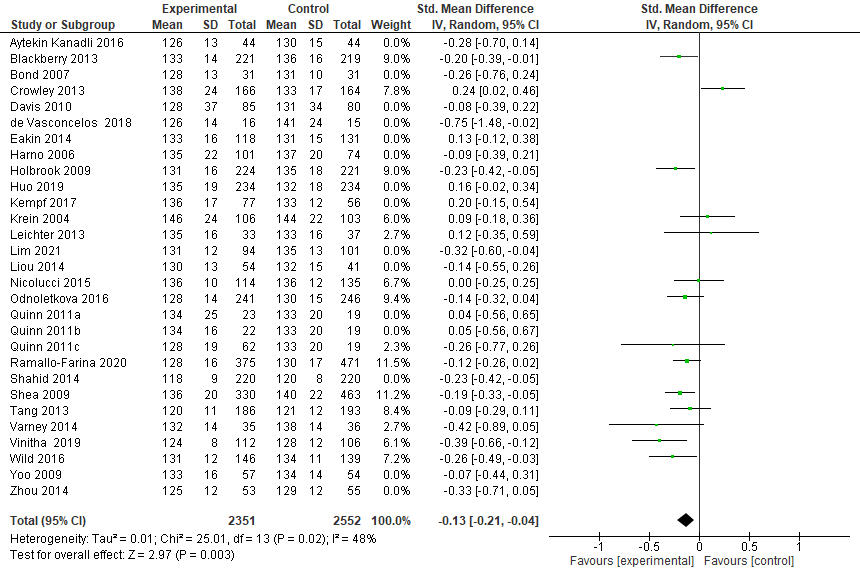


### 4.21 Forest Plot showing the impact of remote interventions with remote patient education on DBP


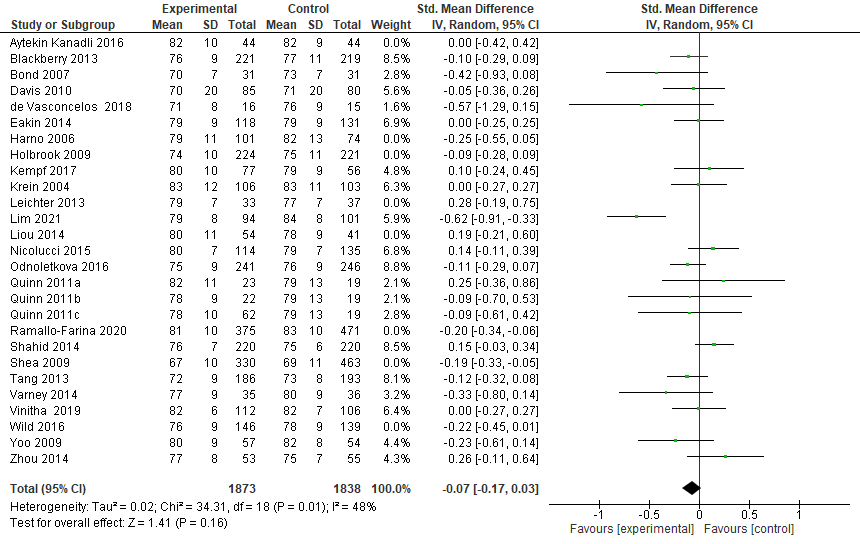


### 4.22 Forest Plot showing the Impact of remote interventions with remote monitoring of risk factors on DBP


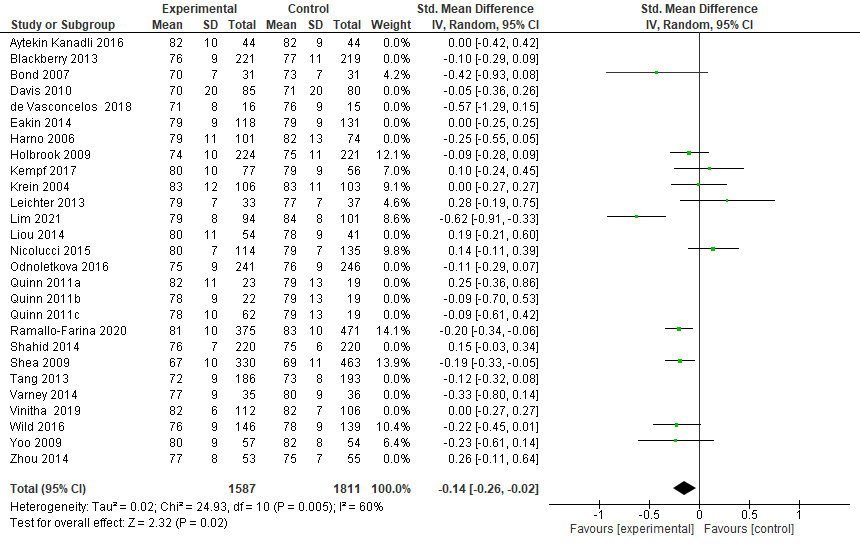


### 4.23 Forest Plot showing the Impact of remote interventions with remote coaching for risk factor modification on DBP


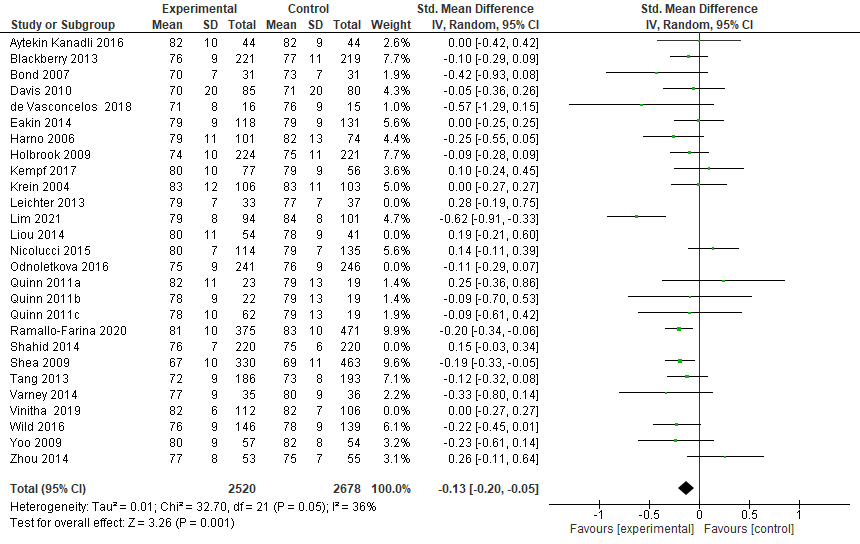


### 4.24 Forest Plot showing the Impact of remote interventions with remote consultation on DBP


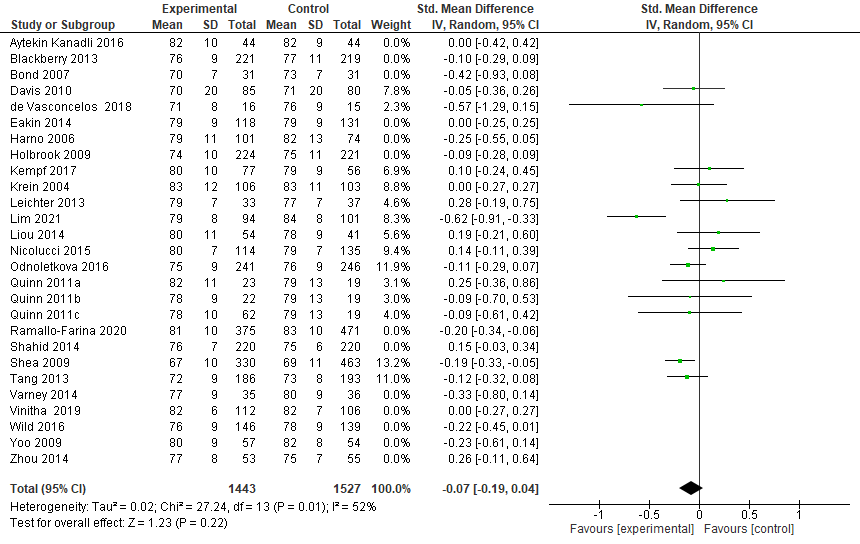


### 4.25 Forest Plot showing the Impact of remote interventions with remote pharmacological management on DBP


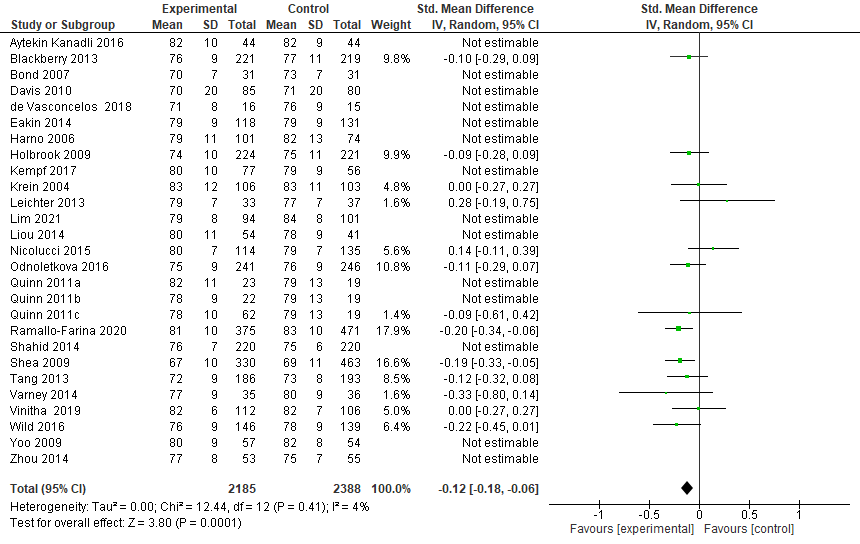


## 5. Subgroup analyses of primary outcomes based on high-risk population as per study inclusion criteria

### 5.1 Forest Plot showing the Impact of remote intervention on glycated haemoglobin A1c in a high-risk population


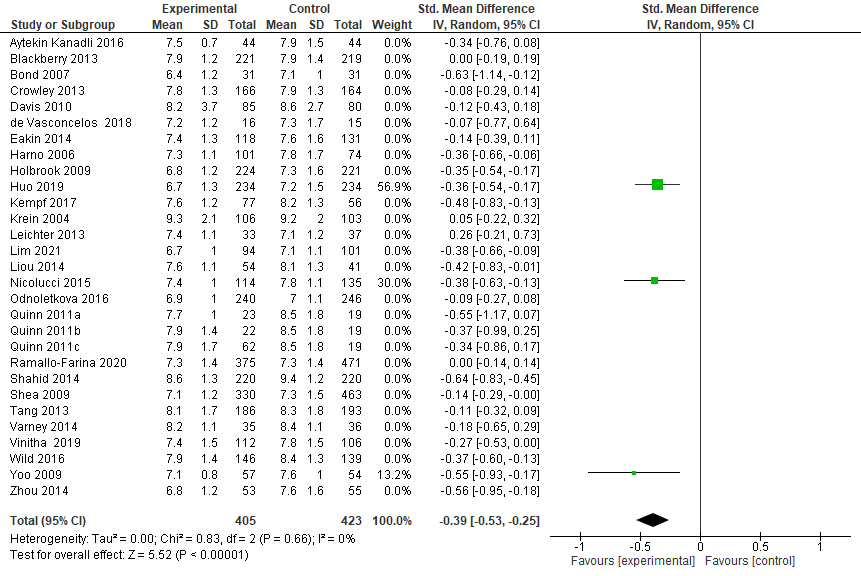


### 5.2 Forest Plot showing the Impact of remote intervention on LDL-c in a high-risk population


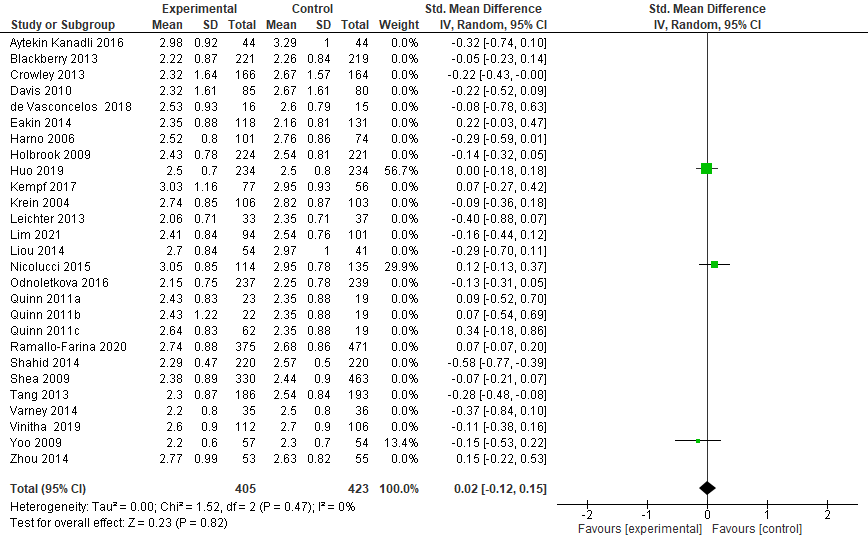


### 5.3 Forest Plot showing the Impact of remote intervention on SBP in a high-risk population


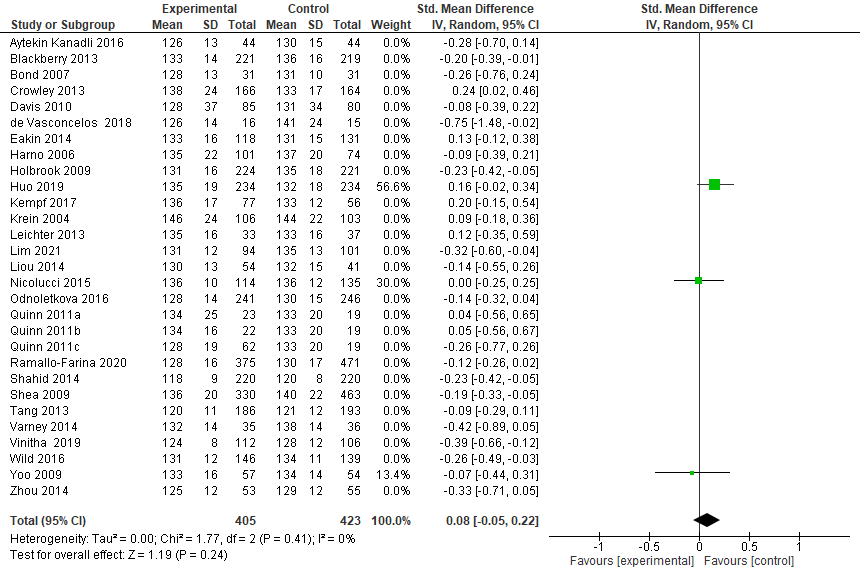


## 6. Secondary Outcomes

### 6.1 Forest plot showing the effect of remote management on overall adverse outcomes


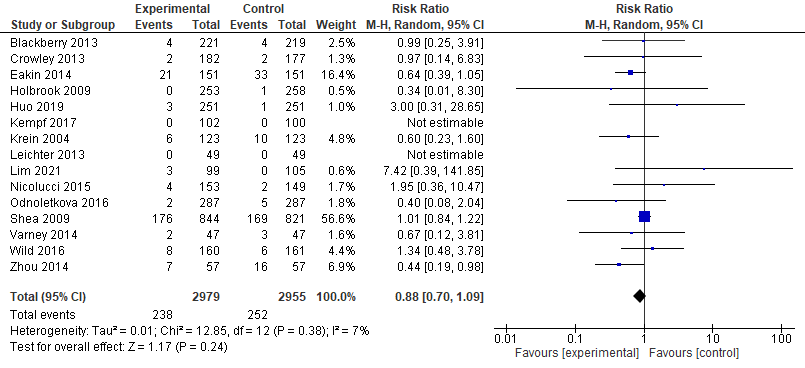


### 6.2 Forest plot showing the effect of remote management on mortality


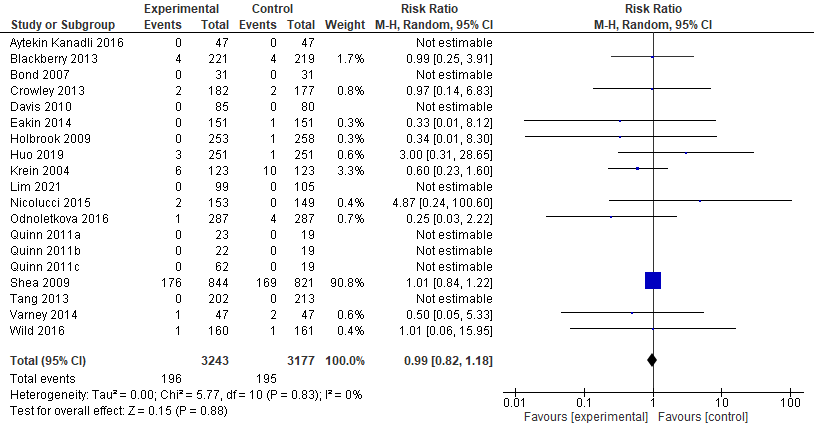


### 6.3 Forest plot showing the effect of remote management on hypoglycaemic episodes


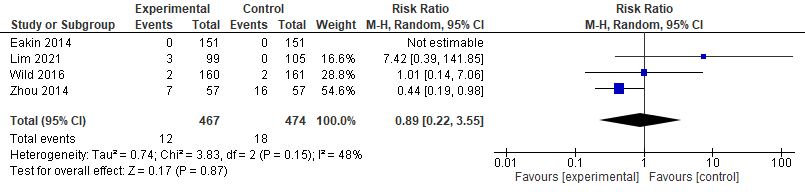


### 6.4 Forest plot showing the effect of remote management on hospitalisation


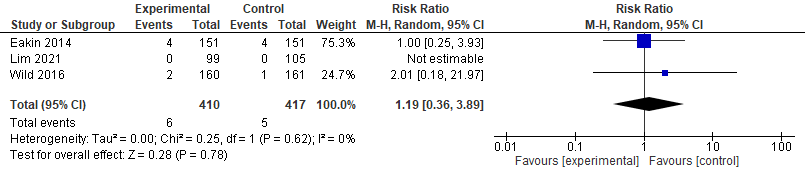


## 7. Funnel plots for secondary outcomes

### 7.1 Impact of remote intervention on overall adverse outcomes


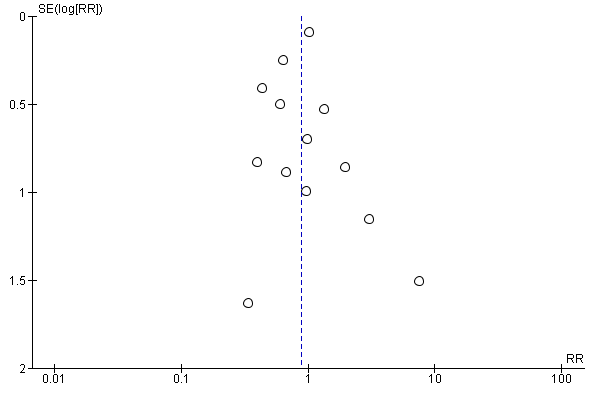


### 7.2 Impact of remote intervention on mortality


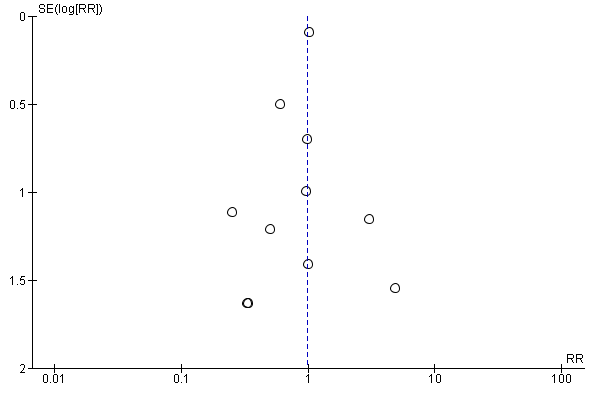


### 7.3 Impact of remote intervention on hypoglycaemic episodes


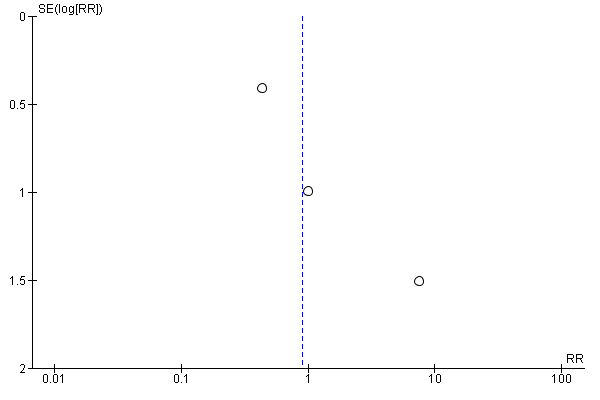


### 7.4 Impact of remote intervention on hospitalisation


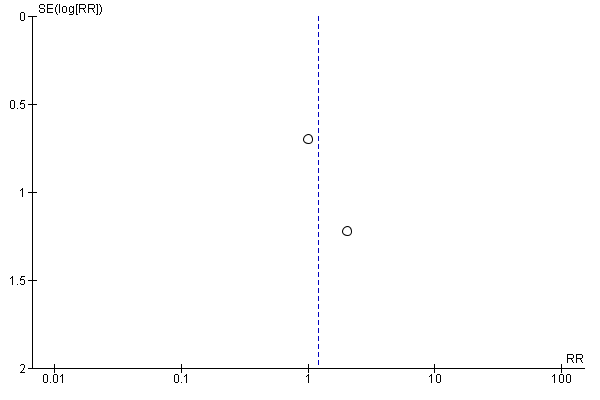

Supplement: Supplementary file 1 [file DataSheet_1.docx]
